# Unraveling the Role of Triplet–Triplet Annihilation and Photodegradation in Difluoroboron‐Based Organic Laser Gain Materials

**DOI:** 10.1002/anie.202509535

**Published:** 2025-09-16

**Authors:** Suman Kuila, Hector Miranda‐Salinas, Chunyong Li, Natalie E. Pridmore, Martin R. Bryce, Christel M. Marian, Andrew P. Monkman

**Affiliations:** ^1^ Department of Physics Durham University South Road Durham DH1 3LE UK; ^2^ Department of Chemistry Durham University South Road Durham DH1 3LE UK; ^3^ Institute of Theoretical and Computational Chemistry Faculty of Mathematics and Natural Sciences Heinrich Heine University Düsseldorf D‐40204 Düsseldorf Germany

**Keywords:** Aggregation, Amplified spontaneous emission, Delayed fluorescence, Photochemistry, Triplet–triplet annihilation

## Abstract

In this study, we investigate the triplet exciton dynamics of a series of difluoroboron‐based organic gain molecules. We synthesized three previously reported molecules from the difluoroboron family and examined their photophysical properties using time‐resolved emission spectroscopy and high‐level theoretical calculations. Our results reveal that emission from these materials arises predominantly from the singlet manifold via prompt and triplet–triplet annihilation (TTA)‐driven delayed fluorescence, rather than from phosphorescence, challenging the earlier assumptions of amplified spontaneous emission (ASE) originating from the triplet manifold. In highly concentrated solutions, the emission shows strong resemblance to that of the crystalline phase, confirming its origin from aggregate singlet states rather than monomeric pathways. Further, the materials are prone to photodegradation, which gives rise to new high‐energy fluorescence and phosphorescence bands adding to the complexity of the photophysics of this family of materials.

## Graphical abstract

Amplified spontaneous emission (ASE) in difluoroboron‐based gain molecules is shown to originate from aggregated singlet states (S_1_), contradicting its previously assumed phosphorescent origin. Additionally, the delayed emission arises from a triplet–triplet annihilation upconversion (TTA‐UC) mechanism rather than a thermally activated (TADF) process, with rapid photodegradation further complicating the excited‐state dynamics and emphasizing the need for improved molecular design.

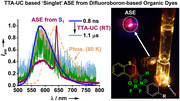

## Introduction

Understanding the formation mechanisms and stabilization of triplet excitons in organic molecules is crucial for controlling many photophysical processes, which are essential for advancing state‐of‐the‐art materials used in electroluminescence, photovoltaics, biological probes, and photocatalysis.^[^ ^1^, ^2^, ^3^, ^4^, ^5^, ^6^, ^7^, ^8^ ^]^ However, in conventional organic chromophores, formation and emissive decay of the excited triplet states are highly inefficient as a change in spin angular momentum is forbidden.^[^ ^9^, ^10^ ^]^ This has a critical impact in electroluminescence applications such as organic light‐emitting diodes (OLEDs), where electrical excitation produces singlet (S) and triplet (T) excited states in a 1:3 ratio upon random recombination of injected electrons and holes.^[^ ^5^ ^]^ Therefore, methods to upconvert triplet excitons to singlet states, as opposed to converting singlets to triplets with phosphorescent emitters,^[^ ^11^ ^]^ are essential for practical device applications. Upconversion has been achieved primarily through two mechanisms: triplet–triplet annihilation (TTA) and thermally activated delayed fluorescence (TADF).^[^ ^12^, ^13^, ^14^, ^15^ ^]^ In TTA, the theoretical maximum internal quantum efficiency a device can achieve is 62.5% but in reality, it lies between 20% and 40%, because two triplet excitons must combine to form one higher‐energy singlet exciton, leading to inherent energy losses.^[^ ^16^ ^]^ In contrast, TADF offers the potential for 100% efficiency, as it involves the thermal activation of triplet excitons to singlet states, allowing all excitons to contribute to light emission.^[^ ^5^ ^]^ Consequently, emitters that utilize the TADF mechanism are particularly promising for OLED applications, as they maximize efficiency and brightness, making them more suitable for high‐performance displays and lighting technologies.^[^ ^1^, ^17^ ^]^ The same mechanism could also greatly enhance electrically pumped organic‐based lasers. Current injection into the emitter layer creates statistically 75% triplets and therefore, a very fast and efficient triplet harvesting would greatly reduce lasing threshold currents.^[^ ^18^, ^19^, ^20^, ^21^, ^22^, ^23^ ^]^ By reducing the lasing threshold, various excited‐state annihilation processes (such as triplet–triplet annihilation, singlet–triplet annihilation, and triplet–polaron annihilation) are also alleviated, which in turn helps to reduce the threshold current further.^[^ ^24^ ^]^ Thus, to develop highly efficient solid‐state organic semiconductor lasers, ultrafast triplet harvesting becomes imperative.^[^ ^25^, ^26^, ^27^, ^28^ ^]^

**Figure 1:**
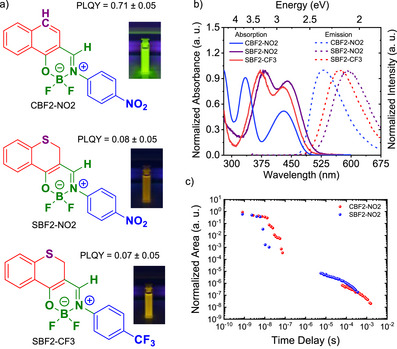
Basic photophysical properties of the three molecules studied here. a) Molecular structure of the boron‐difluoride chromophores; photographs of DCM solution of the three chromophores under 365 nm UV‐lamp are also shown. b) Absorption and steady‐state emission spectra in 1 × 10^−5^ M DCM solution, showing increasing charge‐transfer strength from **CBF2‐NO2** to **SBF2‐CF3** to **SBF2‐NO2**. c) Time‐resolved decay of degassed **SBF2‐NO2** and **CBF2‐NO2** in DCM (*λ*_exc _= 355 nm, [*c*] = 1 × 10^−5^ M) showing prompt and delayed emission. Photoluminescence quantum yield (PLQY) was measured in air‐saturated DCM solution (*λ*_exc _= 450 nm, [*c*] = 1 × 10^−5^ M).

So far, primarily fluorescent organic molecules have been used as gain materials as they offer an excellent platform for a 4‐level lasing scheme to achieve efficient population inversion at low current, but triplet states are sacrificed.^[^ ^29^, ^30^, ^31^, ^32^, ^33^, ^34^, ^35^, ^36^, ^37^ ^]^ However, triplet excited states offer extended lifetimes, potentially yielding longer‐lived metastable states and enhancing the population inversion. But the triplet states’ very long‐lived nature is also detrimental for lasing due to competing bimolecular annihilation losses and further gain losses through excited triplet state absorption, which generally overlaps with the stimulated emission.^[^ ^19^ ^]^ Therefore, two scenarios for lasing involving the triplet states can be envisaged: either using fast TADF to convert the triplets into a population inversion in the singlet manifold, or fast, efficient stimulated phosphorescence from a triplet population inversion giving rise to optical gain. Recent attempts made by various groups to use triplet excitons as organic gain materials have been reported; however, the fundamental understanding of such processes is far from clear.^[^ ^38^, ^39^, ^40^, ^41^, ^42^, ^43^, ^44^, ^45^, ^46^ ^]^ For example, Fu and coworkers have reported a series of difluoroboron‐based molecules that they suggest give phosphorescence amplified spontaneous emission at room temperature. A slight variation in the acceptor unit of their molecule, e.g., changing a ─PhNO_2_ to a ─PhCF_3_ (Figure 1) reportedly changes the emission characteristics drastically and room temperature phosphorescence (RTP) in **SBF2‐NO2** is replaced by TADF in **SBF2‐CF3** (Figure 1).^[^ ^41^, ^45^ ^]^ Therefore, only a subtle change in the acceptor strength seemingly drives fundamental changes in the triplet dynamics of the materials so dramatically. A thorough spectroscopic and computational investigation to account for this behavior will improve our understanding of this class of materials, which could give rise to a new form of lasing. Here, we derive a mechanistic understanding of boron‐difluoride‐based organic gain molecules, synthesizing a series of previously reported materials^[^ ^41^, ^45^ ^]^ to elucidate their triplet exciton dynamics by time‐resolved emission spectroscopy and theoretical calculations.

## Results and Discussion

### The Boron‐Difluoride Chromophores Investigated in this Work

Details of the synthesis of the three boron‐difluoride compounds studied are given in the Supporting Information, along with their characterization and sample preparation methods. Molecular structures and basic photophysical properties in dichloromethane (DCM) solution are shown in Figure 1. Figures 1 and S1 show steady‐state absorbance and PL characteristics of the three molecules studied in this work. In line with the previous reports, the strength of charge‐transfer characteristics increases from **CBF2‐NO2** to **SBF2‐NO2**, commensurate with stronger donor strength of the thioether‐based donor unit, resulting in larger solvatochromic shifts. Further, photoluminescence quantum yield (PLQY) was measured in methylcyclohexane and (DCM) for **CBF2‐NO2** 0.77 (0.71)**, SBF2‐NO2** 0.29 (0.08), and **SBF2‐CF3** 0.11 (0.07). This shows that only **SBF2‐NO2** is strongly affected by solvent polarity, which we attribute to increased radiative decay in nonpolar MCH, potentially indicating a small change in the CT character of the S_1_ state and delayed emission (Figure S1). The molecular packings within crystals of **SBF2‐NO2** and **CBF2‐NO2** are given in Figures S2, S3 and Table S1. These surprisingly are very different. Whereas the **SBF2‐NO2** forms head‐to‐head stacks (nitrobenzene groups adjacent to the naphthalene groups), the **CBF2‐NO2** forms head‐to‐tail stacks (neighboring nitrobenzene groups). In both cases, the ─BF_2_ groups alternate left‐side and right‐side along the stacks. These packing effects significantly influence the optical properties as discussed later (vide infra).

### Theoretical Modeling of the Investigated Molecules

**SBF2‐NO2, CBF2‐NO2,** and **SBF2‐2MeNO2** were previously studied theoretically by Fu and coworkers^[^ ^41^, ^44^, ^45^ ^]^ who carried out calculations at the B3LYP/6‐31G* density functional theory (DFT) and time‐dependent DFT (TDDFT) levels, employing the polarizable continuum model (PCM) to represent the different environments studied experimentally, i.e., polystyrene (PS) film, dichloromethane (DCM) solution, and glycol/methanol mixtures.

In this study, we employed a higher level of theory: PBE0^[^ ^47^, ^48^ ^]^/TZVP^[^ ^49^ ^]^ (TD)DFT geometry optimizations and vibrational frequency calculations were performed using Gaussian16^[^ ^50^ ^]^ while electronic excitation energies and spin–orbit‐free properties were determined at the DFT/MRCI level of theory,^[^ ^51^, ^52^ ^]^ a semi‐empirical multireference configuration interaction (MRCI) approach based on BH‐LYP^[^ ^53^, ^54^ ^]^ Kohn–Sham DFT molecular orbitals. To model a DCM environment, PCM point charges^[^ ^55^, ^56^ ^]^ from the (TD)DFT runs were exported from Gaussian16 and included in Turbomole.^[^ ^57^ ^]^ Kohn–Sham DFT calculations preceded the DFT/MRCI runs. To compare the theoretical results with experiments in PS film or Zeonex hosts, vacuum conditions were assumed satisfactory. Phosphorescence rate constants were determined using DFT/MRSOCI wavefunctions employing atomic spin–orbit mean‐field integrals, as implemented in the spin–orbit coupling program Spock.^[^ ^58^, ^59^ ^]^ intersystem crossing (ISC) and reverse intersystem crossing (rISC)2 rate constants were computed according to Fermi's golden rule in the Condon approximation using the electronic spin–orbit coupling matrix element (SOCME) of the DFT/MRCI wavefunctions at the minimum geometry of the initial state and a Fourier transform approach^[^ ^60^, ^61^ ^]^ for evaluating the vibrational overlaps. Initial state populations at 77 and 298 K were assumed to follow Boltzmann distributions.

Starting with **SBF2‐NO2**, two rotamers with B–N–C–C torsion angles θ ≅ ±35° (Figure S4) were identified in the electronic ground and excited states. In **SBF2‐2MeNO2**, steric hindrance forces the di‐methylated nitrobenzene (DMNB) group to take a nearly perpendicular orientation with respect to the sulfide‐substituted difluoroboron (SBF_2_) moiety, which itself is not planar but substantially puckered. Only one rotamer can be formed in this case. Because the rotamers of **SBF2‐NO2** were found to have nearly identical energies (R2 < R1 by 20 meV), dipole moments and transition rate constants, only one rotamer of **SBF2‐CF3** was investigated in detail. As an initial verification, we calculated the absorption spectra of all molecules in vacuum and DCM, shown in Figure S5, which are found to be in excellent agreement with the experimentally measured spectra (Figures 1 and S1).

**Figure 2:**
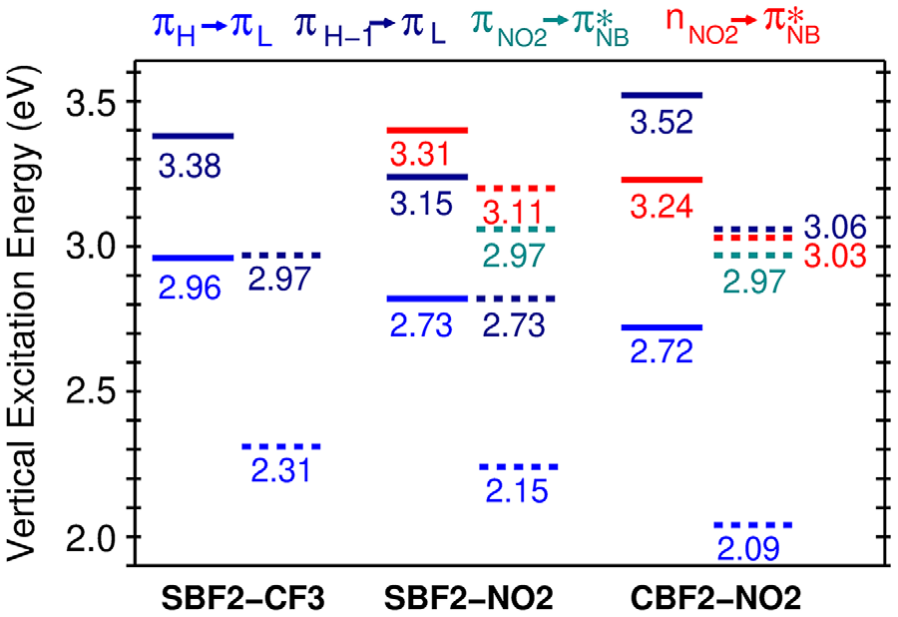
Vertical DFT/MRCI excitation energies of the three molecules studied here, calculated at the respective ground‐state minimum geometry in DCM solution. Singlet states are symbolized by solid lines and triplet states by dashed lines.

At variance with the computational results of Yu et al.,^[^ ^41^ ^]^ we find that the S_1_ and S_2_ states of **SBF2‐NO2** originate mainly from (π_H_→π_L_) and (π_H‐1_→π_L_) excitations, respectively (Figure 2 and Tables S2 (R1) and S3 (R2)). The first singlet excitation of nπ*‐type (S_3_ in the Franck–Condon (FC) region) is located on the nitrobenzene residue and therefore designated (n_NO2_→ π*_NB_) (Figures S6 and S7). The wavefunctions of the T_1_ and T_2_ states are composed of a nearly 1:1 mixture of the (π_H_→π_L_) and (π_H‐1_→π_L_) configurations in the FC region. The singlet excited states exhibit partial CT character and experience a small redshift in DCM with respect to a nonpolar environment, by about 0.05–0.10 eV (Tables S2 (DCM) and S4 (vacuum)). This agrees very well with what we observe experimentally. Moreover, the relative intensities of the S_1_ and S_2_ absorption bands are inverted by the DCM environment (Figure S5). The calculated absorption maxima of **SBF2‐NO2** (S_1_ 437 nm, S_2_ 385 nm, vacuum; S_1_ 455 nm, S_2_ 393 nm in DCM) agree well with measured peak positions (445 and 380 nm in DCM). In energetic proximity to the S_2_ state, we find the optically dark, local singlet (S_3_) and triplet (T_4_) excitations of n_NO2_→ π*_NB_ character, located on the nitrobenzene (NB) unit with minor π_Ph_→ π*_NB_ admixtures (Figure 1 and Table S2). Furthermore, the triplet T_4_ π_NO2_→ π*_NB_ state lies below S_2_ in the FC region. Thus, the (n_NO2_→ π*_NB_) states may play an important role in the emission decay due to their substantial relaxation energies.

The absorption spectrum of **SBF2‐CF3** shows minimal solvent shift, as determined experimentally (Figure S1c), whereas the relative peak intensities of the S_1_ and S_2_ absorptions invert in DCM compared to vacuum (Figure S5). Peak maxima are calculated at 415 and 368 nm in vacuum and at 419 and 367 nm in DCM, in excellent agreement with the experimentally observed bands at 425 and 370 nm in DCM (Figures 1 and S5). The composition of the S_1_, S_2_, T_1_, and T_2_ wavefunctions resembles those of **SBF2‐NO2**, but the CF_3_ substituent does not participate in the excitations and therefore the CT contributions are reduced (Figures S10 and S11), and the excitations are blue‐shifted (Figure 2). The S_3_ and T_3_ states of **SBF2‐CF3** originate from a mixture of configurations with dominant π_H‐3_→π_L_ excitations (Figure S10 and Table S8).

The annelated rings of the nonsulfur‐containing compound, **CBF2‐NO2,** are essentially planar. The two rotamers exhibit nearly identical properties (their energies differ by less than 0.001 eV). The following discussion therefore focuses on rotamer R1. Its S_1_ (π_H_→π_L_) state closely resembles its **SBF2‐NO2** counterpart with regard to electronic structure (cp. Figures S6 and S12) and vertical excitation energy (Figure 2). In agreement with experimental observations, the π_H‐1_→π_L_ excitation strongly blue shifts and forms the S_3_ (π_H‐1_→π_L_) state of **CBF2‐NO2** in the FC region. The corresponding triplet state (T_2_ in **SBF2‐NO2**, T_4_ in **CBF2‐NO2**) is shifted to higher energies as well. Besides the reduced SOC in the nonsulfur‐containing compound, the blue shift of the T(π_H‐1_→π_L_) state is one of the reasons underlying the altered photophysics of **CBF2‐NO2**. Although the S_1_’ (n_NO2_→ π*_NB_) transition is optically dark, the nπ* state might nevertheless be populated upon photoexcitation with a 355 nm laser because it is located at the lower edge of the bright S_3_ (π_H‐1_→π_L_) transition.

**Figure 3:**
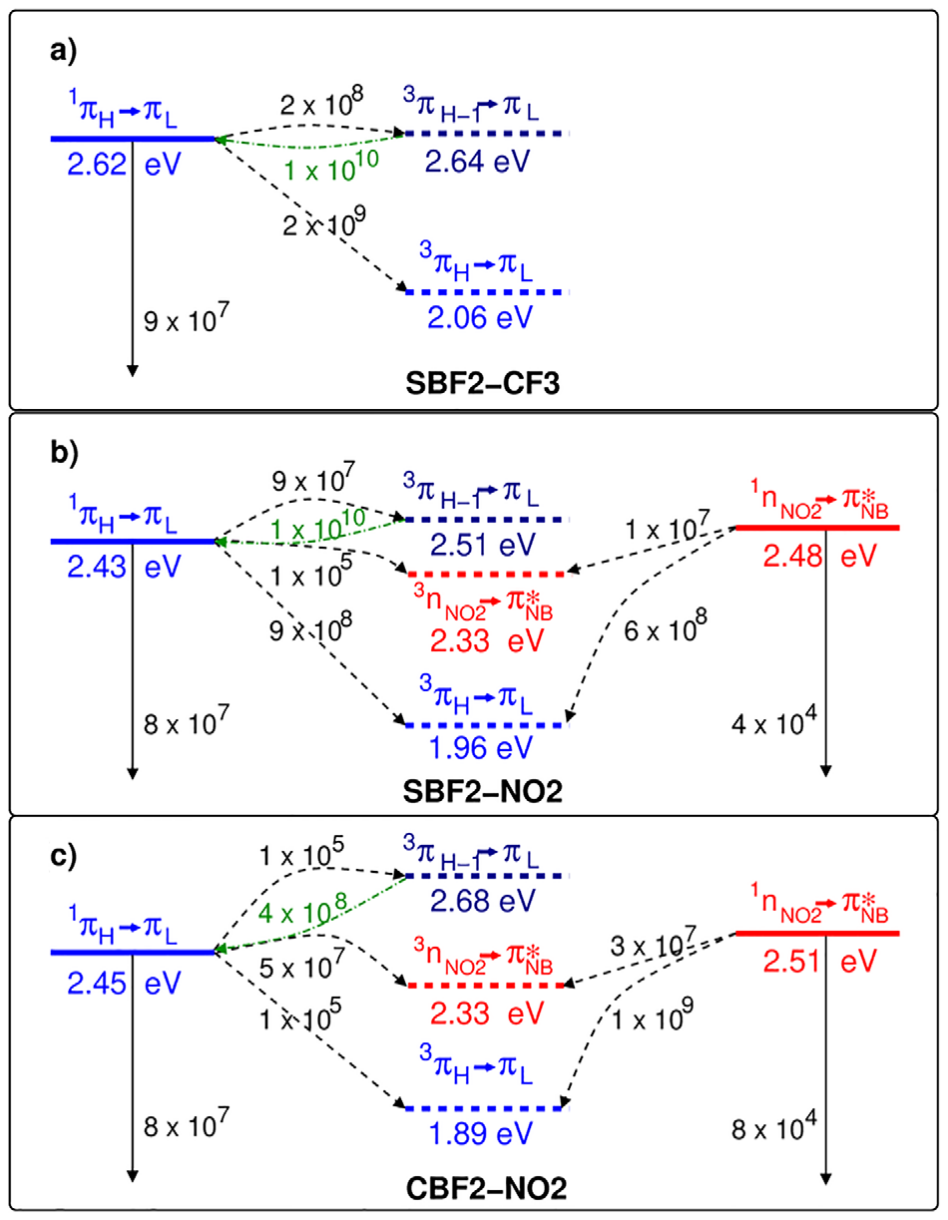
Adiabatic DFT/MRCI excitation energies of the three molecules studied here in DCM solution as well as transition rate constants (s^−1^). ISC and rISC rate constants were calculated for a temperature of 298 K. Corresponding rate constants for a temperature of 77 K and computed phosphorescence rate constants are given in Tables S5, S9, and S12.

It is tempting to deduce the electronic structures of the lowest‐lying adiabatic states from the wavefunction characteristics in the FC region for these compounds; however, the wavefunction compositions change markedly upon geometry relaxation in the excited states. At their respective minimum geometries, the T_1_ and S_1_ wavefunctions of **SBF2‐NO2** are dominated by the π_H_→π_L_ configuration. The charge distribution in the S_1_ state (S_1_ geometry) (Figure S8) reveals minor (SBF_2_→NB) CT contributions. At the DFT/MRCI level, the lowest energy S_1_ state is of ππ* character below the S_2_ nπ* state (Figure 3), inverted compared to the (lower level) DFT calculations of Yu et al.^[^ ^41^ ^]^ The excitation in the T_1_ state (T_1_ geometry) is more localized on the SBF_2_ unit, with small charge shifts in the (SBF_2_←NB) direction (Figure S8). Nevertheless, the minimum nuclear arrangements at the S_1_ and T_1_ minima closely resemble one another. The T_1_ (π_H_→π_L_) excitation forms the global minimum on the first excited triplet hypersurface in all solvent environments. In contrast to the TDDFT results of Li et al.,^[^ ^44^ ^]^ our calculations always place T_2_ adiabatically higher in energy than S_1_ (Figure 3b). In addition, the zero‐point vibrational energy of the T_2_ state is higher than that of S_1_. However, considering the error margins of the respective computational methods, the energetic order in Li et al.’s calculation could well be reversed.^[^ ^44^ ^]^ In any case, the S_1_ and T_2_ potentials undergo a crossing along the reaction coordinate connecting the two minima. At the T_2_ geometry, the T_1_ and T_2_ wavefunctions are strongly mixed linear combinations of the π_H‐1_→π_L_ and π_H_→π_L_ excitations, similar to their compositions at the ground‐state geometry. Although the different densities of S_1_ and T_1_ are visually almost indistinguishable (Figure S8), their electronic structures are sufficiently different to allow their mutual SOC to be sizeable (component averaged value 4.36 cm^−1^ for R1), resulting in ISC rate constants in the order of 10^8^–10^9^ s^−1^, depending on the temperature (Figure 3 and Table S5).

The static dipole moment increases upon π_H‐1_→π_L_ and π_H_→π_L_ excitation, but genuine CT (SBF_2_→NB) excitations are not found among the lowest‐lying electronic states. The S_3_ and T_4_ (n_NO2_→ π*_NB_) states form the lowest‐excited states (denominated S_1_’ and T_1_’, respectively) at their optimized geometries. It is not clear whether the S_1_’ state is effectively populated, however, because it lies quite high in the FC region and its formation from the S_1_ minimum requires substantial (>0.7 eV) reorganization energy. If populated, it rapidly decays (nanosecond regime) to the global T_1_ minimum. This applies equally to **SBF2‐NO2** in DCM solution and in a nonpolar environment (Figure 3 and Tables S5–S7). In vacuum, the S_1_’ potential well forms the global minimum on the first excited singlet hypersurface (Table S4). In the highly twisted DMNB derivative, **SBF2‐2MeNO2**, the optically dark ^1^(n_NO2_→π) excitation adiabatically forms the lowest excited singlet state even in DCM (Figures S14, S15 and Tables S13, S14), while the ^3^(π_H_→π_L_) structure continues to represent the global T_1_ minimum. Consequently, we expect the PLQY of this compound to be much lower than for **SBF2‐NO2**.

It is evident that all members of this family of compounds exhibit, at best, partial CT character. Fluorescence (≅10^8^ s^−1^) and ISC (≅10^8^–10^9^ s^−1^) are nearly competitive decay processes of the S_1_ population in **SBF2‐NO2** (Figure 3 and Tables S5–S7). Due to the large vibrational overlaps and sizeable electronic SOCMEs, direct S_1_ to T_1_ ISC is fast despite the energy gap of about 0.45 eV in DCM and 0.60 eV in nonpolar media. This is fully in line with our quantum yield determinations of the sulfur‐containing compounds. Mediation of the ISC by the energetically proximate T_2_ state is not required. However, according to our computational study, S_1_ to T_2_ ISC is a thermally activated process and therefore strongly temperature dependent, effectively zero at 77 K and 3 times slower than S_1_ to T_1_ ISC at 298 K for R1 (Table S5). Any T_2_ population can decay either by rapid IC to the T_1_ state or by rISC to the S_1_ state. The IC rate constants have not been computed, but rate constants of the order of 10^10^ s^−1^ are obtained for rISC in DCM solution and in nonpolar environments (Figure 3 and Tables S5–S7). This is a very fast rISC rate and may be competitive with IC to T_1_, but we note that the potential initial T_2_ population will also be small compared to that formed directly in T_1_; thus, rISC from T_2_ will at best only contribute a very minor contribution to S_1_ emission even at room temperature. Because of the sulfur contributions to SOC, the T_1_ phosphorescence rate constant is higher than for typical ππ* states, but with values in the range of 10 s^−1^ still rather small (Tables S5–S7).

For **SBF2‐CF3**, the states of nπ* character are energetically well above the low‐lying π_H_→π_L_ states. The first singlet state having CT character, S_3_ (SBF←Ph), is found >1 eV above S_1_ (Table S8) and is therefore not expected to play any role in the photoexcitation decay (or the corresponding triplet). As for **SBF2‐NO2**, S_1_→T_1_ is the major nonradiative decay channel of the S_1_ population (Table S9). Due to the energetic proximity of S_1_ and T_2_ (T_2 _≥ S_1_ by 20 meV), ISC S_1_→T_2_ is strongly thermally activated, but S_1_← T_2_ rISC is fast for **SBF2‐CF3** in DCM (Figure 3), as with **SBF2‐NO2**. However, T_2_ lies some >0.6 eV above T_1_ so again IC from T_2_ to T_1_ must compete strongly with any rISC (see Table S9).

In agreement with the computational results of Yu et al.,^[^ ^41^ ^]^ the lowest adiabatic excited singlet state of **CBF2‐NO2** is of mixed CT/LE character (Figure S12) in DCM and is dominated by the π_H_→π_L_ excitation (Table S10). Note, however, that the adiabatic order of singlet states is different in nonpolar environments such as Zeonex. In vacuum, the S_1_’ (n_NO2_→ π*_NB_) excitation is found slightly lower (*E*_adia_ = 2.45 eV) than the S_1_ (π_H_→π_L_) state (*E*_adia_ = 2.53 eV) (Table S11), although in both DCM and MCH, the measured PLQY is about the same at 0.71 and 0.77, respectively. In both environments, the T_1_ state has π_H_→π_L_ character and exhibits a large energy separation from the lowest singlet (∆*E*_ST_ ≈ 0.5 eV); thus, no TADF is expected.

In DCM, the vibrational density of states in the T_1_ potential of **CBF2‐NO2** at the energy of the S_1_ state is comparable to the one in **SBF2‐NO2**. However, due to the lack of a heavy atom (sulfur), the mutual S_1_–T_1_ SOCME is much smaller in **CBF2‐NO2** (0.133 cm^−1^) than in **SBF2‐NO2** (4.355 cm^−1^), resulting in an ISC rate constant that is reduced by 3–4 orders of magnitude (cp. Figure 3b,c as well as Tables S5 and S12). As the radiative rate constant of the S_1_ emission is nearly unchanged in comparison to **SBF2‐NO2**, the fluorescence quantum yield is greatly improved, as we find experimentally. The transition density of the T_1_ (π_H_→π_L_) excitation is mainly localized on the CBF2 unit with very small contributions from the NB part of the compound. Experimentally, we observe a well‐structured phosphorescence indicative of a predominant LE character. The calculated blue shift of the T_2_ (π_H‐1_→π_L_) state places its minimum energy about 0.25 eV above the S_1_ (π_H_→π_L_) minimum in DCM. Like **SBF2‐NO2**, the S_1_ (π_H_→π_L_)‐to‐T_2_ (π_H‐1_→π_L_) ISC is an uphill process in **CBF2‐NO2**, but the activation energy is much higher in the latter. Consequently, we compute a rate constant in the order of 10^5^ s^−1^ for this ISC process at room temperature, compared to a rISC rate constant of 4 × 10^8^ s^−1^. The calculations thus show that ISC and rISC are possible in this compound, but not very likely as they cannot compete against radiative decay of the S_1_ state (Figure 3). The deactivation of the optically dark S_1_’ (n_NO2_→ π*_NB_) state via the El‐Sayed‐allowed ISC to the T_1_ (π_H_→π_L_) state proceeds at similar rate constants in **SBF2‐NO2** and **CBF2‐NO2** as both transitions mainly involve n‐ and π‐type orbitals on the NB residue.

The S_2_ π_H‐1_→π_L_ singlet transition has a very large oscillator strength (radiative rate constant of 3 × 10^8^ s^−1^). Using an excitation wavelength of 355 nm, it could be possible to observe dual emission with the fast component from the S_2_ (π_H‐1_→π_L_) state and a slightly slower one from the S_1_ (π_H_→π_L_) state (radiative rate constant of 8 × 10^7^ s^−1^). The T_1_ π_H_→π_L_ triplet is the lowest triplet, and the S–T gap T_1_ (π_H_→π_L_)‐S_2_ (π_H_→π_L_) is 0.73 eV. Therefore, we do not expect TADF to occur in **CBF2‐NO2**. We also do not find a higher triplet state in energetic proximity to the S_1_ (π_H_→π_L_) minimum (Table S10).

### Time‐Resolved Photophysical Properties

**Figure 4:**
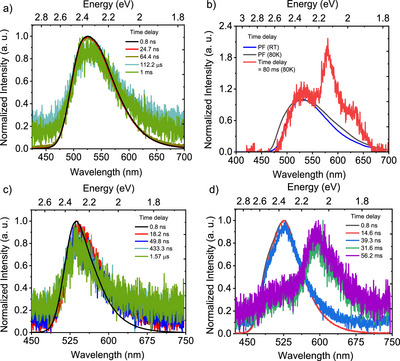
Optical properties of **CBF2‐NO2**. a) Time‐resolved spectra obtained at different delay times for 1 × 10^−5^ M **CBF2‐NO2** dissolved in dichloromethane at RT; b) prompt fluorescence at different temperatures along with phosphorescence at 80 K for 1 × 10^−5^ M **CBF2‐NO**2 dissolved in dichloromethane; time‐resolved spectra obtained at different delay times for **CBF2‐NO2**; c) in neat films and d) doped in Zeonex (1 wt.%) at RT **CBF2‐NO2**. *λ*_exc_ = 355 nm.

Time‐resolved emission measurements on this series of boron‐difluoride molecules were performed both in solution and solid states. Figures 4a,b and S16 show emission spectra of **CBF2‐NO2** in degassed dilute DCM ([*c*] = 1 × 10^−5^ M) at different time delays at 300 and 80 K, respectively. At 300 K, we observed a clear prompt fluorescence with a lifetime of 8 ns and a significantly weaker delayed fluorescence at the same onset energy (2.6 eV) as the PF having a lifetime of 291.4 µs (Figure 1c). This is in contradiction to the previously reported “only fluorescent” behavior of **CBF2‐NO2**.^[^ ^41^ ^]^

Given the very large singlet–triplet gap (Δ*E*_ST_ > 0.5 eV) and weak SOCME we calculate for this material, and believe that this DF emission is too weak to be observed in time correlated single‐photon counting (TCSPC) measurements^[^ ^41^ ^]^ and arises from triplet–triplet annihilation (TTA) in the dilute solution.^[^ ^62^ ^]^ Further, in MCH and Zeonex, we observe changes in the shape of the emission band at higher energy, which may indicate dual emission from the allowed S_1_(ππ*) and S_1_’ (n_NO2_→ π*_NB_) states consistent with our calculations (Figure S17). At 80 K, the **CBF2‐NO2** prompt fluorescence is slightly blue‐shifted (onset at 2.64 eV) as compared to room temperature (onset at 2.6 eV) (Figure 4b) consistent with the LE character of the S_1_ state. We also observe a red shoulder on S_1_ decay when **CBF2‐NO2** is dispersed in Zeonex (1 wt.%) at times of 35.1 ns, which is consistent with the weak but long‐lived S_1_’ nπ* again consistent with dual emission (Figure S24c).

At longer time delays, a strongly vibronically structured emission (*λ*_max_ = 2.14 eV, onset at 2.2 eV) is observed. This phosphorescence is indicative of an El‐Sayed allowed S_1_ (nπ*) → T_1_(ππ*) transition, as predicted from our calculations, and not a forbidden transition proposed by Yu et al.,^[^ ^41^ ^]^ giving a Δ*E*_ST_ of 450 meV, very close to that which we predict from calculation (Figure 4b). Additional weak, long‐lived delayed fluorescence at the same prompt emission onset (2.6 eV) is observed, again assumed to be very slow TTA given the magnitude of the Δ*E*_ST_ (Figure 4b).

**Figure 5:**
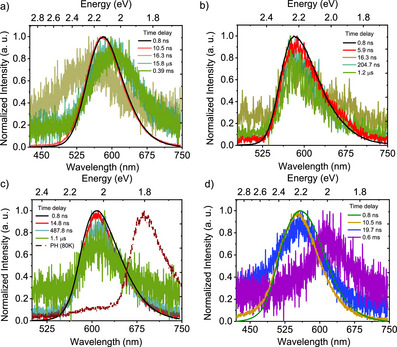
Optical properties of **SBF2‐NO2**. Time‐resolved spectra obtained at different delay times for a) 1 × 10^−5^ M and b) 16 × 10^−3^ M **SBF2‐NO2** dissolved in dichloromethane at RT. Time‐resolved spectra obtained at different delay times for **SBF2‐NO2** c) in neat films with the phosphorescence (PH) spectrum at 80 K is shown for **SBF2‐NO2** (time delay = 1 ms, integration time = 15 ms), and d) doped in Zeonex (1 wt.%) at RT **CBF2‐NO2**. *λ*_exc_ = 355 nm.

With a clear idea of the excited‐state characteristics of **CBF2‐NO2**, we investigated its sulfur‐containing congener **SBF2‐NO2** through time‐resolved emission spectroscopy. Figure 5a shows the time‐resolved decay of **SBF2‐NO2** in degassed and dilute ([*c*] = 1 × 10^−5^ M) DCM solvent. Prompt and delayed fluorescence were observed in the early (0.8–50 ns) and late (10–400 µs) time regions, respectively. A weak, blue‐shifted emission (450–500 nm) is observed when the time delay is over 10.5 ns, which grows in during prolonged irradiation (Figures 5a and S18). This is assigned to emission from photo‐degradation products of this derivative with the degradation confirmed by ^1^H NMR spectroscopy (Figures S18 and S19). Clear new peaks were observed for **SBF2‐NO2** in CD_2_Cl_2_ when it was irradiated with a hand‐held 365 nm UV lamp, compared with a nonirradiated sample (Figure S19). At 80 K, similar photodegradation was observed, but the emissive species became comparatively longer‐lived, indicative of slower nonradiative decay at low temperature (Figure S20). The fluorescence and phosphorescence at 80 K have several further interesting features in the case of **SBF2‐NO2** (Figure S20). The PF at 80 K, showed a blue‐shifted onset (2.5 eV) as compared to RT (2.35 eV), indicating a definite CT or mixed CT/LE character state. This is confirmed in our calculations with a predicted solvent shift of 0.05–0.1 eV for **SBF2‐NO2** at the S_1_ geometry (Tables S2 and S4). At 80 K, two distinct phosphorescence emissions were observed with different lifetimes and onsets (Figure S20). The shorter‐lived phosphorescence emission, onset at 2.17 eV (571 nm) in the time range of 1–10 ms matching that observed in **CBF2‐NO2**, in line with our calculations, and is assigned to the lowest excited triplet state of **SBF2‐NO2** (Figures 4b and S20; Table S5). However, a second phosphorescence band is observed at higher energy (onset at 2.45 eV, 506 nm) and at longer time delay (>15 ms), having significantly weaker intensity, and is ascribed to originate from the triplet states (phosphorescence) of the unknown photodegradation products noted above. This assignment is motivated by the fact that a longer‐lifetime phosphorescence emission from a higher energy triplet state (than the phosphorescence coming from the lowest triplet state) is physically impossible from the same molecule and is not consistent with any of our modeling results. This is not anti‐Kasha phosphorescence emission from decay of an upper triplet state to lowest triplet state as proposed by Feng et al.^[^ ^63^ ^]^

We further investigated **SBF2‐NO2** at 16 mM concentration in DCM, the concentration range where ASE was previously reported.^[^ ^44^ ^]^ Figure 5b shows RT time‐resolved spectra and time‐dependent decay is shown in Figure S21. Prompt fluorescence with a lifetime of 3 ns is followed by DF lasting over 1 µs, which is ascribed to strong TTA in this highly concentrated solution. A strong concentration dependence of the DF confirms it as TTA (Figures 5b and S21) and is fully consistent with an ∆*E*_ST_ being >280 meV and not TADF as proposed by Li et al.^[^ ^45^ ^]^ Interestingly, the high energy degradation peak observed at room temperature in dilute solutions is much less obvious, hinting that degradation occurs through the triplet state, which is quenched rapidly in high concentrated solution by the efficient TTA. Further, the high energy (degradation product) fluorescence peaks strongly overlap with the ground state absorption reducing this emission by reabsorption at high concentration. At 80 K, we observe a weak low‐energy phosphorescence at 2.17 eV (571 nm) matching that observed in the dilute conditions (Figures S20b and S21a). Additionally, a low‐energy feature grow‐in is seen in the µs time scale lasting beyond 0.99 ms (broad emission from 525 nm to 675 nm, peak at 600 nm at 12.4 ns) having a very different spectral shape to the longer‐lived phosphorescence. This, we believe, is the sign of aggregation, which is not surprising in such a highly concentrated frozen solution. This should also point to the high probability of aggregate contributions at RT when measurements are made in highly concentrated solutions (16 mM).

Comparing the emission characteristics at high concentration (16 mM), as shown in Figure 5b, to those at low concentration (Figure 5a), we observe a 100 meV red shift at high concentration, which further red‐shifts in the neat film state (Figures 5c and S22). This again indicates a high degree of aggregation in the high‐concentration solutions. As this concentration was used by Li et al. for ASE measurements,^[^ ^44^ ^]^ we believe it implies that the ASE in solution probably comes from the aggregated states and matches that which they observed in crystals.^[^ ^41^, ^42^ ^]^

To further probe the role of possible aggregate states in solution, neat solid films drop‐casted from DCM solutions were studied (Figures 1b, S1b, and S22). At room temperature, we observe prompt **CBF2‐NO2** emission at the same wavelengths as we observed for **SBF2‐NO2** in DCM solution, while **SBF2‐NO2** and **SBF2‐CF3** were even further redshifted. The emission band of **CBF2‐NO2** is sharper and more structured at 80 K compared to that of **SBF2‐NO2** (neat film) in line with their ππ* LE and mixed ππ* LE/CT characters from our theoretical results (Figures 4c, S22, and S23a). We observed a clear delayed fluorescence contribution at 80 K in both **CBF2‐NO2** (onset at 2.61 eV, 475 nm) and **SBF2‐NO2** (onset at 2.25 eV, 550 nm) consistent with the 16 mM **SBF2‐NO2** solutions, assigned to efficient TTA in line with their very large singlet–triplet energy gaps (Figure S23). At room temperature, **CBF2‐NO2** showed prompt fluorescence and delayed fluorescence features, having the same emission onset in their neat film state (Figure 4c). Although the prompt fluorescence decay could be fitted with a mono‐exponential decay (3 ns), the DF could not be fitted reliably as the emission intensity was very weak (Figure S23c). **SBF2‐NO2** showed a similar short‐lived prompt fluorescence component of lifetime 1–2 ns (quenched prompt) and slower components at 31.1 ns (unquenched prompt) and 218.1 ns (aggregate) (Figures 5c and S23b,d). In both of these derivatives, we do not observe any phosphorescence at room temperature in neat film, as reported previously.^[^ ^41^ ^]^ At 80 K, **CBF2‐NO2** showed no phosphorescence, although weak DF was still observed at this temperature with a long time delay (Figure S23a). On the other hand, a neat film of **SBF2‐NO2** at 80 K showed a distinct phosphorescence emission (onset at 1.92 eV, 646 nm, *λ*_max_ at 1.81 eV, 683 nm) as compared to the fluorescence (onset at 2.2 eV, 558 nm, *λ*_max_ at 2.04 eV, 608 nm) at RT (Figures 5c and S23d). Yu et al.,^[^ ^41^ ^]^ reported phosphorescence at 77 K at this latter energy, which we believe is in fact delayed emission from aggregates, not phosphorescence. Our observations contradict the previously proposed idea of amplified spontaneous emission from a (formally forbidden) T_1_→S_0_ transition in **SBF2‐NO2**.^[^ ^41^ ^]^

Comparing **SBF2‐NO2** in Zeonex (1 wt.%) with DCM, we see a 75 meV blue shift of the prompt emission indicative of polarity change in the environment and in excellent agreement with the calculated solvent shift of 50–100 meV for the S_1_ state of **SBF2‐NO2** (Figures 5a,d and S1). Clearly **SBF2‐NO2** has a CT S_1_ state and undergoes solvatochromic shift in DCM compared to Zeonex and neat film. Thus, when DCM solution is frozen, some **SBF2‐NO2** may precipitate to form aggregates, but also the frozen DCM solvent shell around each **SBF2‐NO2** molecule cannot reorientate to give the red solvent shift either, explaining the temperature‐dependent spectral shifts previously observed.

Comparing the emission spectra of 1 wt.%‐doped Zeonex films of **SBF2‐NO2** at room temperature and 80 K, we observe a 100 meV blue‐shift at 80 K (Figures 5d and S24a). However, the prompt emission band at 80 K exhibits a red shoulder that decays rapidly (within 6.2 ns), which we tentatively attribute to the presence of the S’ nπ* state (Figure S24a). This state emits but is quickly quenched by ISC, as indicated by our calculations. This observation shows the strong decoupling of the S and S’ excited states. In DCM, the S’ is blue‐shifted to slightly higher energies than the S_1_ ππ* state and could be the cause of the rather weak blue shoulder on the prompt emission observed in DCM (Figure 5a). Figure S24a shows clear well‐structured 0.707 ms phosphorescence at 2.06 eV (602 nm) and matches perfectly with the **SBF2‐NO2** triplet energy, indicating that the triplet state is localized on the SBF2 unit (Figure S25). In **CBF2‐NO2** with no S in the “donor” unit, the triplet is blue shifted to 2.16 eV, in excellent agreement with the theory. Further, we do not see any delayed fluorescence (both at RT and 80 K) in dilute polymer matrix, which confirms that in these emitters any delayed emission observed, either in solution state or in neat films, has to be TTA, not TADF.^[^ ^64^ ^]^ At 80 K, we again observed two phosphorescence bands in **SBF2‐NO2** (Figure S25a), consistent with our observations in low‐temperature solution‐state experiments (vide infra). This observation suggests rapid photodegradation of the **SBF2‐NO2**‐doped Zeonex films leading to the high‐energy phosphorescence emission at low temperature.

Unfortunately, the delayed fluorescence intensity in both these derivatives is too weak in solution or neat films for any reliable intensity‐dependent measurement to be made to unambiguously assign bimolecular (TTA) or unimolecular (TADF or upper‐triplet crossing) processes. However, this issue was addressed with the structurally similar **SBF2‐CF3** derivative, detailed in the next section.

**Figure 6:**
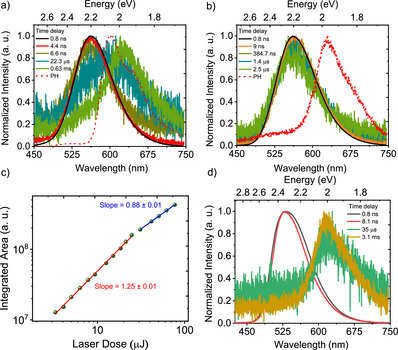
Optical properties of **SBF2‐CF3**. Time‐resolved spectra obtained at different delay times for a) 1 × 10^−5^ M **SBF2‐CF3** dissolved in dichloromethane and b) neat **SBF2‐CF3** films at RT and 80 K, *λ*_exc_ = 355 nm; c) Integrated area as a function of the laser excitation (*λ*_exc_ = 337 nm) of neat **SBF2‐CF3** films at RT (time delay = 0.7 µs and integration time = 5 µs). d) Time‐resolved spectra obtained at different delay times for **SBF2‐CF3** doped in Zeonex (1 wt.%) at RT. *λ*_exc_ = 355 nm, PH = phosphorescence (measured at 80 K, at delay time = 1 ms, integration time = 15 ms).

Figure 6a shows time‐resolved decay from **SBF2‐CF3** in dilute DCM (1 × 10^−5^ M) solution. We clearly see a combination of PF until ∼10 ns, followed by DF and RTP emission between 22.2 µs and 0.63 ms. The prominent RTP emission in **SBF2‐CF3** is due to its large singlet–triplet gap (>200 meV), therefore giving an expected inefficient reverse intersystem crossing. At 80 K, we again observe similar strong degradation‐induced prompt fluorescence and corresponding phosphorescence features in line with that observed in **SBF2‐NO2** (Figures S18c, S19c, and S26a,b). In the neat film state, **SBF2‐CF3** shows a significantly stronger DF (0.9 µs), along with a prompt fluorescence at 2.45 eV (490 nm) (Figures 6b and S26a). In this case, the power‐dependent delayed fluorescence intensity was measured to elucidate its mechanism (Figure 6c). The integrated DF emission intensity, as a function of the laser excitation (337 nm) dose was collected over the delayed fluorescence region (time delay = 0.7 µs and integration time = 5 µs). The intensity dependence shows a slope of 1.25 ± 0.01 at low excitation dose (<11 µJ), which turns to a (sub)linear dependence with slope of 0.88 ± 0.01 at high excitation doses. This suggests that the delayed fluorescence observed for **SBF2‐CF3** neat films has a contribution from both TTA and TADF. The behavior at low excitation doses is expected to show a slope closer to 2 for a pure TTA‐delayed fluorescence.^[^ ^65^ ^]^ Therefore, the power‐dependent studies of the delayed fluorescence may indicate that there could be contribution from TADF as well as TTA. We highlight, though, that there is significant singlet quenching at high excitation doses (slope below 1). To understand these rather complex decay dynamics and the effectiveness of bimolecular TTA in neat films, we studied the photophysics of **SBF2‐CF3** in dilute 1 wt.% Zeonex films. In line with our observations for **SBF2‐NO2** and **CBF2‐NO2** doped in Zeonex films, we observed a complete disappearance of the delayed fluorescence band and a concomitant appearance of the room temperature phosphorescence (onset 566 nm, 2.19 eV) at high time delays (Figures 6d and S26). This clearly verifies that the DF in **SBF2‐CF3** has a bimolecular nature consistent with TTA, not unimolecular TADF. We therefore assume that other bimolecular quenching processes are occurring in **SBF2‐CF3**, potentially singlet–triplet quenching or triplet quenching at degradation sites.

### Dimer Formation in Concentrated Solutions

**Figure 7:**
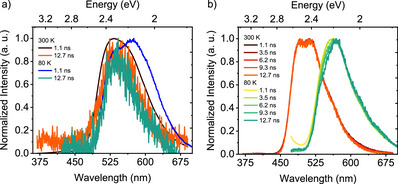
Optical properties of **SBF2‐NO2** and **CBF2‐NO2** in methylcyclohexane. Time‐resolved spectra obtained at different delay times for 1 × 10^−4^ M a) **SBF2‐NO2** and b) **CBF2‐NO2** dissolved in methylcyclohexane at RT and 80 K. *λ*_exc_ = 355 nm.

Last, we investigated further the aggregation phenomena in these materials to gain more insight into the previous studies performed at high concentrations and in crystals.^[^ ^41^, ^44^, ^45^ ^]^ **SBF2‐NO2** at 100 µM in MCH shows no indication of aggregation at room temperature (Figure S27a), only a ca. 60 nm solvent‐induced blue shift compared to emission in DCM. The observed prompt emission decays with a single exponential lifetime of 2.98 ns (Figure S28). On cooling the solution to 80 K, we observe dual prompt emission, consistent with monomer and weak aggregate (potentially dimer), the dimer being 35 nm redder than the monomer (Figures 7a and S29). The dimer decays more quickly than the monomer (Figure 7a). At long delay times at 80 K, we observe dual phosphorescence; in the first few milliseconds, we see structured phosphorescence, onset at 565 nm, at much longer delay times (>40 ms), a second phosphorescence band is seen (onset at 470 nm), consistent with the emission from the previously identified degradation product (vide infra, Figure S29).

**Figure 8:**
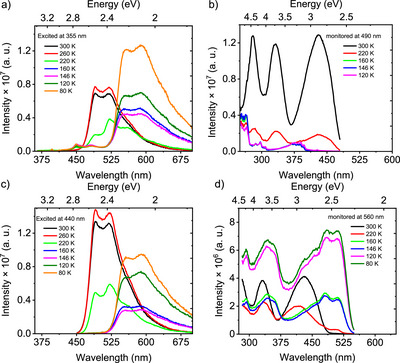
Temperature‐dependent (absolute) emission properties of **CBF2‐NO2** in methylcyclohexane. Steady‐state emission spectra excited at a) 355 nm, c) 440 nm and excitation spectra monitored at b) 490 nm, and d) 560 nm of 10 × 10^−5^ M **CBF2‐NO2** dissolved in methylcyclohexane at different temperatures.

In contrast, **CBF2‐NO2** behaves very differently under similar experimental conditions (in 100 µM MCH) (Figures 7b and S27b). As in **SBF2‐NO2**, at room temperature little evidence of aggregation is seen; however, as the solution is cooled to below 220 K, the onset of strong aggregation is observed and appears to be fully aggregated by 160 K (Figures 7b and 8a,c). This we ascribe to the head‐to‐tail molecular packing in **CBF2‐NO2** compared to head‐to‐head packing in **SBF2‐NO2** (Figures S2 and S3).

In both the excitation and emission profiles of **CBF2‐NO2**, strikingly large differences are observed (Figure 8). A new emission band grows in red‐shifted by 60 nm (onset at 525 nm), while the monomer emission band (onset at 450 nm) rapidly disappears (from 260 K to 160 K). A new absorption spectrum, dominated by a structured red absorption at 525 nm (red‐shifted by 60 nm), grows in over the same temperature range. In both cases, the aggregate emission and absorption increase in intensity as the temperature further drops to 80 K, potentially indicating further evolution of the aggregate states. In addition, measuring the excitation spectra at the peak of the monomer emission (490 nm), a total loss of the monomer absorption band is seen, leaving an underlying weak but highly structured absorption onset at 400 and 315 nm (Figure 8b). We believe these to be from the weak nπ* S_1_’ state. Exciting at 355 nm gives 50% less monomer emission than exciting at 440 nm but ca. 25% more aggregate emission (Figure S30). Understood as excitation at 355 nm excites both the S_1_ and S_2_ transitions giving rise to a weak but structured emission (onset at 435 nm) from the S_2_ states, visible after aggregation (Figure 8a). The monotonic increase in aggregate intensity as the temperature decreases may indicate changes in the transition dipole moments and oscillator strengths of transitions in the aggregates. Phosphorescence (single band) is observed at 80 K in the millisecond time window, with onset at 550 nm, the same onset as observed in dilute DCM solutions, showing no obvious signs of aggregation (Figures 4b and S28d). This may point to a highly localized triplet state on individual molecules within the aggregate. As such, this is the first clear evidence about the nature of triplet states in dimers/aggregates. This phenomenon will be further studied in future work.

Next, the optical properties of **SBF2‐NO2** and **CBF2‐NO2** crystals grown from DCM solutions were measured (Figure S31). In **SBF2‐NO2**, the excitation spectrum begins at an onset of 590 nm (monitored at 640 nm), while a Gaussian‐shaped emission band is seen at 560 nm (*λ*_max_ = 610 nm) (Figure S31a,b). This long prompt emission decays biexponentially (3.63 and 55.07 ns) but without spectral (energy) relaxation,^[^ ^66^ ^]^ indicative of prompt and delayed emission via TTA (Figures S31c and S32). In **CBF2‐NO2**, excitation onset is at 520 nm (*λ*_monitored_ = 560 nm) and emission onset at 490 nm (*λ*_max_ = 540 nm) (Figure S31a,b). The emission band has a main peak at 540 nm and a very weak band at 400–450 nm. Measuring the time‐dependent emission, we find that these are two separate emission decays, the 540 nm band decays much faster with far greater intensity than the 400–450 nm band (Figure S31d). This blue feature may indicate excitonic splitting due to the crystal stacking. However, it is clear that, in **CBF2‐NO2**, the excited states in the dimer/aggregate are different from those in the crystal, with the aggregate being more redshifted (*λ*_max_ = 600 nm) than the crystal states (*λ*_max_ = 540 nm) (Figures 8a and S31b).

**Figure 9:**
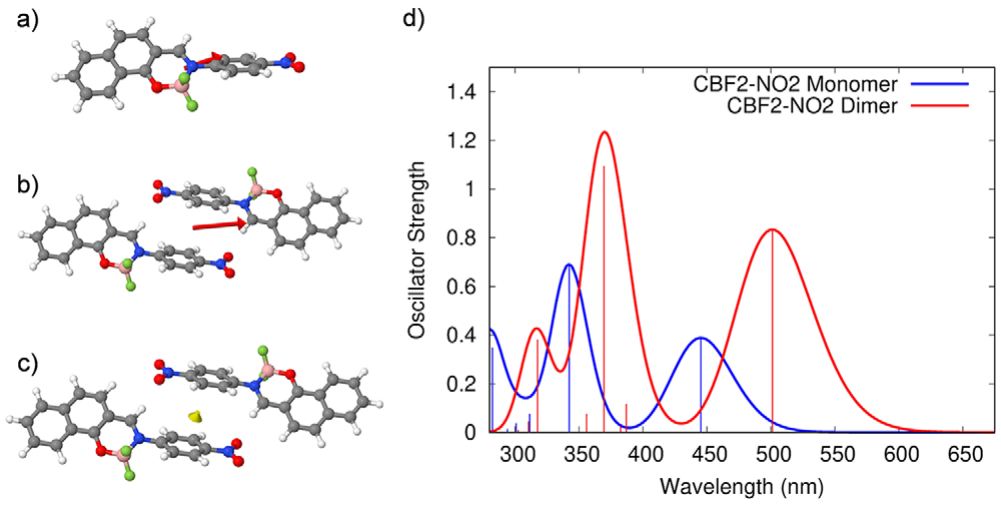
Calculated a) monomer and b) dimer transition dipole moment (red arrow), and c) dimer static dipole moment (yellow arrow) of **CBF2‐NO2** in vacuum. The doubling of the transition dipole moment in the dimer instead of cancelation is ascribed to an excitonic coupling effect. d) Calculated dimer versus monomer spectrum in vacuum.

This behavior was modeled using a dimer structure in vacuum (Figures 9 and S33). It was found that a dimer configuration taken from the crystal structure was not the global minimum energy configuration of a dimer in nonpolar solvent medium, which showed a strong slipping of the molecules such that only the neighboring nitrobenzene units co‐facially overlapped (Figure 9b), compared to the whole molecules overlapping in the crystal (Figure S2). From the minimum energy dimer configuration and the monomer, absorption spectra were calculated and found to be in excellent agreement with the experimentally measured spectra (Figures 8d and 9d). The lowest energy configuration of the dimer in nonpolar solvent is shown in Figure 9b. The static dipole moment between the molecules is negligible, indicating π‐stacking to be the dominant packing force (Figure 9c). The transition dipole moment (TDM) in the dimer is, however, found to be twice that of the monomer even though the head‐to‐tail structure should cancel the monomer TDM; this data indicates that excitonic interactions are strong in these dimers. These results also show that molecular stacking within small aggregates in solution is different from that in crystals and the solvent plays an important role in modifying the π–π stacking forces. This leads to different optical properties and photophysics in solvated π‐stacks, characteristic of J‐aggregates.^[^ ^67^, ^68^ ^]^ This potentially is the reason why ASE in solution from these aggregates can be observed.

## Conclusion

In summary, we have synthesized a series of three molecules based on a donor–acceptor boron difluoride family that were previously reported as triplet emitters, of either room temperature phosphorescence (RTP) or delayed fluorescence in origin that could give rise to amplified stimulated emission (ASE) from either stimulated phosphorescence or TADF.^[^ ^40^, ^41^, ^42^, ^43^, ^44^, ^45^, ^46^ ^]^ An important general conclusion of the present study is that it is essential to apply high‐level photophysics and theory before defining the emission processes. Detailed quantum chemistry calculations at a high level of theory (DFT/MCRI) combined with a wide range of time‐resolved spectroscopy measurements have now elucidated the complex photophysical properties of these materials. The control molecule, **CBF2‐NO2**, emits prompt and delayed fluorescence without having a sulfur atom present, questioning sulfur's role as a heavy atom in triplet‐emitting systems.^[^ ^14^ ^]^ On the other hand, both **SBF2‐NO2** and **SBF2‐CF3** (which contain a sulfur atom) emit via prompt and delayed fluorescence in neat films, but no RTP is observed, only a very minor contribution of RTP in degassed solution state is seen. The majority of the DF component was clearly shown to come from TTA, from concentration‐dependent measurements and experiments with the emitters diluted in an inert Zeonex host. Further, ubiquitous photodegradation is identified, especially in **SBF2‐NO2** and **SBF2‐CF3**, resulting in new high‐energy prompt emission and phosphorescence emission bands with their own lifetimes. In previous studies, these bands were erroneously assigned to phosphorescence from higher excited triplet states (above T_1_).^[^ ^40^, ^41^, ^42^, ^43^, ^44^, ^45^, ^46^ ^]^ In all three materials, we see a strong tendency for aggregation in DCM solution, confirmed by neat film measurements. In nonpolar MCH, there is extreme aggregation upon cooling **CBF2‐NO2** solutions, but much weaker aggregation with **SBF2‐NO2**, which is ascribed to the different head‐to‐tail and head‐to‐head molecular packing, confirmed in calculations. Moreover, comparing the theoretical minimum energy dimer configuration of **CBF2‐NO2** to its configuration in crystal, we find large differences with the solution dimer state yielding a lower energy excited state than in the crystal. Also, in solution there is weak π–π stacking with strong excitonic effects giving rise to a doubling of the oscillator strength, indicating a strong perturbation of the molecular interaction coming from the solvent and giving rise to strong excitonic coupling between the slip‐stacked head‐to‐tail molecules in this packing conformation. In the aggregated state, we also observe weak emission from the nπ* S_1_’ state. This shows how strongly the solvent affects molecular π stacking, but also that triplet states remain rather localized to individual molecules. Our results unravel a general emission pathway that was previously reported as phosphorescent ASE to be, instead, from aggregate singlet states in concentrated solution, matching the crystal state ASE properties previously reported, not from monomeric delayed fluorescence or phosphorescence. Both our modeling and photophysical measurements clearly rule out the latter mechanisms. Moreover, significant triplet–triplet annihilation contributions to DF have now been identified in this class of materials, which have comparatively large singlet–triplet energy gaps. Our measurements show that using even relatively low concentrations to obtain solution‐state photophysics with molecules that readily interact needs great care and a lot of attention needs to be paid to differentiate true monomolecular properties from those of ill‐defined aggregates. Therefore, the design of donor–acceptor derivatives with smaller Δ*E*_ST_ is the way forward to avoid TTA losses and to boost triplet harvesting for future development of this class of molecules as organic laser gain materials in crystal form.

## Supporting Information

The authors have cited additional references within the Supporting Information.^[^ ^41^, ^44^, ^45^ ^,^ ^69^, ^70^, ^71^, ^72^, ^73^, ^74^, ^75^ ^]^ Synthetic details, NMR and mass spectra, additional absorption and emission spectra, and computational data are also included. The CIF files for **CBF2‐NO2** and **SBF2‐NO2** have been deposited with the Cambridge Structural Database, CCDC 2434826 and 2434827, respectively.

## Conflict of Interests

The authors declare no conflict of interest.

## Supporting information



Supporting Information

Supporting Information

## Data Availability

The data that support the findings of this study are available in the Supporting Information of this article.

## References

[1] H. Uoyama , K. Goushi , K. Shizu , H. Nomura , C. Adachi , Nature 2012, 492, 234–238. 23235877 10.1038/nature11687

[2] B. T. Luppi , W. L. Primrose , Z. M. Hudson , Angew. Chem. Int. Ed. 2024, 63, e202400712. 10.1002/anie.202400712 38439710

[3] C. J. Christopherson , N. R. Paisley , Z. Xiao , W. R. Algar , Z. M. Hudson , J. Am. Chem. Soc. 2021, 143, 13342–13349. 34382775 10.1021/jacs.1c06290

[4] M. A. Baldo , M. E. Thompson , S. R. Forrest , Nature 2000, 403, 750–753. 10693799 10.1038/35001541

[5] M. A. Baldo , D. F. O'Brien , M. E. Thompson , S. R. Forrest , Phys. Rev. B 1999, 60, 14422–14428.

[6] B. D. Ravetz , A. B. Pun , E. M. Churchill , D. N. Congreve , T. Rovis , L. M. Campos , Nature 2019, 565, 343–346. 30651612 10.1038/s41586-018-0835-2 PMC6338432

[7] J. Xia , S. N. Sanders , W. Cheng , J. Z. Low , J. Liu , L. M. Campos , T. Sun , Adv. Mater. 2017, 29, 1601652. 10.1002/adma.201601652 27973702

[8] A. J. Gillett , A. Privitera , R. Dilmurat , B. D. Naidu , X. Q. Bai , A. K. Y. Jen , L. M. Herz , H. Sirringhaus , R. H. Friend , Nature 2021, 597, 666–671.34588666

[9] M. A. El‐Sayed , Acc. Chem. Res. 1968, 1, 8–16.

[10] S. Kuila , H. Miranda‐Salinas , J. Eng , C. Li , M. R. Bryce , T. J. Penfold , A. P. Monkman , Nat. Commun. 2024, 15, 9611. 39511188 10.1038/s41467-024-53740-1 PMC11544105

[11] C. J. Chiang , A. Kimyonok , M. K. Etherington , G. C. Griffiths , V. Jankus , F. Turksoy , A. P. Monkman , Adv. Funct. Mater. 2013, 23, 739–746.

[12] R. Ieuji , K. Goushi , C. Adachi , Nat. Commun. 2019, 10, 5283. 31754203 10.1038/s41467-019-13044-1 PMC6872538

[13] F. B. Dias , K. N. Bourdakos , V. Jankus , K. C. Moss , K. T. Kamtekar , V. Bhalla , J. Santos , M. R. Bryce , A. P. Monkman , Adv. Sci. 2016, 3, 1600080. 10.1002/adma.201300753 23703877

[14] S. Öner , S. Kuila , K. Stavrou , A. Danos , M. A. Fox , A. P. Monkman , M. R. Bryce , Chem. Mater. 2024, 36, 7135–7150. 39156711 10.1021/acs.chemmater.4c00850 PMC11325549

[15] P. L. dos Santos , F. B. Dias , A. P. Monkman , J. Phys. Chem. C 2016, 120, 18259–18267.

[16] K. Stavrou , L. G. Franca , A. Danos , A. P. Monkman , Nat. Photonics 2024, 18, 554–561.

[17] C.‐C. Yan , X.‐D. Wang , L.‐S. Liao , Adv. Sci. 2022, 9, 2200525. 10.1002/advs.202200525 PMC9165517 35344285

[18] M. C. Gather , S. H. Yun , Nat. Commun. 2014, 5, 5722. 25483850 10.1038/ncomms6722 PMC4385288

[19] Z. Zhou , C. Qiao , K. Wang , L. Wang , J. Liang , Q. Peng , Z. Wei , H. Dong , C. Zhang , Z. Shuai , Y. Yan , Y. S. Zhao , Angew. Chem. Int. Ed. 2020, 59, 21677–21682. 10.1002/anie.202008940 32789916

[20] C. A. M. Senevirathne , S. Yoshida , M. Auffray , M. Yahiro , B. S. B. Karunathilaka , F. Bencheikh , K. Goushi , A. S. D. Sandanayaka , T. Matsushima , Adv. Opt. Mater. 2022, 10, 2101302.

[21] G. Sardar , A. Shukla , E. G. Moore , G. Banappanavar , S.‐C. Lo , E. B. Namdas , D. Kabra , J. Phys. Chem. C 2022, 126, 9069–9075.

[22] Y. Jiang , P. Lv , J.‐Q. Pan , Y. Li , H. Lin , X.‐W. Zhang , J. Wang , Y.‐Y. Liu , Q. Wei , G.‐C. Xing , W.‐Y. Lai , W. Huang , Adv. Funct. Mater. 2019, 29, 1806719.

[23] M. A. Baldo , R. J. Holmes , S. R. Forrest , Phys. Rev. B 2002, 66, 035321.

[24] A. S. D. Sandanayaka , K. Yoshida , M. Inoue , K. Goushi , J.‐C. Ribierre , T. Matsushima , C. Adachi , Adv. Opt. Mater. 2016, 4, 834–839. 10.1126/sciadv.1602570 PMC5409494 28508042

[25] P. Lova , V. Grande , G. Manfredi , M. Patrini , S. Herbst , F. Würthner , D. Comoretto , Adv. Opt. Mater. 2017, 5, 1700523.

[26] D.‐H. Kim , A. D'Aléo , X.‐K. Chen , A. S. D. Sandanayaka , D. Yao , L. Zhao , T. Komino , E. Zaborova , G. Canard , Y. Tsuchiya , E. Choi , J. W. Wu , F. Fages , J.‐L. Brédas , J.‐C. Ribierre , C. Adachi , Nat. Photonics 2018, 12, 98–104.

[27] T. Zhang , Z. Zhou , X. Liu , K. Wang , Y. Fan , C. Zhang , J. Yao , Y. S. Zhao , J. Am. Chem. Soc. 2021, 143, 20249–20255. 34797057 10.1021/jacs.1c08824

[28] J.‐J. Wu , X.‐D. Wang , L.‐S. Liao , Laser Photonics Rev. 2022, 16, 2200366.

[29] A. J. C. Kuehne , M. C. Gather , Chem. Rev. 2016, 116, 12823–12864. 27501192 10.1021/acs.chemrev.6b00172

[30] B. K. Yap , R. Xia , M. Campoy‐Quiles , P. N. Stavrinou , D. D. C. Bradley , Nat. Mater. 2008, 7, 376–380. 18408724 10.1038/nmat2165

[31] U. Scherf , S. Riechel , U. Lemmer , R. F. Mahrt , Curr. Opin. Solid State Mater. Sci. 2001, 5, 143–154.

[32] C. Rothe , F. Galbrecht , U. Scherf , A. Monkman , Adv. Mater. 2006, 18, 2137–2140.

[33] M. Cherpak , E. Angioni , N. J. Findlay , B. Breig , V. Cherpak , P. Stakhira , J. V. Grazulevicius , Y. A. Nastishin , O. D. Lavrentovich , P. J. Skabara , ACS Appl. Mater. Interfaces 2017, 9, 4750–4757. 28078885 10.1021/acsami.6b13689

[34] K. Yoshida , J. Gong , A. L. Kanibolotsky , P. J. Skabara , G. A. Turnbull , I. D. W. Samuel , Nature 2023, 621, 746–752. 37758890 10.1038/s41586-023-06488-5 PMC10533406

[35] M. D. McGehee , A. J. Heeger , Adv. Mater. 2000, 12, 1655–1668.

[36] A. J. Das , C. Lafargue , M. Lebental , J. Zyss , K. Narayan , Appl. Phys. Lett. 2011, 99, 263303.

[37] A. J. C. Kuehne , D. Eljström , A. R. Mackintosh , A. Kanibolotsky , B. Guilhabert , E. Gu , I. F. Perepichka , P. J. Skabara , M. D. Dawson , R. A. Pethrick , Adv. Mater. 2009, 21, 781–785.

[38] M. Mamada , S. Maedera , S. Oda , T. B. Nguyen , H. Nakanotani , T. Hatakeyama , C. Adachi , Mater. Chem. Front. 2023, 7, 259–266.

[39] X. Tang , M. Xie , Z. Lin , K. Mitrofanov , T. Tsagaantsooj , Y.‐T. Lee , R. Kabe , A. S. D. Sandanayaka , T. Matsushima , T. Hatakeyama , C. Adachi , Angew. Chem. Int. Ed. 2023, 63, e202307531. 10.1002/anie.202315210 37991245

[40] X. Wang , Z. Z. Li , S. F. Li , H. Li , J. Chen , Y. Wu , H. Fu , Adv. Opt. Mater. 2017, 5, 1700027.

[41] Z. Yu , Y. Wu , L. Xiao , J. Chen , Q. Liao , J. Yao , H. Fu , J. Am. Chem. Soc. 2017, 139, 6376–6381. 28414231 10.1021/jacs.7b01574

[42] H. Huang , Z. Yu , D. Zhou , S. Li , L. Fu , Y. Wu , C. Gu , Q. Liao , H. Fu , ACS Photonics 2019, 6, 3208–3214.

[43] F. Yin , J. De , M. Liu , H. Huang , H. Geng , J. Yao , Q. Liao , H. Fu , Nano Lett. 2022, 22, 5803–5809. 35848711 10.1021/acs.nanolett.2c01345

[44] S. Li , Z. Yu , X. Xiao , H. Geng , K. Wang , X. Jin , Q. Liao , Y. Liao , Y. Wu , J. Yao , H. Fu , Laser Photonics Rev. 2019, 13, 1900036.

[45] S. Li , X. Jin , Z. Yu , X. Xiao , H. Geng , Q. Liao , Y. Liao , Y. Wu , W. Hu , H. Fu , J. Mater. Chem. C 2021, 9, 7400–7406.

[46] S. Li , J. Chen , Y. Wei , J. De , H. Geng , Q. Liao , R. Chen , H. Fu , Angew. Chem. Int. Ed. 2022, 61, e202209211. 10.1002/anie.202209211 35923091

[47] J. P. Perdew , K. Burke , M. Ernzerhof , Phys. Rev. Lett. 1996, 77, 3865–3868. 10062328 10.1103/PhysRevLett.77.3865

[48] C. Adamo , V. Barone , J. Chem. Phys. 1999, 110, 6158–6170.

[49] A. Schäfer , H. Horn , R. Ahlrichs , J. Chem. Phys. 1992, 97, 2571–2577.

[50] M. J. Frisch , G. W. Trucks , H. B. Schlegel , G. E. Scuseria , M. A. Robb , J. R. Cheeseman , G. Scalmani , V. Barone , G. A. Petersson , H. Nakatsuji , X. Li , M. Caricato , A. V. Marenich , J. Bloino , B. G. Janesko , R. Gomperts , B. Mennucci , H. P. Hratchian , J. V. Ortiz , A. F. Izmaylov , J. L. Sonnenberg , D. Williams‐Young , F. Ding , F. Lipparini , F. Egidi , J. Goings , B. Peng , A. Petrone , T. Henderson , D. Ranasinghe , et al., Gaussian 16, revision A.03, Gaussian Inc., Wallingford, CT.

[51] I. Lyskov , M. Kleinschmidt , C. M. Marian , J. Chem. Phys. 2016, 144, 034104. 26801017 10.1063/1.4940036

[52] C. M. Marian , A. Heil , M. Kleinschmidt , WIREs Comput. Mol. Sci. 2019, 9, e1394.

[53] C. Lee , W. Yang , R. G. Parr , Phys. Rev. B 1988, 37, 785–789. 10.1103/physrevb.37.785 9944570

[54] A. D. Becke , J. Chem. Phys. 1993, 98, 1372–1377.

[55] E. Cancès , B. Mennucci , J. Tomasi , J. Chem. Phys. 1997, 107, 3032.

[56] B. Mennucci , E. Cancès , J. Tomasi , J. Phys. Chem. B 1997, 101, 10506–10517.

[57] TURBOMOLE 7.5, 2020, TURBOMOLE GmbH, available from http://www.turbomole.com.

[58] M. Kleinschmidt , J. Tatchen , C. M. Marian , J. Comput. Chem. 2002, 23, 824–833. 12012359 10.1002/jcc.10064

[59] M. Kleinschmidt , J. Tatchen , C. M. Marian , J. Chem. Phys. 2006, 124, 124101. 16599656 10.1063/1.2173246

[60] M. Etinski , J. Tatchen , C. M. Marian , Phys. Chem. Chem. Phys. 2014, 16, 4740. 24469462 10.1039/c3cp53247j

[61] M. Etinski , V. Rai‐Constapel , C. M. Marian , J. Chem. Phys. 2014, 140, 114104. 24655169 10.1063/1.4868484

[62] S. Paredis , T. Cardeynaels , S. Kuila , J. Deckers , M. Van Landeghem , K. Vandewal , A. Danos , A. P. Monkman , B. Champagne , W. Maes , Chem. Eur. J. 2023, 29, e202301369. 37154211 10.1002/chem.202301369

[63] C. Feng , S. Li , L. Fu , X. Xiao , Z. Xu , Q. Liao , Y. Wu , J. Yao , H. Fu , J. Phys. Chem. Lett. 2020, 11, 8246–8251. 32915577 10.1021/acs.jpclett.0c02180

[64] Y. Li , K. Wang , Q. Liao , L. Fu , C. Gu , Z. Yu , H. Fu , Nano Lett. 2021, 21, 3287–3294. 33724847 10.1021/acs.nanolett.1c00632

[65] V. Jankus , C.‐J. Chiang , F. Dias , A. P. Monkman , Adv. Mater. 2013, 25, 1455–1459. 23281058 10.1002/adma.201203615

[66] P. L. dos Santos , M. K. Etherington , A. P. Monkman , J. Mater. Chem. C 2018, 6, 4842–4853.

[67] N. J. Hestand , F. C. Spano , Chem. Rev. 2018, 118, 7069–7163. 29664617 10.1021/acs.chemrev.7b00581

[68] Z. Chen , A. Lohr , C. R. Saha‐Möller , F. Würthner , Chem. Soc. Rev. 2009, 38, 564–584. 19169466 10.1039/b809359h

[69] G. R. Fulmer , A. J. M. Miller , N. H. Sherden , H. E. Gottlieb , A. Nudelman , B. M. Stoltz , J. E. Bercaw , K. I. Goldberg , Organometallics, 2010, 29, 2176–2179.

[70] APEX5, v.2023.9‐2, Bruker AXS Inc., Madison, WI, USA, 2019.

[71] SAINT+ v8.40.0, Bruker AXS Inc., Madison, WI, USA, 2019.

[72] SADABS, Bruker AXS Inc., Madison, WI, USA, 2019.

[73] G. M. Sheldrick , Acta Crystallogr. A 2015, 71, 3–8. 10.1107/S2053273314026370 PMC4283466 25537383

[74] G. M. Sheldrick , Acta Crystallogr. C 2015, 71, 3–8. 10.1107/S2053273314026370 PMC4283466 25537383

[75] O. V. Dolomanov , L. J. Bourhis , R. J. Gildea , J. A. K. Howard , H. Puschmann , J. Appl. Crystallogr. 2009, 42, 339–341. 10.1107/S0021889811041161 PMC3236671 22199401

