# Supplementary material for: Unraveling the Role of Triplet–Triplet Annihilation and Photodegradation in Difluoroboron‐Based Organic Laser Gain Materials
Source: Angew Chem Int Ed Engl. 2025 Sep 16;64(45):e202509535. doi: 10.1002/anie.202509535 (PMC12582015; doi:10.1002/anie.202509535)
Supplement: Supplementary file 1 — Supporting Information [file ANIE-64-e202509535-s002.pdf]

Unravelling the Role of Triplet-triplet Annihilation and Photodegradation in Difluoroboron-based Organic Laser Gain Materials

Suman Kuila,[a,b] Hector Miranda-Salinas,[a] Chunyong Li,[a] Natalie E. Pridmore,[b] Martin R. Bryce,[b] Christel M. Marian,[c] and Andrew P. Monkman[a]*

[a] Dr. S. Kuila, H. Miranda-Salinas, Dr. C. Li, Prof. Dr. A. P. Monkman

Department of Physics, Durham University, South Road, Durham, DH1 3LE, UK

E-mail: a.p.monkman@durham.ac.uk

[b] Dr. S. Kuila, Dr. N. E. Pridmore, Prof. Dr. M. R. Bryce

Department of Chemistry, Durham University, South Road, Durham, DH1 3LE, UK

[c] Prof. Dr. C. M. Marian

Institute of Theoretical and Computational Chemistry, Faculty of Mathematics and Natural Sciences, Heinrich Heine University Düsseldorf, D-40204 Düsseldorf, Germany

Table of Content

1. General Experimental Details – Methods and Instruments....2

2. Sample Preparation for Optical Measurements....2

3. Optical Properties, Structural Characterizations and Theoretical Modelling....3

4. Synthetic Scheme and Characterization of the Compounds....32

5. References....45

1. General Experimental Details – Methods and Instruments.

Commercial reagents were purchased and used without further purification. Reactions were conducted under an argon atmosphere, unless otherwise stated. Glassware was dried overnight in an oven at 80 °C. Solvents and liquid reagents were added by syringe or cannula, and solid reagents were added under a positive pressure of argon. Degassing was performed by bubbling argon through the reaction mixture using an argon-filled balloon fitted with a syringe needle. Thin layer chromatography (TLC) analysis was performed by using Merck Silica gel 60 F254 TLC plates and spots were visualized by UV irradiation at 365 and 254 nm. Column chromatography was performed using silica gel 60 purchased from Fluorochem. NMR spectroscopy was carried out on Bruker AV400 spectrometers. Spectra were recorded at 295 K in commercially available deuterated solvents and referenced internally to the residual solvent proton resonances.[69] Atmospheric pressure solids analysis probe (ASAP) ionization mass spectra were obtained using an LCT Premier XE mass spectrometer and an Acquity® UPLC from Waters Ltd at 350°C. High-resolution mass spectrometry was carried out on a Quantum time-of-flight (QToF) mass spectrometer. The X-ray single crystal data have been collected at a temperature of 120.0(2) K using MoKα radiation (λ =0.71073Å) on a Bruker D8 Venture with a Photon III MM C7 or C14 CPAD detector respectively, IμS-III-microsource, focusing mirrors diffractometer equipped with a Cryostream (Oxford Cryosystems 700+) open-flow nitrogen cryostat. Using APEX5 software,[70] intensities were integrated in SAINTS[71] and absorption corrections based on equivalent reflections were applied using SADABS.[72] The structure was solved using ShelXT[73] structure solution program using Intrinsic Phasing and refined by full matrix least squares against F^2 in ShelXL[74] using Olex2.[75] The time-resolved measurements were obtained using a Stanford Computer Optics 4Picos gated iCCD camera (250-950 nm) with sub-nanosecond resolution and spectrograph equipped with 300 lines/mm grating, 500 nm blaze wavelength system. For the temperature-dependent measurements, a helium-closed cycle cryopump was used, equipped with optical windows, Si thermodiode, and sample mount, attached directly to the cold head. Excitation was from the 3rd or 4th harmonic of an EKSPLA 200 ps Nd:YAG 10 Hz repetition rate laser. Time-resolved measurements were made using a variable CCD gate and delay times relative to the laser trigger, allowing emission decays to be constructed from the changes in spectrum area (normalised by gate time). TCSPC measurements were recorded with a Horiba DeltaFlex TCSPC system using a Horiba NanoLED (357 nm) and SpectraLED (330 nm) as light sources. PLQY measurements were made using a HORIBA Fluorolog-QM spectrofluorometer equipped with a high-efficiency "ECO" friendly continuous 75 W Xenon arc lamp and a HORIBA integrating sphere. The excitation wavelength used was 375 nm, and the monitored emission depended on the molecule. The spectrofluorometer specific parameters were the excitation and emission slits and exits set to 1 nm, the step size for scanning the spectra was 1 nm, and integration time was 0.1 seconds. The PLQY was then calculated using the built in Quantum Yield Calculator of the spectrofluorometer.

2. Sample Preparation for Optical Measurements.

Solutions (in methylcyclohexane (MCH), toluene, and dichloromethane (DCM)) of all the studied samples for photophysical characterization were prepared at low concentration of 2.5-20 μM to strictly prevent intermolecular interactions. Degassed solutions were obtained by 5 freeze-pump-thaw cycles to remove all dissolved oxygen. Solid state samples were fabricated by casting solutions of DCM onto quartz. To prepare the 1 wt. % doped films of emitters in

zeonex, 99 % w/w (99 mg) of the host was dissolved in 0.1 mL of solvent and to this was added 1 % w/w (1 mg) of the emitter.

3. Optical Properties, Structural Characterizations and Theoretical Modelling.

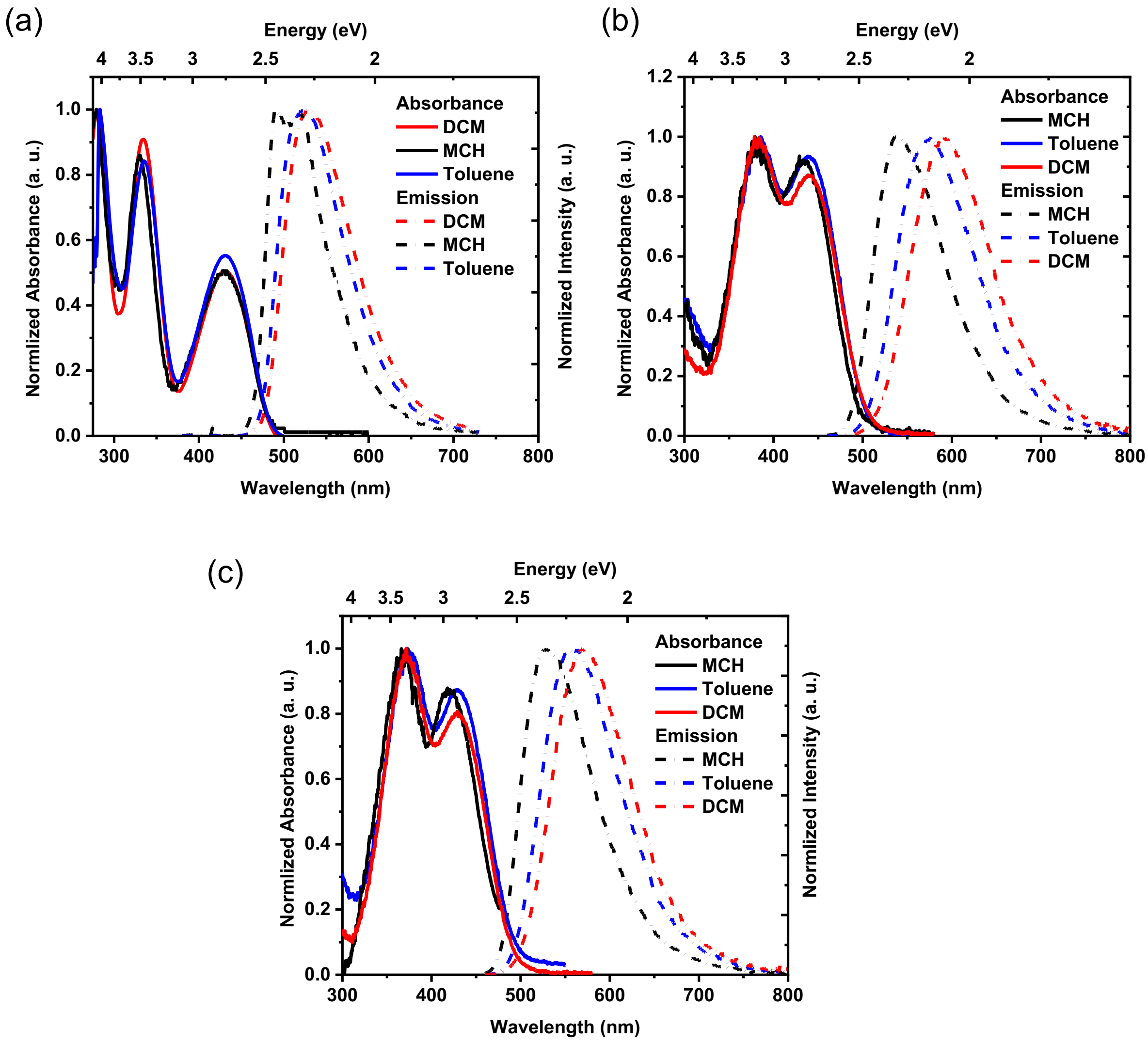

Figure S1. Normalized absorption and steady-state emission spectra measured in different solvents for (a) **CBF2-NO2**, (b) **SBF2-NO2** and (c) **SBF2-CF3**. λ_{exc} = 355 nm, [c] = 1 × 10^{-5} M.

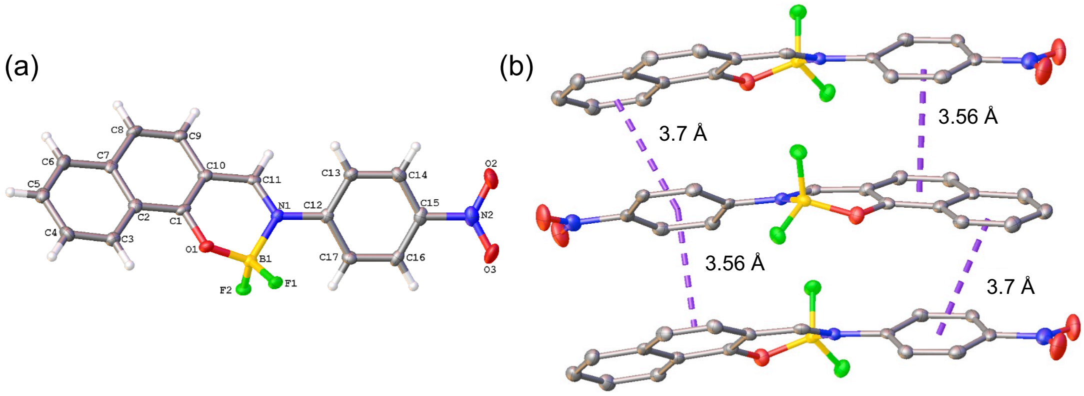

Figure S2. X-ray crystal structure of **CBF2-NO2** shown with (a) the anisotropic displacement parameters depicted at the 50% probability level and (b) the π–π stacking distance between two adjacent molecules and a head-to-tail orientation.

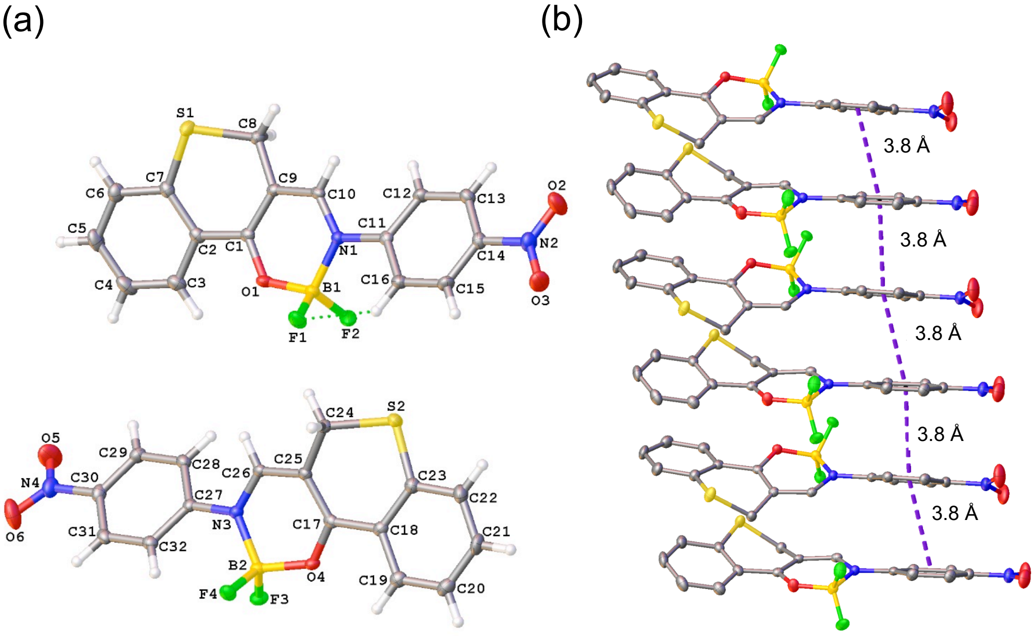

Figure S3. (a) X-ray crystal structure of **SBF2-NO2**, two molecules in the asymmetric unit, with the anisotropic displacement parameters depicted at the 50% probability level. (b) X-ray crystal structure of **SBF2-NO2** showing the π–π stacking distance between two adjacent molecules and a head-to-head orientation. Note: The polymorph reported here for **SBF2-NO2** is different than the previously reported structure.[41]

Table S1. Crystal data and structure refinement for **CBF2-NO2** and **SBF2-NO2**.

Identification code	**CBF2-NO2**	**SBF2-NO2**
Empirical formula	$C_{17}H_{11}BF_2N_2O_3$	$C_{16}H_{11}BF_2N_2O_3S$
CCDC number	2434826	2434827

Formula weight	340.09	360.14
Temperature/K	120.00	120.00
Crystal system	orthorhombic	triclinic
Space group	$P2_12_12_1$	P-1
a/Å	7.2772(3)	7.5842(3)
b/Å	7.7290(3)	14.6714(6)
c/Å	26.0321(12)	15.7222(6)
α/°	90	117.095(2)
β/°	90	91.101(2)
γ/°	90	102.629(2)
Volume/Å^3	1464.19(11)	1505.47(11)
Z	4	4
ρ_{calc}g/cm^3	1.543	1.589
μ/mm^{-1}	0.123	0.258
F(000)	696.0	736.0
Crystal size/mm^3	0.246 × 0.066 × 0.041	0.228 × 0.165 × 0.01
Radiation	Mo Kα (λ = 0.71073)	MoKα (λ = 0.71073)
2Θ range for data collection/°	5.498 to 50.678	5.204 to 52.744
Index ranges	-8 ≤ h ≤ 8, -9 ≤ k ≤ 9, -31 ≤ l ≤ 31	-9 ≤ h ≤ 9, -18 ≤ k ≤ 18, -19 ≤ l ≤ 19
Reflections collected	33677	57623
Independent reflections	2676 [R_{int} = 0.0858, R_{sigma} = 0.0314]	6157 [R_{int} = 0.0773, R_{sigma} = 0.0363]
Data/restraints/parameters	2676/0/226	6157/0/451
Goodness-of-fit on F^2	1.116	1.063
Final R indexes [I>=2σ (I)]	R_1 = 0.0524, wR_2 = 0.1029	R_1 = 0.0440, wR_2 = 0.0904
Final R indexes [all data]	R_1 = 0.0573, wR_2 = 0.1051	R_1 = 0.0560, wR_2 = 0.0952
Largest diff. peak/hole / e Å^{-3}	0.19/-0.31	0.28/-0.30

Note: All non-hydrogen atoms were refined with anisotropic displacement parameters. Hydrogen atoms were located geometrically and refined using a riding model. **CBF2-NO2** has crystallised in a non-centrosymmetric space group but due to the presence of only weak anomalous scatterers, the absolute structure parameter is meaningless and has been removed from the CIF. Crystallographic data for the structures has been deposited with the Cambridge Crystallographic Data Centre with deposition numbers CCDC-2434826 (CBF2-NO2) and 2434827 (SBF2-NO2) respectively. Copies of the data can be obtained free of charge on application to CCDC, 12 Union Road, Cambridge CB2 1EZ, UK [fax(+44) 1223 336033, e-mail: deposit@ccdc.cam.ac.uk].

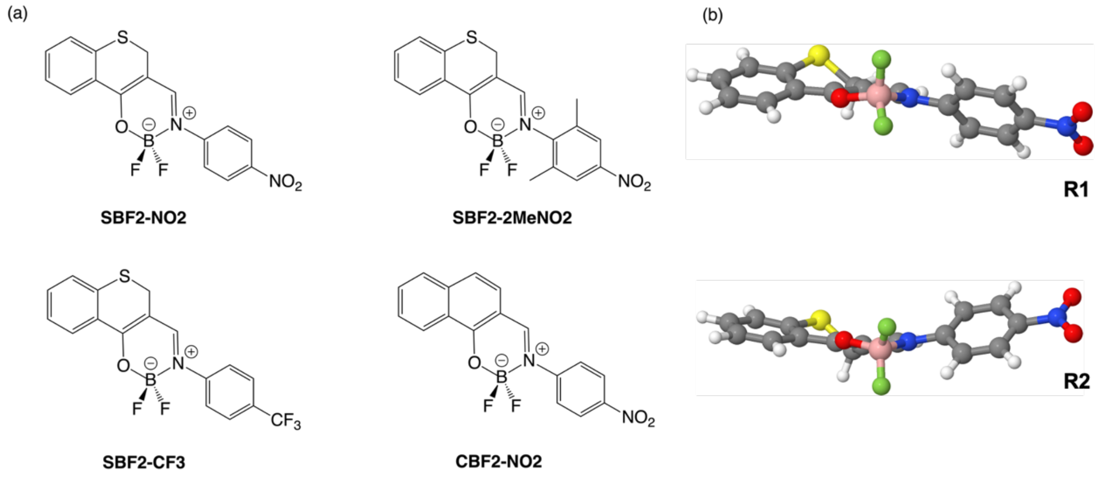

Figure S4. (a) Compounds investigated quantum chemically in this work. (b) Side view of the rotamers R1 (top) and R2 (bottom) of **SBF2-NO2** in the electronic ground state.

Absorption Spectra and State Properties in the Franck–Condon Region

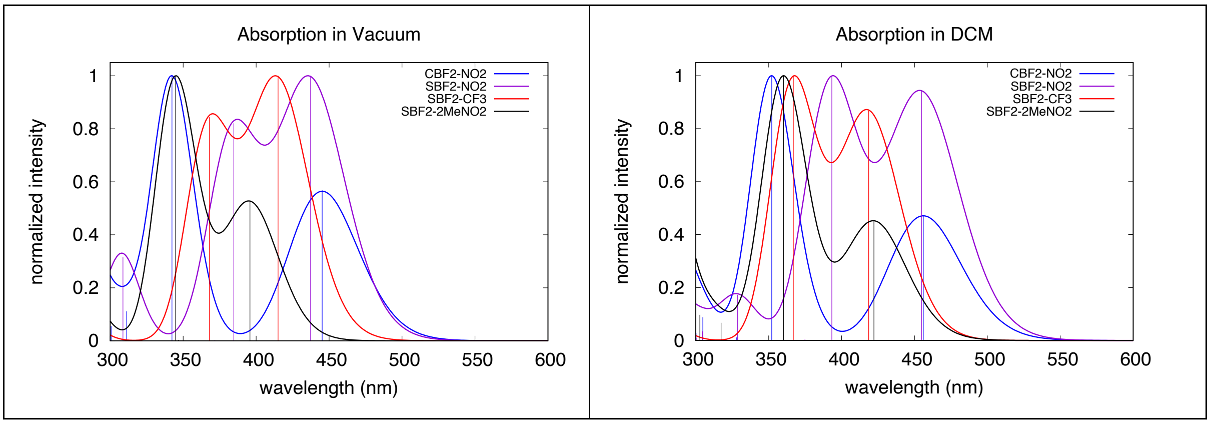

Figure S5. DFT/MRCI absorption spectra in vacuum (left) and DCM (right) normalized to the most intensive transition in the spectral region between 300 and 600 nm. Gaussian functions of 2800 cm^{-1} FWHM were used to broaden the spectra.

Difference densities and computed photophysical properties for the rotamers of **SBF2-NO2**.

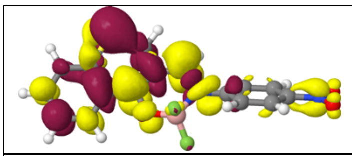	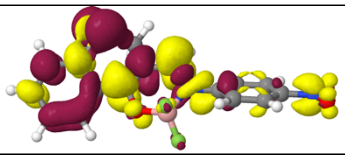	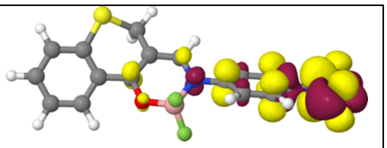
S_1 (72% $\pi_H \rightarrow \pi_L$) @ S_0 geom.	S_2 (73% $\pi_{H-1} \rightarrow \pi_L$) @ S_0 geom.	S_3 ($n_{NO2} \rightarrow \pi^*_{NB}$) @ S_0 geom.
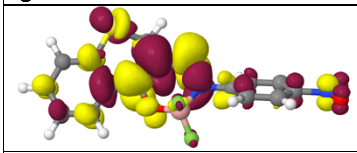	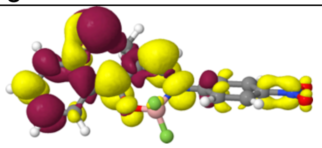	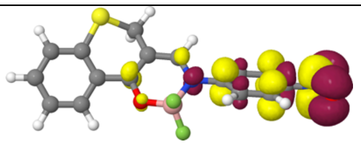
T_1 (41% $\pi_H \rightarrow \pi_L$, 28% $\pi_{H-1} \rightarrow \pi_L$) @ S_0 geom.	T_2 (38% $\pi_{H-1} \rightarrow \pi_L$, 26% $\pi_H \rightarrow \pi_L$) @ S_0 geom.	T_3 ($\pi_{NO2} \rightarrow \pi^*_{NB}$) @ S_0 geom.

Figure S6. Difference electron densities of the excited and ground states of the R1 rotamer of **SBF2-NO2** at the ground-state minimum geometry in DCM (isovalue =±0.001). A gain of electron density upon electronic excitation is indicated in yellow, a loss in red.

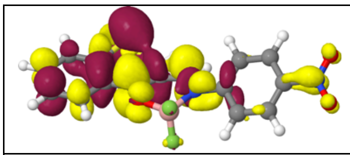	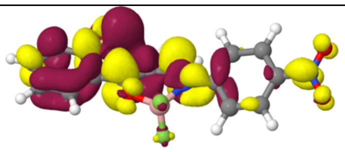	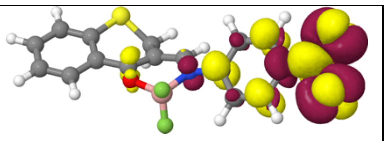
S_1 (71% $\pi_H \rightarrow \pi_L$) @ S_0 geom.	S_2 (74% $\pi_{H-1} \rightarrow \pi_L$) @ S_0 geom.	S_3 ($n_{NO2} \rightarrow \pi^*_{NB}$) @ S_0 geom.
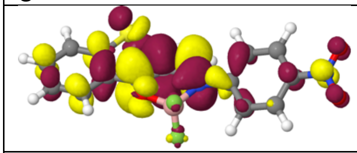	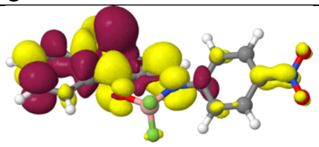	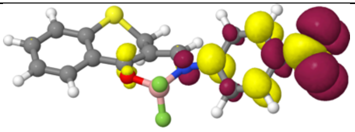
T_1 (39% $\pi_H \rightarrow \pi_L$, 31% $\pi_{H-1} \rightarrow \pi_L$) @ S_0 geom.	T_2 (37% $\pi_{H-1} \rightarrow \pi_L$, 28% $\pi_H \rightarrow \pi_L$) @ S_0 geom.	T_3 ($\pi_{NO2} \rightarrow \pi^*_{NB}$) @ S_0 geom.

Figure S7. Difference electron densities of the excited and ground states of the R2 rotamer of **SBF2-NO2** at the ground-state minimum geometry in DCM (isovalue =±0.001). For colour codes, see Figure S6.

Table S2: DFT/MRCI energies (eV) of the R1 rotamer of **SBF2-NO2** in DCM at various minimum geometries. The S_0 minimum energy serves as common origin. Adiabatic energies are set in bold face.

State\Geometry	@S_0[a]	@S_1	@ S_1'[b]	@T_1	@T_2	@T_1'[c]
S_0	**0.00**	0.30	0.74	0.28	0.23	0.78
S_1 ($\pi_H \rightarrow \pi_L$)	2.73	**2.43**	3.25[d]	2.44	2.61	3.34[d]
S_2 ($\pi_{H-1} \rightarrow \pi_L$)	3.15	3.20	3.66[d]	3.18	2.95	3.73[d]
S_3 ($n_{NO2} \rightarrow \pi^*_{NB}$)	3.31	3.46	**2.48**	3.59	3.51	2.49
T_1 ($\pi_H \rightarrow \pi_L$)	2.15[e]	2.00	2.80[d, e]	**1.96**	2.12[e]	2.88[d, e]
T_2 ($\pi_{H-1} \rightarrow \pi_L$)	2.73[e]	2.72	3.33[d, e]	2.71	**2.51**[e]	3.41[d, e]
T_3 ($\pi_{NO2} \rightarrow \pi^*_{NB}$)	2.97	3.15	2.64	3.26	3.17	2.64
T_4 ($n_{NO2} \rightarrow \pi^*_{NB}$)	3.11	3.29	2.32	3.43	3.33	**2.32**

[a] In the electronic ground state, R2 is marginally preferred over R1 by 0.02 eV. [b] $^1(n_{NO2} \rightarrow \pi^*_{NB})$ geometry. [c] $^3(n_{NO2} \rightarrow \pi^*_{NB})$ geometry. [d] with CT ($Bz \rightarrow NO_2$) contributions. [e] nearly 1:1 mixture of $\pi_H \rightarrow \pi_L$ and $\pi_{H-1} \rightarrow \pi_L$

Table S3: DFT/MRCI energies (eV) of the R2 rotamer of **SBF2-NO2** in DCM at various minimum geometries. The S_0 minimum energy serves as common origin. Adiabatic energies are set in bold face.

State\Geometry	@S_0	@S_1	@S_1'[a]	@T_1	@T_2	@T_1'[b]
S_0	**0.00**	0.29	0.74	0.29	0.21	0.77
S_1 ($\pi_H \rightarrow \pi_L$)	2.73	**2.44**	3.27[c]	2.46	2.62	3.34[c]
S_2 ($\pi_{H-1} \rightarrow \pi_L$)	3.14	3.19	3.65[c]	3.18	2.95	3.72[c]
S_3 ($n_{NO2} \rightarrow \pi^*_{NB}$)	3.31	3.46	**2.48**	3.60	3.49	2.49
T_1 ($\pi_H \rightarrow \pi_L$)	2.17[d]	2.02	2.82[c,d]	**1.98**	2.14[d]	2.90[c,d]
T_2 ($\pi_{H-1} \rightarrow \pi_L$)	2.70[d]	2.71	3.31[c,d]	2.71	**2.50**[d]	3.40[c,d]
T_3 ($\pi_{NO2} \rightarrow \pi^*_{NB}$)	2.96	3.14	2.64	3.26	3.16	2.64
T_4 ($n_{NO2} \rightarrow \pi^*_{NB}$)	3.10	3.29	2.31	3.43	3.32	**2.32**

[a] $^1(n_{NO2} \rightarrow \pi^*_{NB})$ geometry. [b] $^3(n_{NO2} \rightarrow \pi^*_{NB})$ geometry. [c] with CT ($Bz \rightarrow NO_2$) contributions. [d] nearly 1:1 mixture of $\pi_H \rightarrow \pi_L$ and $\pi_{H-1} \rightarrow \pi_L$

Table S4: DFT/MRCI energies (eV) of the R1 rotamer of **SBF2-NO2** at various minimum geometries in vacuum. The S_0 minimum energy serves as common origin. Adiabatic energies are set in bold face.

State\Geometry	@S_0	@S_1	@S_1'[a]	@T_1	@T_2	@T_1'[b]
S_0	**0.00**	0.28	0.67	0.28	0.22	0.79
S_1 ($\pi_H \rightarrow \pi_L$)	2.84	**2.61**	3.49	2.60	2.73	3.54
S_2 ($\pi_{H-1} \rightarrow \pi_L$)	3.22	3.30	3.89	3.17	3.07	3.92
S_3 ($n_{NO2} \rightarrow \pi^*_{NB}$)	3.34	3.50	**2.43**	3.66	3.52	2.45
T_1 ($\pi_H \rightarrow \pi_L$)	2.20[c]	2.13	2.92[c]	**2.00**	2.16[c]	2.95[c]
T_2 ($\pi_{H-1} \rightarrow \pi_L$)	2.81[c]	2.82	3.54[c]	2.79	**2.64**[c]	3.53[c]
T_3 ($\pi_{NO2} \rightarrow \pi^*_{NB}$)	2.93	3.12	2.58	3.25	3.13	2.53
T_4 ($n_{NO2} \rightarrow \pi^*_{NB}$)	3.14	3.31	2.29	3.48	3.35	**2.30**

[a] $^1(n_{NO2} \rightarrow \pi^*_{NB})$ geometry. [b] $^3(n_{NO2} \rightarrow \pi^*_{NB})$ geometry. [c] strong mixture of $\pi_H \rightarrow \pi_L$ and $\pi_{H-1} \rightarrow \pi_L$

**Table S5**: Adiabatic and vertical DFT/MRCI emission energies (eV), vertical emission wavelengths λ_{vert} (nm), static dipole moments μ (Debye), radiative and ISC/rISC rate constants (s^{-1}) of selected states of the R1 rotamer of **SBF2-NO2** in DCM.						
State	ΔE_{adia}	ΔE_{vert}	λ_{vert}	μ	k_r	$k_{ISC/rISC}$
S_0	0.00			10.77		
S_1 ($\pi_H \rightarrow \pi_L$)	2.43	2.13	582	19.73	8×10^7	$\rightarrow T_1$: 3×10^8@77K
						$\rightarrow T_1$: 9×10^8@298K
						$\rightarrow T_2$: 6×10^0@77K
						$\rightarrow T_2$:9×10^7@298K
S_1' ($n_{NO2} \rightarrow \pi^*_{NB}$)	2.48	1.74	712	9.41	6×10^4	$\rightarrow T_1$: 1×10^9@77K
						$\rightarrow T_1$: 6×10^8@298K
T_1 ($\pi_H \rightarrow \pi_L$)	1.96	1.68	741	12.86	1×10^1	
T_2 ($\pi_{H-1} \rightarrow \pi_L$)	2.51	2.28		21.44		$S_1 \leftarrow$: 9×10^9@77K
						$S_1 \leftarrow$: 1×10^{10}@298K
T_1' ($n_{NO2} \rightarrow \pi^*_{NB}$)	2.32	1.54	804	9.35	2×10^2	

**Table S6**: Adiabatic and vertical DFT/MRCI emission energies (eV), vertical emission wavelengths λ_{vert} (nm), static dipole moments μ (Debye), radiative and ISC/rISC rate constants (s^{-1}) of selected states of the R2 rotamer of **SBF2-NO2** in DCM.						
State	ΔE_{adia}	ΔE_{vert}	λ_{vert}	μ	k_r	$k_{ISC/rISC}$
S_0	0.00			10.69		
S_1 ($\pi_H \rightarrow \pi_L$)	2.44	2.15	575	19.49	9×10^7	$\rightarrow T_1$: 3×10^8@77K
						$\rightarrow T_1$: 6×10^8@298K
						$\rightarrow T_2$: 5×10^2@77K
						$\rightarrow T_2$: 5×10^7@298K
S_1' ($n_{NO2} \rightarrow \pi^*_{NB}$)	2.48	1.74	711	9.22	4×10^4	$\rightarrow T_1$: 5×10^8@77K
						$\rightarrow T_1$: 3×10^8@298K
T_1 ($\pi_H \rightarrow \pi_L$)	1.98	1.69	731	12.64	1×10^1	
T_2 ($\pi_{H-1} \rightarrow \pi_L$)	2.50	2.29		21.24		$S_1 \leftarrow$: 2×10^{10}@77K
						$S_1 \leftarrow$: 2×10^{10}@298K
T_1' ($n_{NO2} \rightarrow \pi^*_{NB}$)	2.32	1.55	802	9.16	2×10^2	

Table S7: Adiabatic and vertical DFT/MRCI emission energies (eV), vertical emission wavelengths λ_{vert} (nm), static dipole moments μ (Debye), radiative and ISC/rISC rate constants (s^{-1}) of selected states of the R1 rotamer of **SBF2-NO2** in vacuum.

State	ΔE_{adia}	ΔE_{vert}	λ_{vert}	μ	k_r	$k_{ISC/rISC}$
S_0	0.00			8.60		
S_1 ($\pi_H \rightarrow \pi_L$)	2.61	2.33	531	16.27	1×10^8	$\rightarrow T_1$: 3×10^9 @77K
						$\rightarrow T_1$: 2×10^9 @298K
						$\rightarrow T_2$: 6×10^4 @77K
						$\rightarrow T_2$: 1×10^8 @298K
S_1' ($n_{NO2} \rightarrow \pi^*_{NB}$)	2.43	1.55	798	6.14	1×10^4	$\rightarrow T_1$: 6×10^8 @77K
						$\rightarrow T_1$: 4×10^8 @298K
T_1 ($\pi_H \rightarrow \pi_L$)	2.00	1.72	719	6.95	1×10^1	
T_2 ($\pi_{H-1} \rightarrow \pi_L$)	2.64	2.42		17.49		$S_1 \leftarrow$: 2×10^{10}@77K
						$S_1 \leftarrow$: 2×10^{10}@298K
T_1' ($n_{NO2} \rightarrow \pi^*_{NB}$)	2.30	1.50	826	6.32	1.5×10^2	

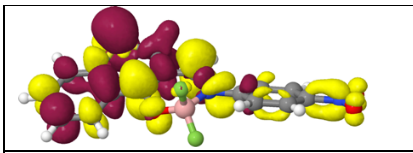	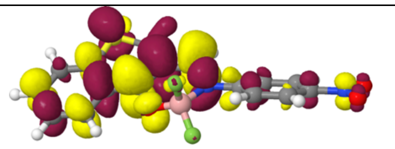	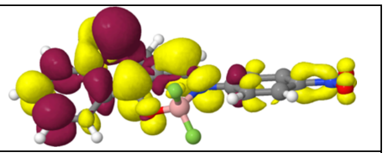
S_1 (79% $\pi_{H}\rightarrow\pi_{L}$) @ S_1 geom.	T_1 (74% $\pi_{H}\rightarrow\pi_{L}$) @ T_1 geom.	T_2 (45% $\pi_{H-1}\rightarrow\pi_{L}$, 32% $\pi_{H}\rightarrow\pi_{L}$) @ T_2 geom.
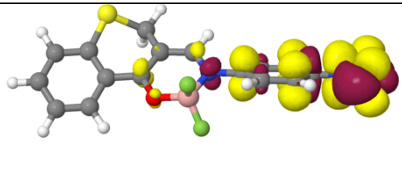	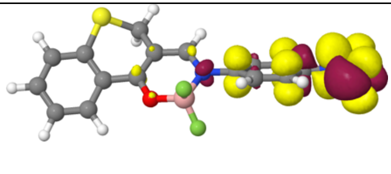	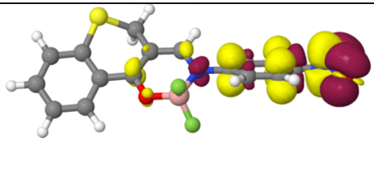
S_1’ ($n_{NO2}\rightarrow \pi^*_{NB}$) @ S_1’ geom.	T_1’ ($n_{NO2}\rightarrow \pi^*_{NB}$) @ T_1’ geom.	T_3 ($\pi_{NO2}\rightarrow \pi^*_{NB}$) @ T_1’ geom.

Figure S8. Difference electron densities of the excited and ground states of the R1 rotamer of **SBF2-NO2** at selected excited-state minimum geometries in DCM (isovalue =±0.001). For colour codes, see Figure S6.

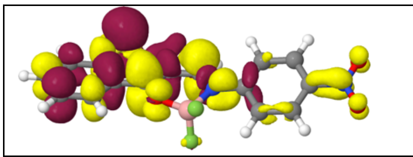	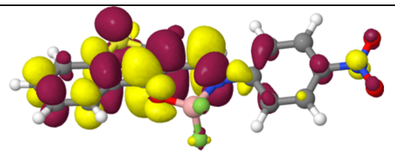	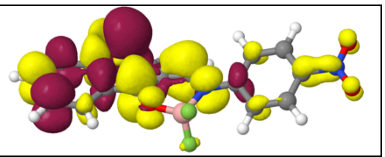
S_1 (79% $\pi_{H}\rightarrow\pi_{L}$) @ S_1 geom.	T_1 (74% $\pi_{H}\rightarrow\pi_{L}$) @ T_1 geom.	T_2 (43% $\pi_{H-1}\rightarrow\pi_{L}$, 34% $\pi_{H}\rightarrow\pi_{L}$) @ T_2 geom.
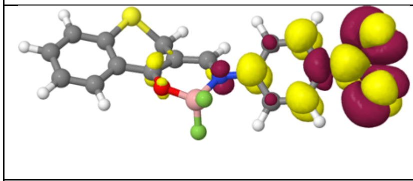	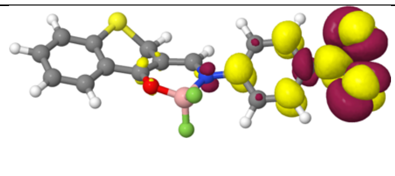	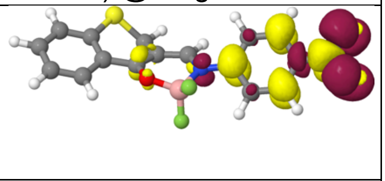
S_1’ ($n_{NO2}\rightarrow\pi^*_{NB}$) @ S_1’ geom.	T_1’ ($n_{NO2}\rightarrow \pi^*_{NB}$) @ T_1’ geom.	T_3 ($\pi_{NO2}\rightarrow \pi^*_{NB}$) @ T_1’ geom.

Figure S9. Difference electron densities of the excited and ground states of the R2 rotamer of **SBF2-NO2** at selected excited-state minimum geometries in DCM (isovalue =±0.001). For colour codes, see **Figure S6.**

Difference densities and computed photophysical properties for **SBF2-CF3**.

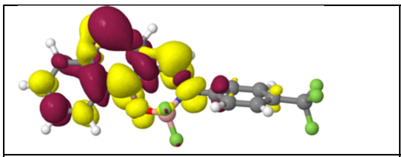	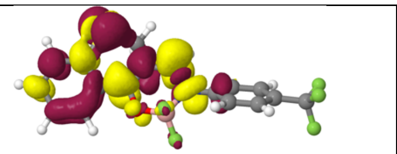	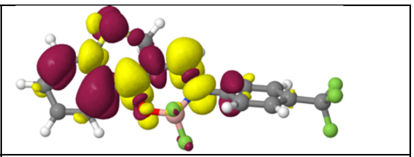
S_1 (86% $\pi_{H}\rightarrow\pi_{L}$) @ S_0 geom.	S_2 (84% $\pi_{H-1}\rightarrow\pi_{L}$) @ S_0 geom.	S_3 (47% $\pi_{H-3}\rightarrow\pi_{L}$) @ S_0 geom.
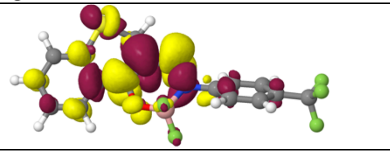	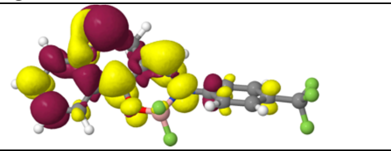	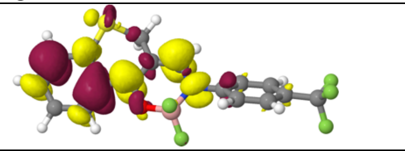
T_1 (53% $\pi_{H}\rightarrow\pi_{L}$, 31% $\pi_{H-1}\rightarrow\pi_{L}$) @ S_0 geom.	T_2 (49% $\pi_{H-1}\rightarrow\pi_{L}$, 32% $\pi_{H-1}\rightarrow\pi_{L}$) @ S_0 geom.	T_3 (45% $\pi_{H-3}\rightarrow\pi_{L}$) @ S_0 geom.

Figure S10. Difference electron densities of the excited and ground states of compound **SBF2-CF3** at the ground-state minimum geometry in DCM (isovalue =±0.001). For colour codes, see Figure S6.

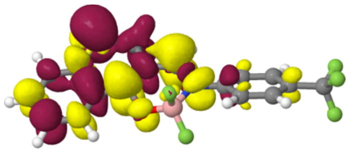

S_1 (86% $\pi_{H}\rightarrow\pi_{L}$) @ S_1 geom.

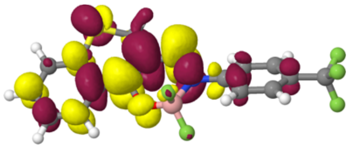

T_1 (81% $\pi_{H}\rightarrow\pi_{L}$) @ T_1 geom.

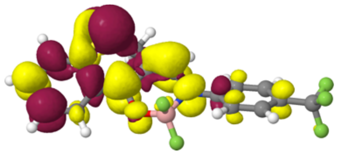

T_2 (58% $\pi_{H-1}\rightarrow\pi_{L}$, 27% $\pi_{H}\rightarrow\pi_{L}$) @ T_2 geom.

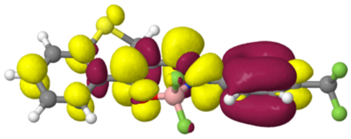

S_3 ($\pi_{H-2}\rightarrow\pi_{L}$) @ S_{CT} geom.

Figure S11. Difference electron densities of the excited and ground states of **SBF2-CF3** at selected excited-state minimum geometries in DCM (isovalue =±0.001). For colour codes, see Figure S6.

Table S8: DFT/MRCI energies (eV) of the R1 rotamer of **SBF2-CF3** in DCM at various minimum geometries. The S_0 energy at the S_0 minimum serves as common origin. Adiabatic energies are set in bold face.

State\Geometry	@S_0	@S_1	@S_{CT}[a]	@T_1	@T_2
S_0	**0.00**	0.26	0.48	0.28	0.14
S_1 ($\pi_H \rightarrow \pi_L$)	2.96	**2.62**	2.99	2.63	2.75
S_2 ($\pi_{H-1} \rightarrow \pi_L$)	3.38	3.36	3.36	3.31	3.12
S_3 ($\pi_{H-3} \rightarrow \pi_L$) ICT (Bz→SBF)	4.32	4.22	4.39	4.18[b]	4.24[b]
S_4 ($\pi_{H-2} \rightarrow \pi_L$) CT (SBF←Ph)	4.42	4.32	**3.94**	4.29	4.26
T_1 ($\pi_H \rightarrow \pi_L$)	2.31[c]	2.12	2.38	**2.06**	2.19[c]
T_2 ($\pi_{H-1} \rightarrow \pi_L$)	2.92[c]	2.85	3.00	2.84	**2.64**[c]
T_3 ($\pi_{H-3} \rightarrow \pi_L$) ICT (Bz→SBF)	3.55	3.64+	3.74	3.60	3.58
T_4 LC (Ph)	3.78	3.86 mix		3.84 mix	mix
T_5 ($\pi_H \rightarrow \pi_{L+1}$)	4.00	4.04	4.32	4.09	3.99
T_6 ($\pi_{H-2} \rightarrow \pi_L$) CT (SBF←Ph)	4.19	4.15	3.79	4.14	4.10

[a] [1]CT (SBF←Ph) geometry. [b] mixed with LC Ph [c] nearly 1:1 mixture of $\pi_H \rightarrow \pi_L$ and $\pi_{H-1} \rightarrow \pi_L$

Table S9: Adiabatic and vertical DFT/MRCI emission energies (eV), vertical emission wavelengths λ_{vert} (nm), static dipole moments μ (Debye), radiative and ISC/rISC rate constants (s^{-1}) of selected states of compound **SBF2-CF3** in DCM.

State	ΔE_{adia}	ΔE_{vert}	λ_{vert}	μ	k_r	$k_{ISC/rISC}$
S_0	0.00			8.72		
S_1 ($\pi_H \rightarrow \pi_L$)	2.62	2.34	530	15.37	9×10^7	→ T_1: 1×10^9 @77K
						→ T_1: 2×10^9 @298K
						→ T_2: 5×10^5 @77K
						→ T_2: 2×10^8 @298K
S_3 ($\pi_{H-2} \rightarrow \pi_L$) CT (SBF←Ph)	3.94	3.46		9.70		
T_1 ($\pi_H \rightarrow \pi_L$)	2.06	1.78	698	9.50	1×10^1	
T_2 ($\pi_{H-1} \rightarrow \pi_L$)	2.64	2.43		17.38		S_1←: 2×10^{10}@77K
						S_1←: 1×10^{10}@298K

Difference densities and computed photophysical properties for the R1 rotamer of **CBF2-NO2.**

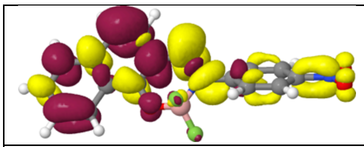	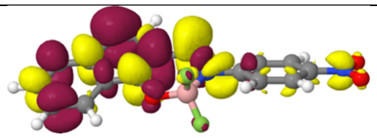	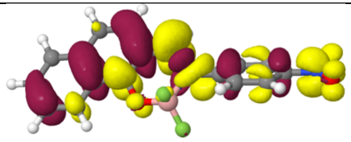
S_1 (68% $\pi_H \rightarrow \pi_L$) @ S_0 geom.	S_2 ($n_{NO2} \rightarrow \pi^*_{NB}$) @ S_0 geom.	S_3 (63% $\pi_{H-1} \rightarrow \pi_L$) @ S_0 geom.
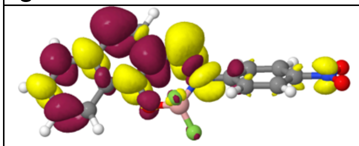	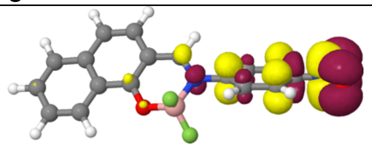	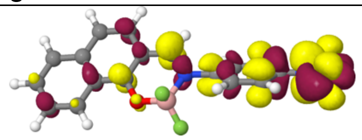
T_1 (60% $\pi_H \rightarrow \pi_L$) @ S_0 geom.	T_2 ($\pi_{NO2} \rightarrow \pi^*_{NB}$) @ S_0 geom.	T_3 ($n_{NO2} \rightarrow \pi^*_{NB}$) @ S_0 geom.
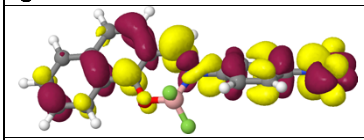		
T_4 (41% $\pi_{H-1} \rightarrow \pi_L$) @ S_0 geom.		

Figure S12. Difference electron densities of the excited and ground states of the R1 rotamer of **CBF2-NO2** at the ground-state minimum geometry in DCM (isovalue =±0.001). For colour codes, see Figure S6.

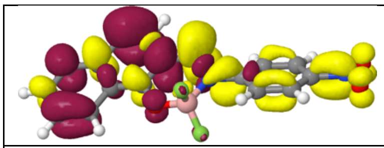	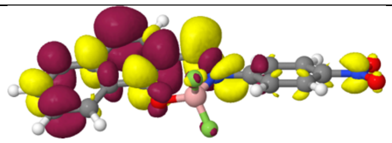	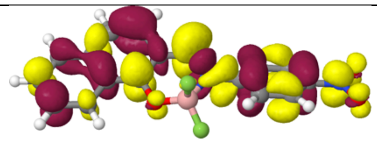
S_1 (72% $\pi_H \rightarrow \pi_L$) @ S_1 geom.	T_1 (74% $\pi_H \rightarrow \pi_L$) @ T_1 geom.	T_2 (74% $\pi_{H-1} \rightarrow \pi_L$) @ T_2 geom.
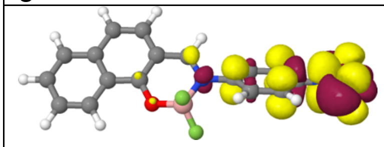	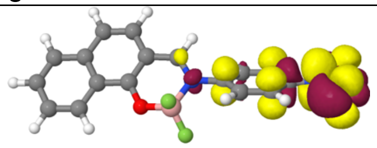	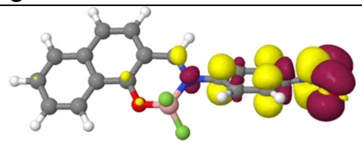
S_1' ($n_{NO2} \rightarrow \pi^*_{NB}$) @ S_1' geom.	T_1' ($n_{NO2} \rightarrow \pi^*_{NB}$) @ T_1' geom.	T_3 ($\pi_{NO2} \rightarrow \pi^*_{NB}$) @ T_1' geom.

Figure S13. Difference electron densities of the excited and ground states of the R1 rotamer of **CBF2-NO2** at selected excited-state minimum geometries in DCM (isovalue =±0.001). For colour codes, see Figure S6.

**Table S10**: DFT/MRCI energies (eV) of the R1 rotamer of **CBF2-NO2** in DCM at various minimum geometries. The S_0 minimum energy serves as common origin. Adiabatic energies are set in bold face.						
State\Geometry	@S_0	@S_1	@ S_1'[a]	@T_1	@T_1'[b]	@T_2
S_0	**0.00**	0.22	0.75	0.20	0.76	0.30
S_1 ($\pi_H \rightarrow \pi_L$)	2.72	**2.45**	3.22	2.49	3.28	2.61
S_2 ($n_{NO2} \rightarrow \pi^*_{NB}$)	3.24	3.21	**2.51**	3.43	2.51	3.44
S_3 ($\pi_{H-1} \rightarrow \pi_L$)	3.52	3.42	3.95	3.38	3.98	3.20
T_1 ($\pi_H \rightarrow \pi_L$)	2.09	1.93	2.73	**1.89**	2.79	2.05
T_2 ($\pi_{NO2} \rightarrow \pi^*_{NB}$)	2.97	2.90	2.64	3.08	2.63	3.12
T_3 ($n_{NO2} \rightarrow \pi^*_{NB}$)	3.03[c]	3.03	2.34	3.23	**2.33**	3.29
T_4 ($\pi_{H-1} \rightarrow \pi_L$)	3.06[c]	2.86	3.40	2.88	3.46	**2.68**
[a] ^{1}($n_{NO2} \rightarrow \pi^*_{NB}$) geometry. [b] ^{3}($n_{NO2} \rightarrow \pi^*_{NB}$) geometry [c] nearly 1:1 mixture of $n_{NO2} \rightarrow \pi^*_{NB}$ and $\pi_{H-1} \rightarrow \pi_L$						

**Table S11**: DFT/MRCI energies (eV) of the R1 rotamer of **CBF2-NO2** in vacuum at various minimum geometries. The S_0 energy at the S_0 minimum serves as common origin. Adiabatic energies are set in bold face.						
State\Geometry	@S_0	@S_1	@ S_1'[a]	@T_1	@T_1'[b]	@T_2
S_0	**0.00**	0.25	0.91	0.20	0.84	0.38
S_1 ($\pi_H \rightarrow \pi_L$)	2.78	**2.53**	3.46	2.57	3.41	2.73
S_2 ($n_{NO2} \rightarrow \pi$)	3.28	3.33	**2.45**	3.46	2.30	3.60
S_3 ($\pi_{H-1} \rightarrow \pi_L$)	3.62	3.60	4.22	3.50	4.19	3.33
T_1 ($\pi_H \rightarrow \pi_L$)	2.14	2.03	2.88	**1.97**	2.82	2.15
T_2 ($\pi_{NO2} \rightarrow \pi$)	2.90	2.96	2.58	3.06	2.57	3.22
T_3 ($n_{NO2} \rightarrow \pi$)	3.09	3.16	2.29	3.28	**2.12**	3.39
T_4 ($\pi_{H-1} \rightarrow \pi_L$)	3.13	3.03	3.68	2.98	3.68	**2.78**
[a] ^{1}($n_{NO2} \rightarrow \pi^*_{NB}$) geometry. [b] ^{3}($n_{NO2} \rightarrow \pi^*_{NB}$) geometry						

**Table S12**: Adiabatic and vertical DFT/MRCI emission energies (eV), vertical emission wavelengths λ_{vert} (nm), static dipole moments μ (Debye), radiative and ISC/rISC rate constants (s^{-1}) of selected states of the R1 rotamer of **CBF2-NO2** in DCM.						
State	ΔE_{adia}	ΔE_{vert}	λ_{vert}	μ	k_r	$k_{ISC/rISC}$
S_0	0.00			10.71		
S_1 ($\pi_H \rightarrow \pi_L$)	2.45	2.23	557	11.29	8×10^7	→ T_1: 2×10^5@77K
						→ T_1: 1×10^5@298K
						→ T_2: 3×10^0@77K
						→ T_2: 1×10^5@298K
S_1' ($n_{NO2} \rightarrow \pi^*_{NB}$)	2.51	1.76	703	9.47	8×10^4	→ T_1: 3×10^9@77K
						→ T_1: 1×10^9@298K
S_2 ($\pi_{H-1} \rightarrow \pi_L$) @$T_2$	(3.20)	(2.90)	(428)	(15.58)	(3×10^8)	
T_1 ($\pi_H \rightarrow \pi_L$)	1.89	1.69	732	16.41	3×10^{-1}	
T_2 ($\pi_{H-1} \rightarrow \pi_L$)	2.68	2.38	521	14.85		S_1←: 6×10^8@77K
						S_1←: 4×10^8@298K
T_1' ($n_{NO2} \rightarrow \pi^*_{NB}$)	2.33	1.57	789	9.47	2×10^2	

Difference densities and computed photophysical properties for **SBF2-2MeNO2.**

For a more general comparison of this family of compounds, we note that through the reduced electron delocalization in the π-system, the absorption spectrum of **SBF2-2MeNO2** is blue shifted with respect to that of **SBF2-NO2**, in agreement with previous experimental observations.[44] The LE states on the dimethylnitrobenzene unit are less affected by the increased twist. Therefore, the optically dark ($n_{NO2} \rightarrow \pi^*_{NB}$) excitation forms the S_2 state in this compound. The impact of the solvent on the energetic positions of the peak maxima is larger for **SBF2-2MeNO2** (red shift about 0.2 eV in DCM) in comparison to **SBF2-NO2**, but the relative intensities of the first two absorption bands remain nearly constant upon solvation.

Due to the nearly perpendicular orientation of the SBF_2 and DMNB π-systems, the charge flow among these units is minimal in **SBF2-2MeNO2** (Figure S14). The twist of the DMNB substituent leads to blueshifts of the T_1, S_1, T_2 and S_2 potentials by about 0.15-0.20 eV relative to **SBF2-NO2**. There are no genuine ($SBF_2 \rightarrow$DMNB) CT states among the low-lying excited states, all charge shifts occur within the SBF_2 moiety, preferentially from the annelated benzene ring to the carbon atoms proximate to O and N. The states involving the NO_2 group closely resemble their counterparts in **SBF2-NO2**, but $^1(n_{NO2} \rightarrow \pi)$ is the second excited singlet state in the FC region. Adiabatically it forms the lowest excited singlet state in vacuum and even in DCM. In the triplet manifold, the $^3(\pi_H \rightarrow \pi_L)$ structure continues to represent the global T_1 minimum.

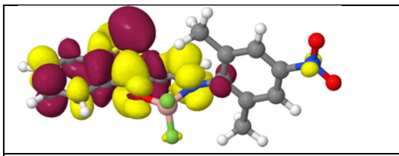	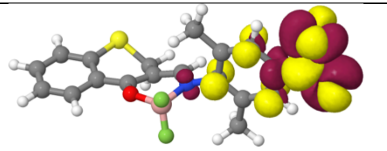	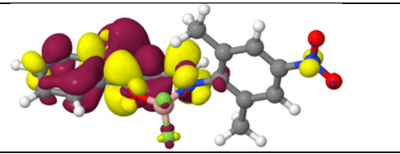
S_1 (64% $\pi_H \rightarrow \pi_L$) @ S_0 geom.	S_2 ($n_{NO2} \rightarrow \pi^*_{NB}$) @ S_0 geom.	S_3 (63% $\pi_{H-1} \rightarrow \pi_L$) @ S_0 geom.
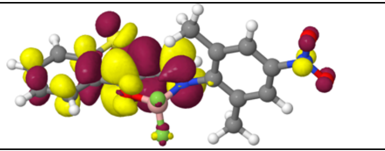	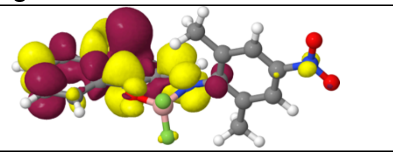	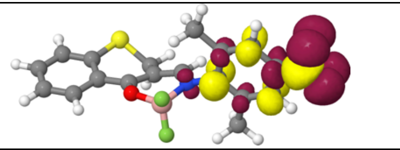
T_1 (38% $\pi_{H-1} \rightarrow \pi_L$, 28% $\pi_H \rightarrow \pi_L$) @ S_0 geom.	T_2 (37% $\pi_H \rightarrow \pi_L$, 25% $\pi_{H-1} \rightarrow \pi_L$) @ S_0 geom.	T_3 ($\pi_{NO2} \rightarrow \pi^*_{NB}$) @ S_0 geom.

Figure S14. Difference electron densities of the excited and ground states of **SBF2-2MeNO2** at the ground-state minimum geometry in DCM (isovalue =±0.001). For colour codes, see Figure S6.

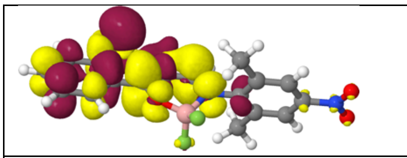	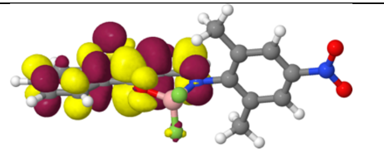	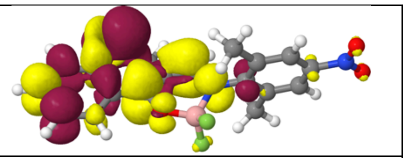
S_1 (80% $\pi_H\rightarrow\pi_L$) @ S_1 geom.	T_1 (75% $\pi_H\rightarrow\pi_L$) @ T_1 geom.	T_2 (20% $\pi_{H-1}\rightarrow\pi_L$, 62% $\pi_H\rightarrow\pi_L$) @ T_2 geom.
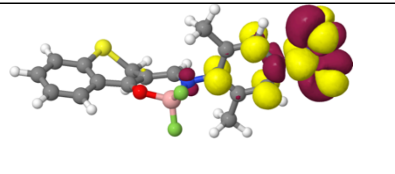	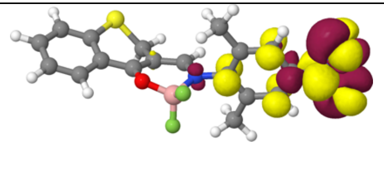	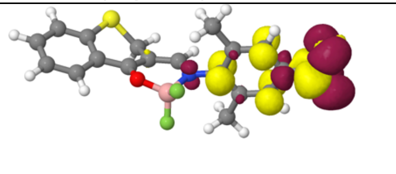
S_1' ($n_{NO2}\rightarrow\pi^*_{NB}$) @ S_1' geom.	T_1' ($n_{NO2}\rightarrow\pi^*_{NB}$) @ T_1' geom.	T_3 ($\pi_{NO2}\rightarrow\pi^*_{NB}$) @ T_1' geom.

Figure S15. Difference electron densities of the excited and ground states of **SBF2-2MeNO2** at selected excited-state minimum geometries in DCM (isovalue =±0.001). For colour codes, see Figure S6.

Table S13: DFT/MRCI energies (eV) of **SBF2-2MeNO2** in DCM at various minimum geometries. The S_0 energy at the S_0 minimum serves as common origin. Adiabatic energies are set in bold face.

State\Geometry	@S_0	@S_1	@S_1'[a]	@T_1	@T_2	@T_1'[b]
S_0	**0.00**	0.26	0.67	0.22	0.14	0.77
S_1 ($\pi_H\rightarrow\pi_L$)	2.93[c]	**2.66**	3.76	2.70	2.86	3.87
S_2 ($n_{NO2}\rightarrow\pi$)	3.37	3.70	**2.63**	3.81	3.67	2.62
S_3 ($\pi_{H-1}\rightarrow\pi_L$)	3.44	3.55		3.56	3.31	
T_1 ($\pi_H\rightarrow\pi_L$)	2.33[c]	2.18	3.18[c]	**2.12**	2.32[c]	3.29[c]
T_2 ($\pi_{H-1}\rightarrow\pi_L$)	2.84[c]	2.93		2.96	**2.67**[c]	3.78[c]
T_3 ($\pi_{NO2}\rightarrow\pi$)	3.01	3.31 (T_4)	2.71	3.40	3.27	2.74
T_4 ($n_{NO2}\rightarrow\pi$)	3.19	3.54 (T_5)	2.47	3.66 (T_5)	3.45	**2.44**

[a] ^{1}($n_{NO2}\rightarrow\pi^*_{NB}$) geometry. [b] ^{3}($n_{NO2}\rightarrow\pi^*_{NB}$) geometry. [c] strong mixture of $\pi_H\rightarrow\pi_L$ and $\pi_{H-1}\rightarrow\pi_L$

Table S14: DFT/MRCI energies (eV) of **SBF2-2MeNO2** in vacuum at various minimum geometries. The S_0 energy at the S_0 minimum serves as common origin. Adiabatic energies are set in bold face.

State\Geometry	@S_0	@S_1	@S_1'[a]	@T_1	@T_2	@T_1'[b]
S_0	**0.00**	0.28	0.95	0.26	0.17	0.94
S_1 ($\pi_H\rightarrow\pi_L$)	3.13	**2.86**	4.04	2.83	2.98	4.05
S_2 ($n_{NO2}\rightarrow\pi$)	3.58	3.80	**2.72**	3.85	3.73	2.68
S_3 ($\pi_{H-1}\rightarrow\pi_L$)	3.60	3.67	4.50	3.58	3.40	4.51
T_1 ($\pi_H\rightarrow\pi_L$)	2.48[c]	2.35	3.44	**2.21**	2.39[c]	3.43
T_2 ($\pi_{H-1}\rightarrow\pi_L$)	3.01[c]	3.04	3.94	3.03	**2.80**[c]	3.94
T_3 ($\pi_{NO2}\rightarrow\pi$)	3.10	3.34	2.76	3.38	3.26	2.75
T_4 ($n_{NO2}\rightarrow\pi$)	3.38	3.60	2.55	3.66	3.54	**2.49**

[a] ^{1}($n_{NO2}\rightarrow\pi^*_{NB}$) geometry. [b] ^{3}($n_{NO2}\rightarrow\pi^*_{NB}$) geometry. [c] strong mixture of $\pi_H\rightarrow\pi_L$ and $\pi_{H-1}\rightarrow\pi_L$

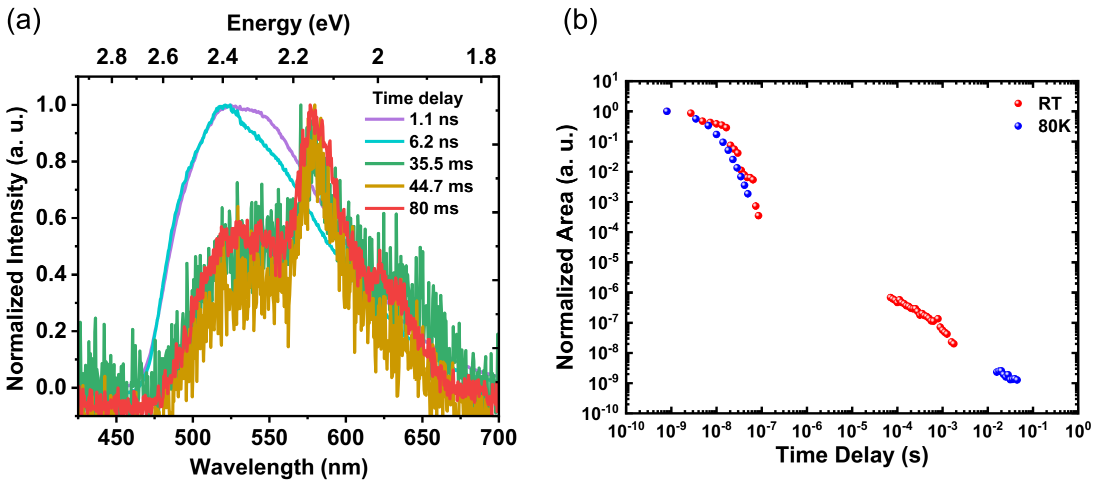

Figure S16. a) Time resolved emission spectra at 80 K obtained at different delay times b) emission decay of **CBF2-NO2** dissolved in dichloromethane. λ_{exc} = 355 nm, [c]= 1 × 10^{-5} M.

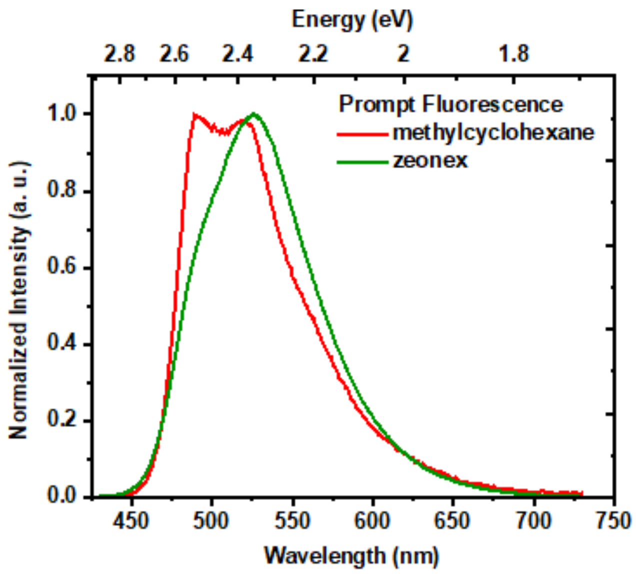

Figure S17. Comparison of prompt fluorescence in 1 wt.% zeonex doped films of **CBF2-NO2** with methylcyclohexane (1 × 10^{-5} M) showing different extent of LE and CT contribution at room temperature. $\lambda_{exc.}$ = 355 nm.

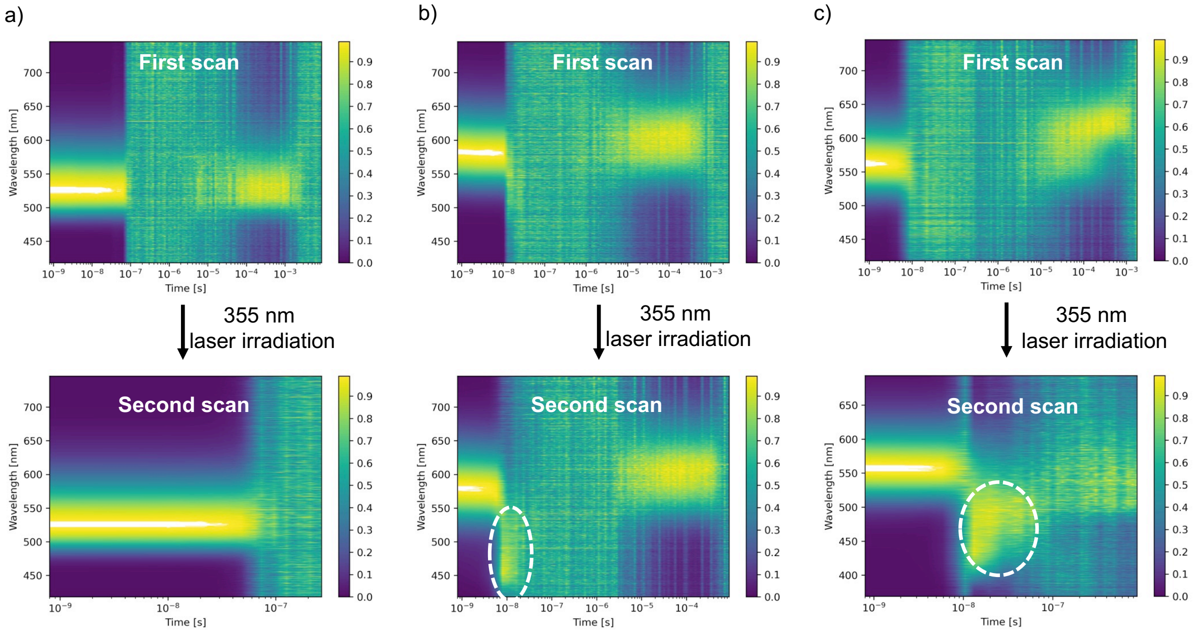

Figure S18. Contour plots of time-resolved emission spectra of (a) **CBF2-NO2**, (b) **SBF2-NO2** and (c) **SBF2-CF3** dissolved in dichloromethane. Bottom panel showing the degradation of same **SBF2-NO2** and **SBF2-CF3** samples between 450-500 nm range, during the second scan, while **CBF2-NO2** does not show such feature indicating no degradation upon photo-irradiation. This is further supported by ^{1}H NMR experiments in Figure S19. (Regions between 100 ns and 10 μs represent hardware background collection, i.e., no detectable emission). λ_{exc} = 355 nm, [c]= 1 × 10^{-5} M.

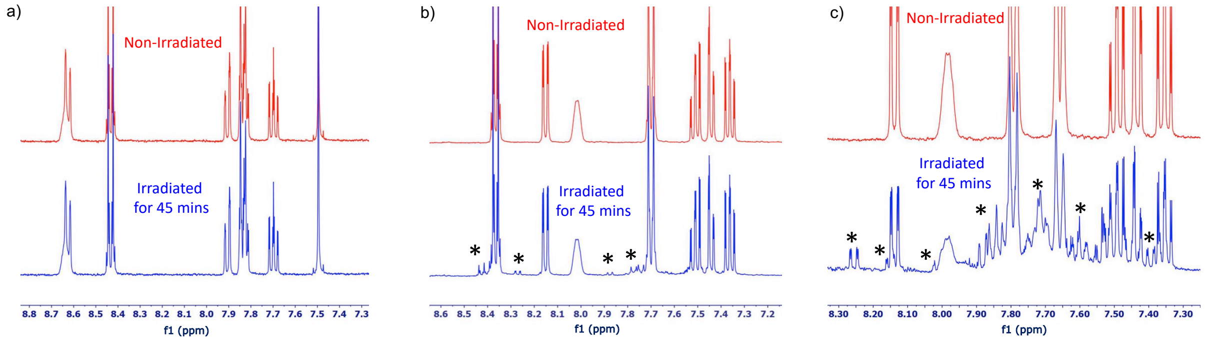

Figure S19. Partial ^{1}H NMR of (a) **CBF2-NO2** and (b) **SBF2-NO2** and (c) **SBF2-CF3** in CD_2Cl_2 (3mg/mL). The photo-irradiation was with 365 nm UV lamp. Clear degradation peaks are indicated with '*' in the bottom panel of (b and c) whereas no such degradation is seen for the **CBF2-NO2**.

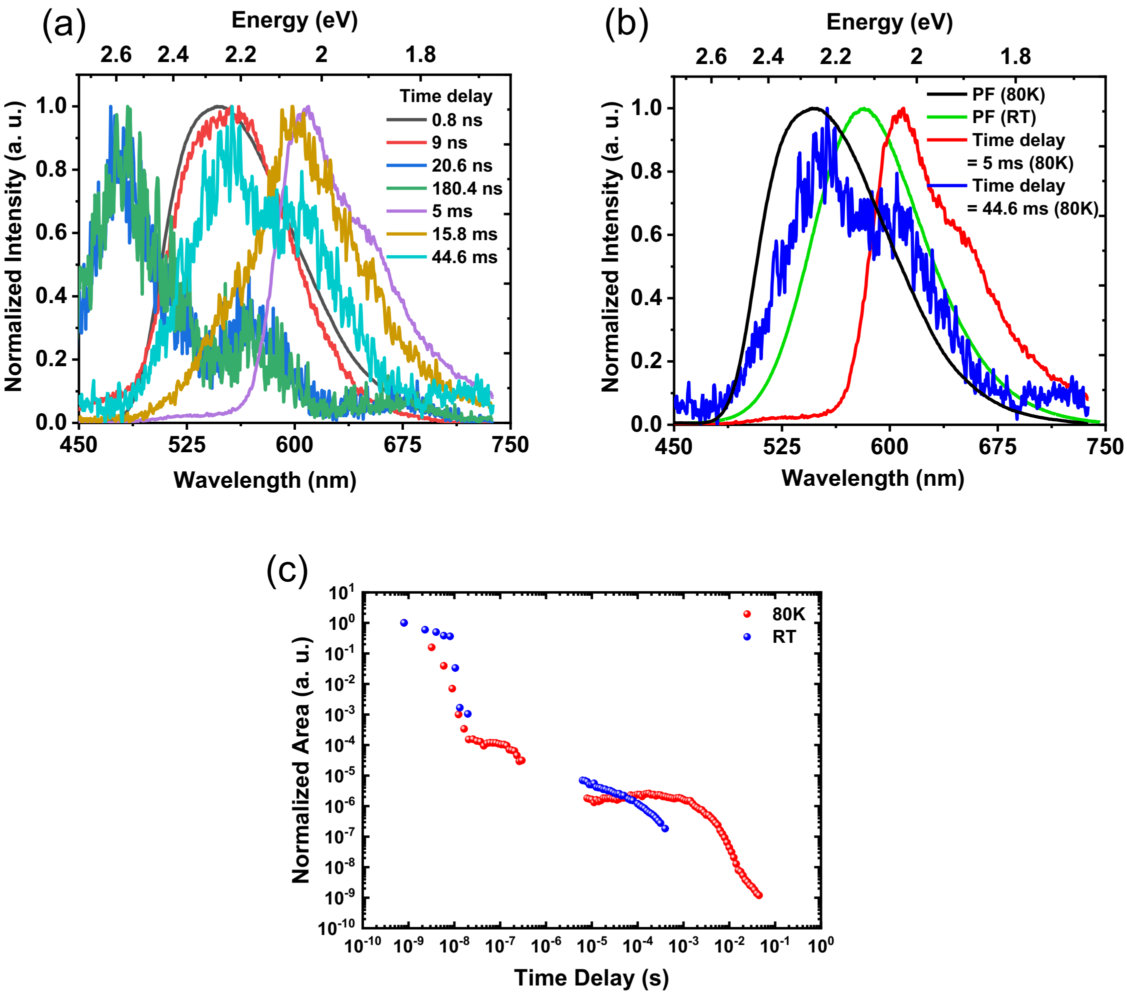

Figure S20. a) Time resolved emission spectra obtained at different delay times of **SBF2-NO2** dissolved in dichloromethane at 80 K. b) Prompt fluorescence and phosphorescence at different time delays. c) Emission decays of the same sample at RT and 80 K. λ_{exc} = 355 nm, [c]= 1 × 10^{-5} M.

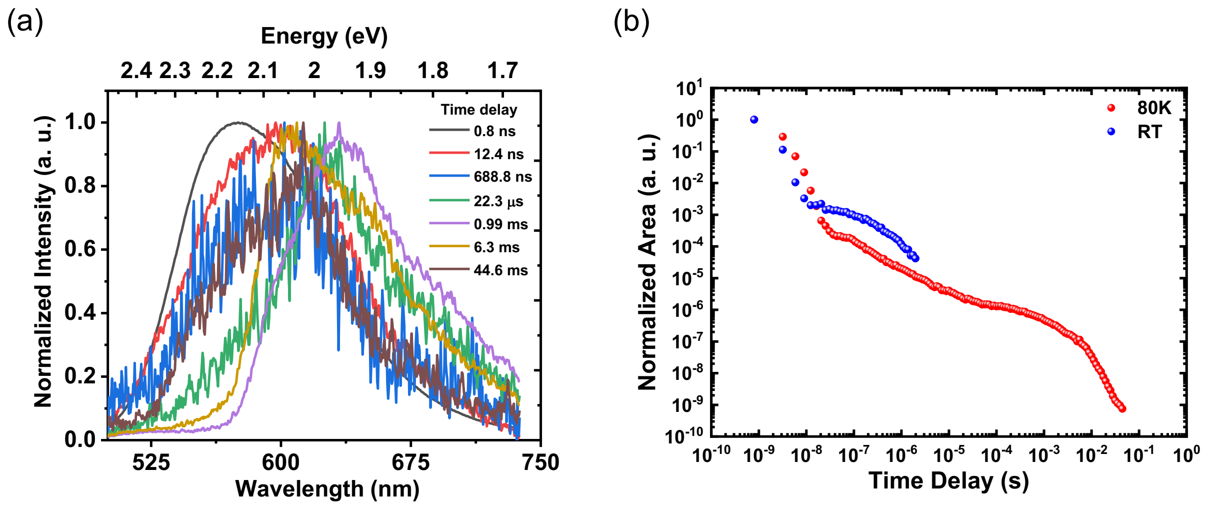

Figure S21. a) Time resolved emission spectra at 80 K obtained at different delay times b) emission decay of 16 mM **SBF2-NO2** dissolved in dichloromethane. λ_{exc} = 355 nm.

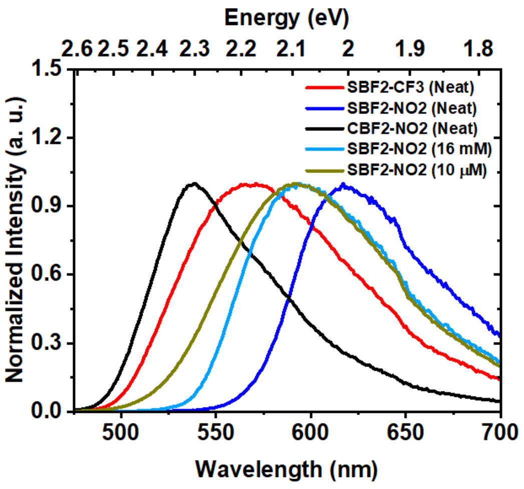

Figure S22. Steady-state emission spectra in neat films of all three compounds at RT. Emission spectra for **SBF2-NO2** dissolved in dichloromethane (10 µM and 16 mM) are also shown for reference. λ_{exc} = 355 nm.

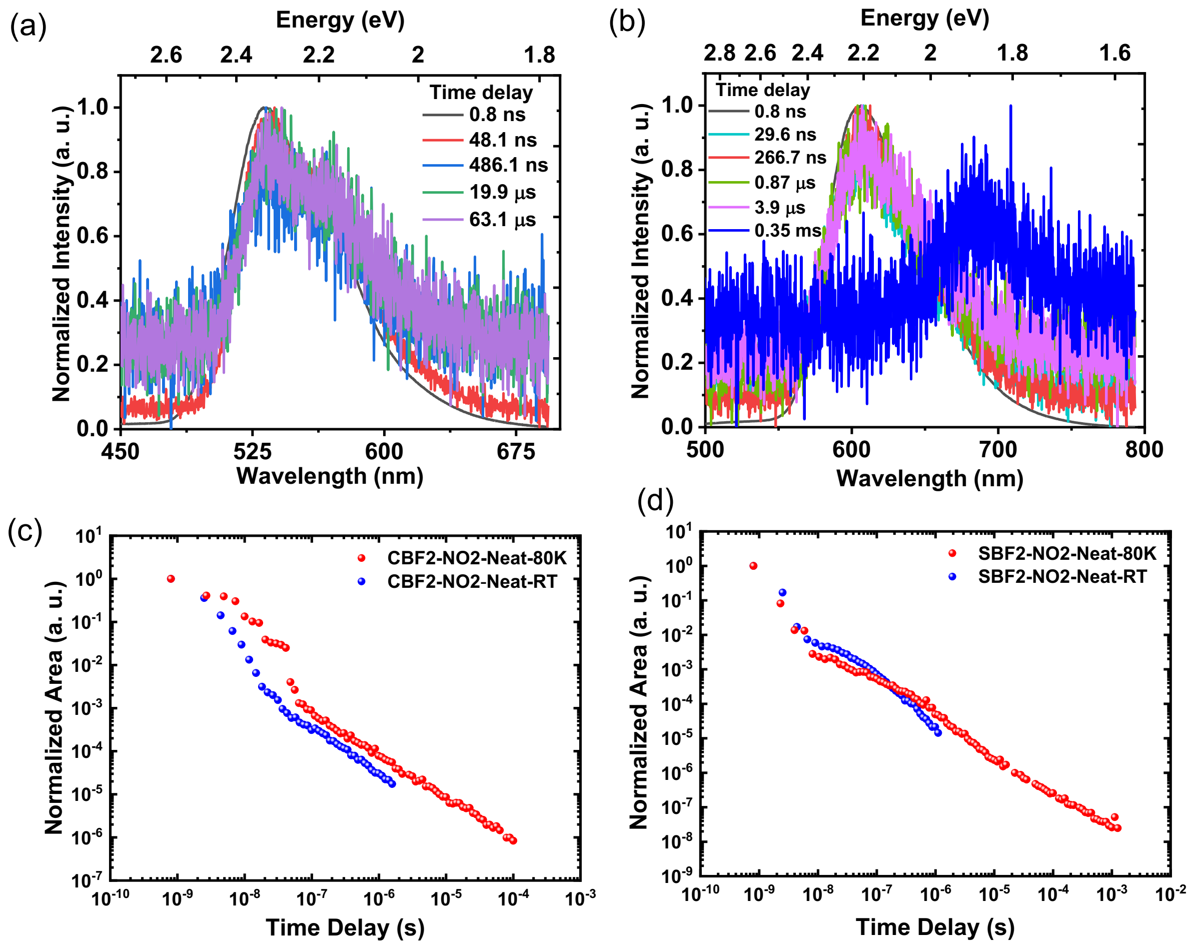

Figure S23. Time resolved spectra at 80 K in neat films of a) **CBF2-NO2** and b) **SBF2-NO2** obtained at different delay times. Emission decays of c) **CBF2-NO2** and d) **SBF2-NO2** in neat film at RT and 80 K. λ_{exc} = 355 nm.

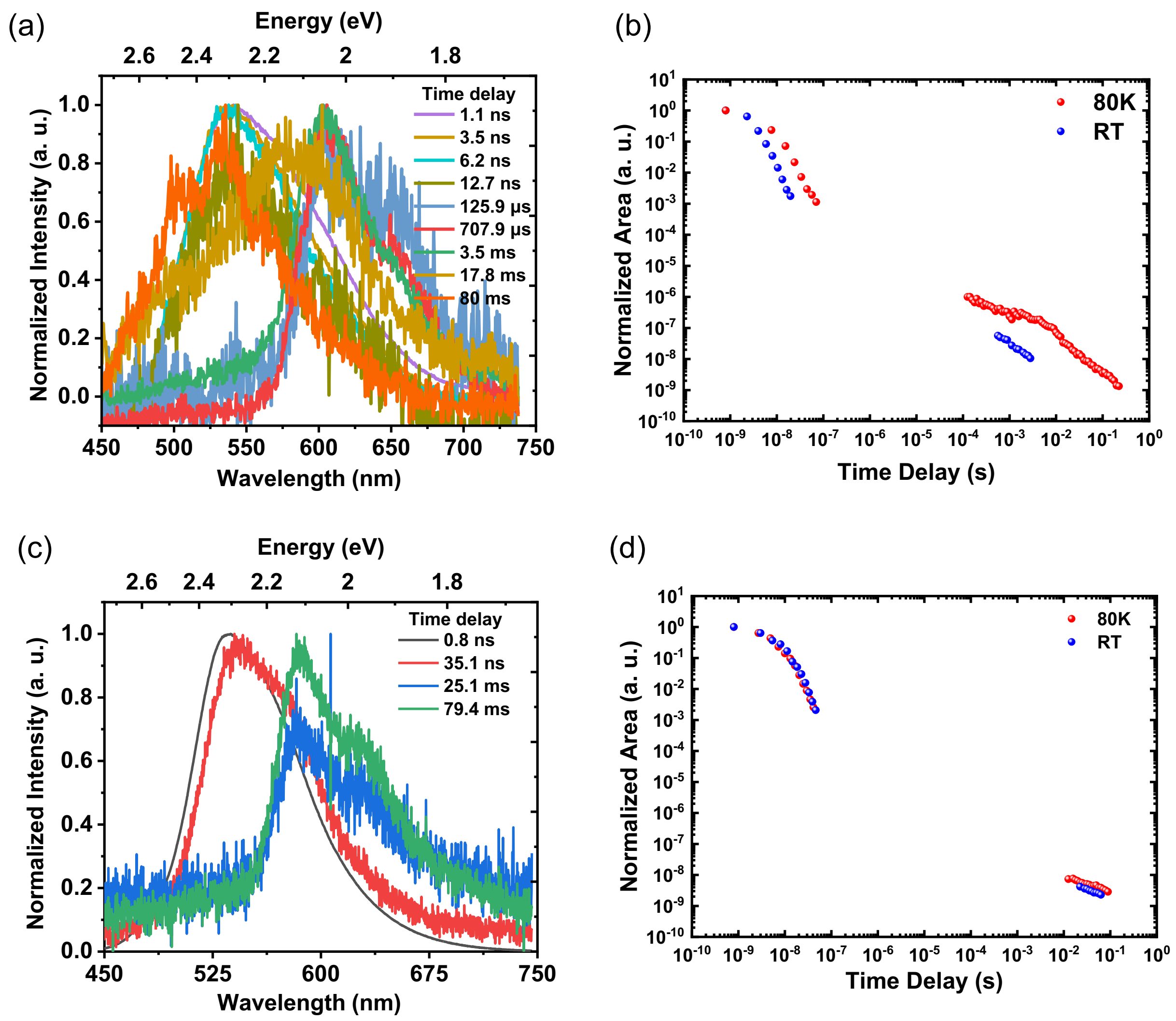

Figure S24. (a) Time resolved emission spectra at 80 K and (b) emission decays obtained at different delay times for **SBF2-NO2** doped in zeonex (1 wt.%). (c) Time resolved emission spectra at 80 K and (d) emission decays obtained at different delay times for **CBF2-NO2** doped in zeonex (1 wt.%). $\lambda_{exc.}$ = 355 nm.

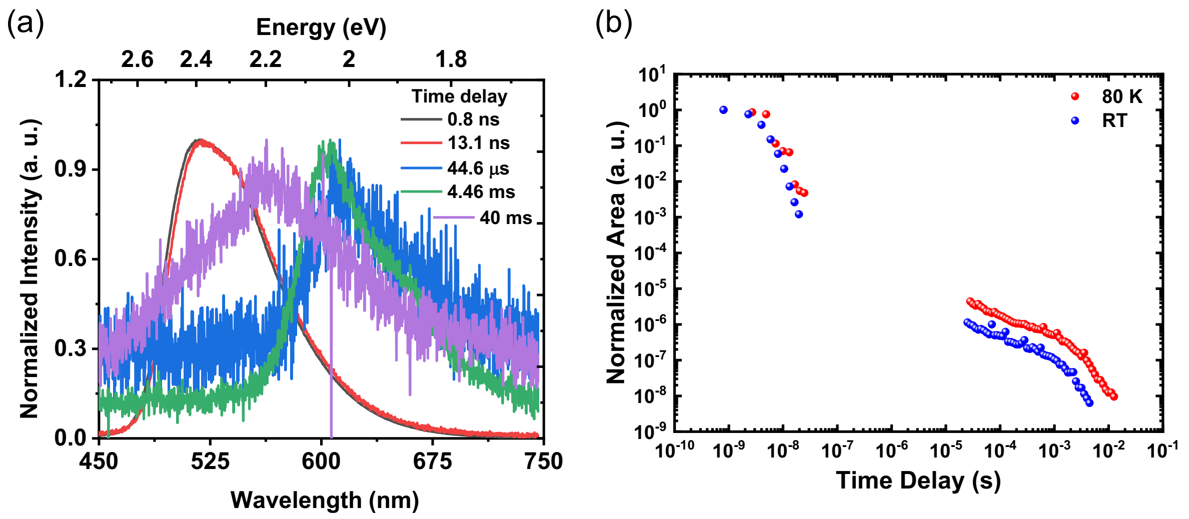

Figure S25. (a) Time resolved emission spectra at 80 K and (b) emission decays for **SBF2-CF3** doped in zeonex (1 wt.%) $\lambda_{exc.}$ = 355 nm. The phosphorescence at 40 ms time delay corresponds to the degradation as has been seen for the **SBF2-NO2** (Figure S24a).

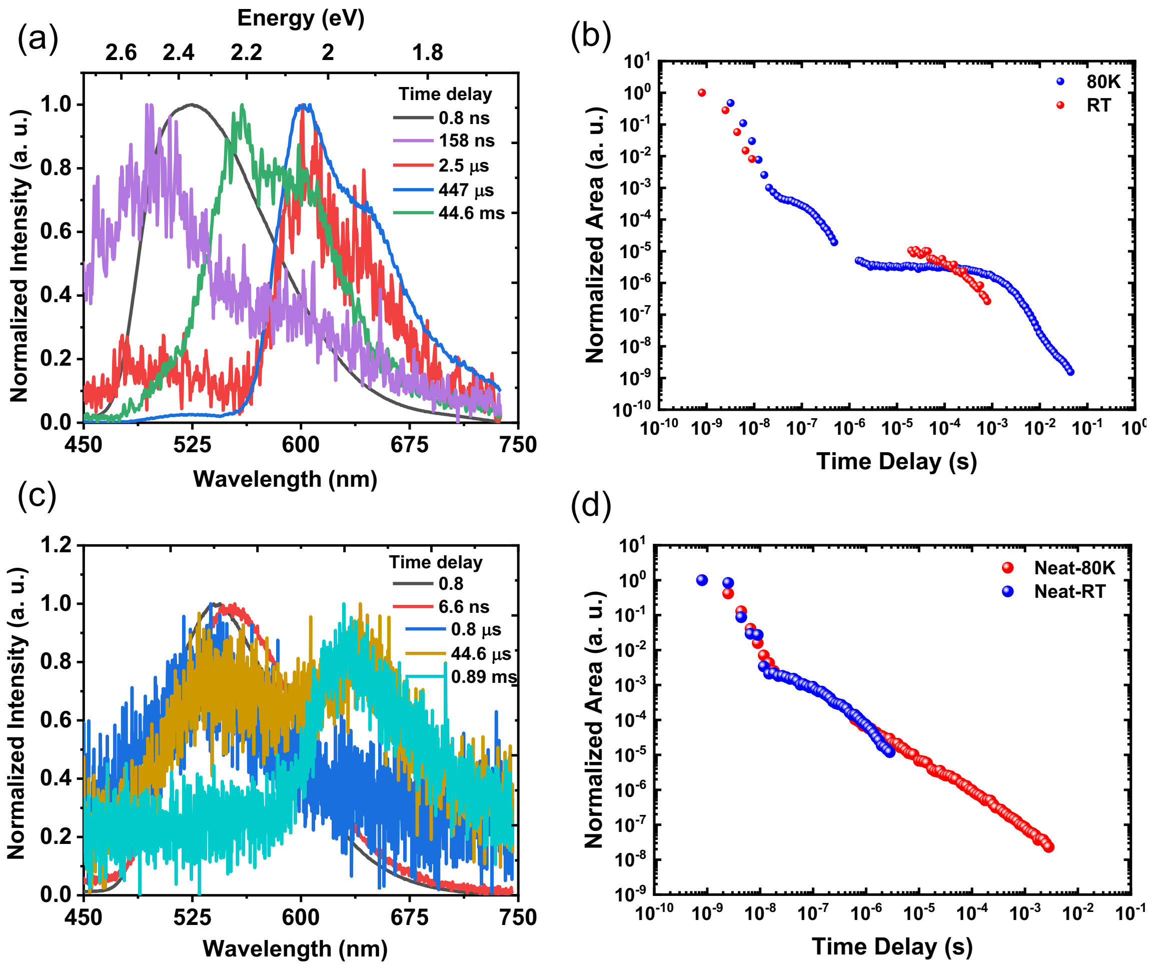

Figure S26. a) Time-resolved emission spectra at different time-delays at 80 K and b) emission decays of **SBF2-CF3** dissolved in dichloromethane λ_{exc} = 355 nm, [c]= 1 × 10^{-5} M. c) Time-resolved emission spectra at different time-delays at 80 K and d) emission decays of **SBF2-CF3** in neat film.

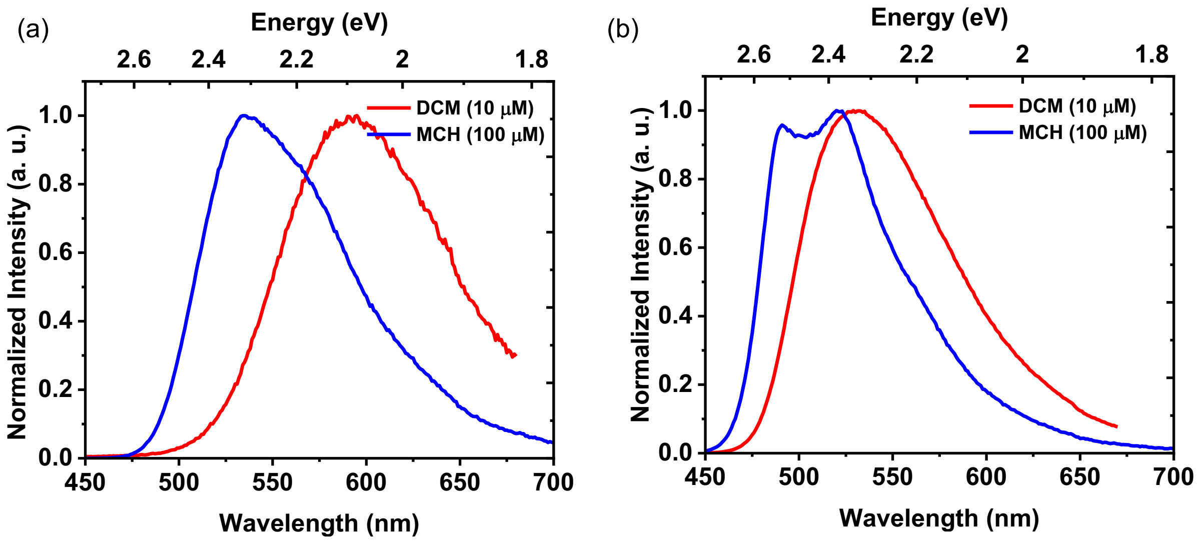

Figure S27. Steady-state emission spectra in DCM and MCH for (a) **SBF2-NO2** and (b) **CBF2-NO2**. λ_{exc} = 355 nm.

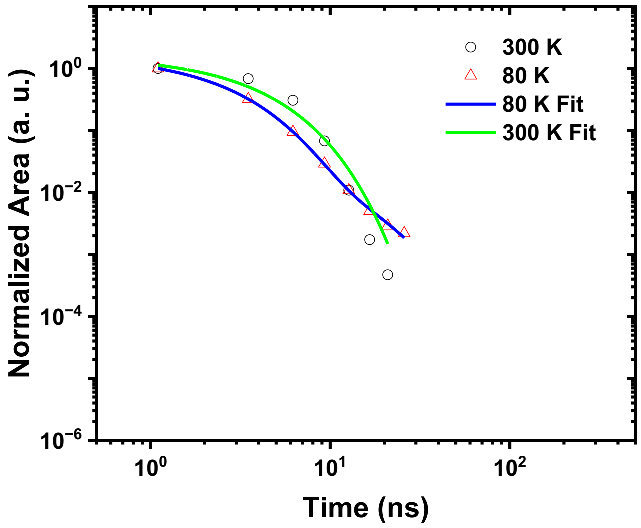

Model	300 K
Equation	y = A1*exp(-x/t1) + y0
Plot	J
y0	0 ± 0
A1	1.63557 ± 0.24656
t1	2.98251 ± 0.31928
Reduced Chi-Sqr	0.02499
R-Square (COD)	0.93912
Adj. R-Square	0.92695

Model	80 K
Equation	y = A1*exp(-x/t1) + A2*exp(-x/t2) + y0
Plot	I
y0	0 ± 0
A1	1.66853 ± 0.01419
t1	2.04431 ± 0.02438
A2	0.02801 ± 0.00618
t2	9.47765 ± 1.08778
Reduced Chi-Sqr	3.59822E-5
R-Square (COD)	0.9999
Adj. R-Square	0.99982

Figure S28. Emission decays (prompt fluorescence region) obtained for **SBF2-NO2** in 100 μM MCH. Fit and fitting parameters of the prompt emission at 300 K and 80 K are also given. (λ_{exc} = 355 nm).

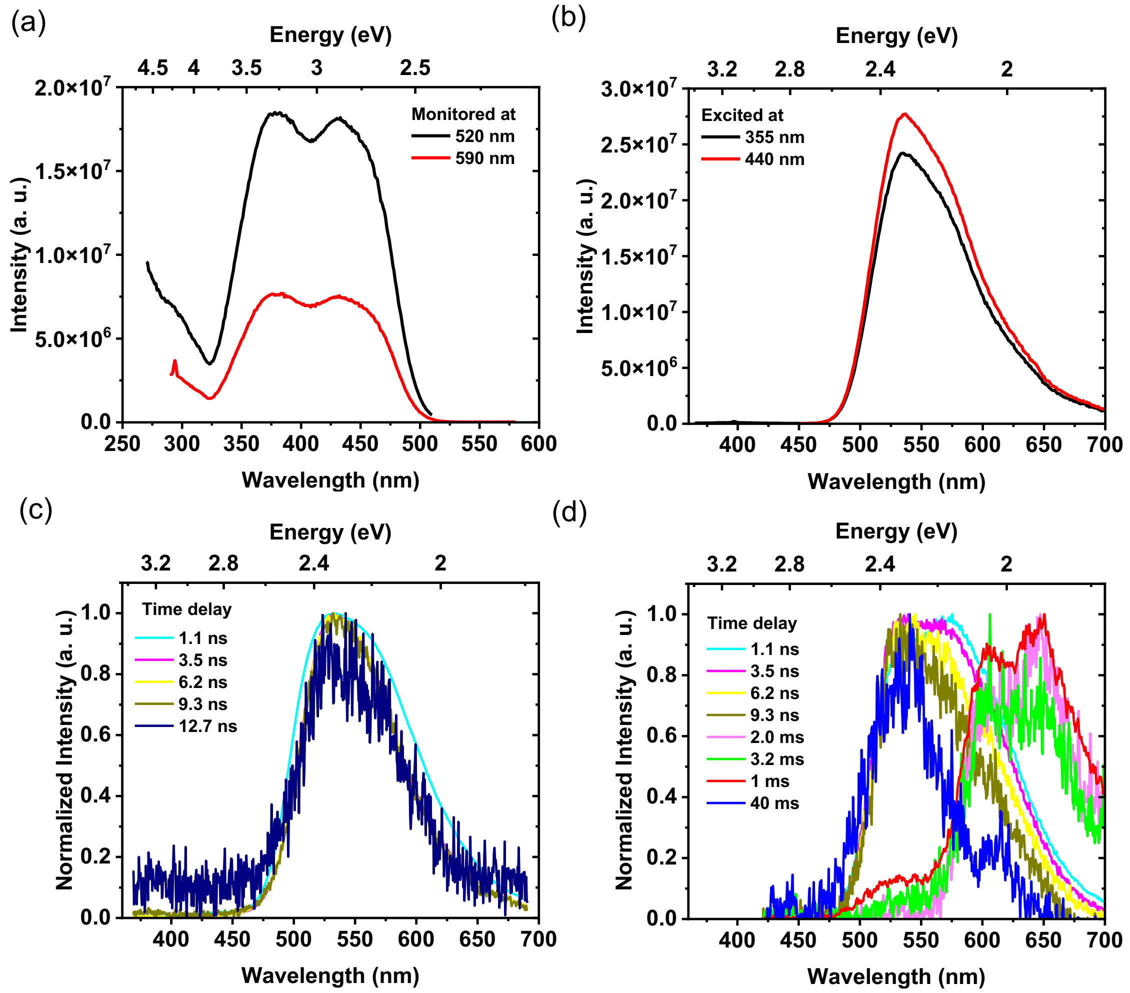

Figure S29. (a) Excitation (b) steady-state emission spectra ($\lambda_{exc.}$ = 355 nm and 440 nm) of **SBF2-NO2** in MCH solution. Time-resolved emission spectra of **SBF2-NO2** ($\lambda_{exc.}$ = 355 nm) at (c) 300 K and (d) 80 K in MCH solution. [c] = 100 μM.

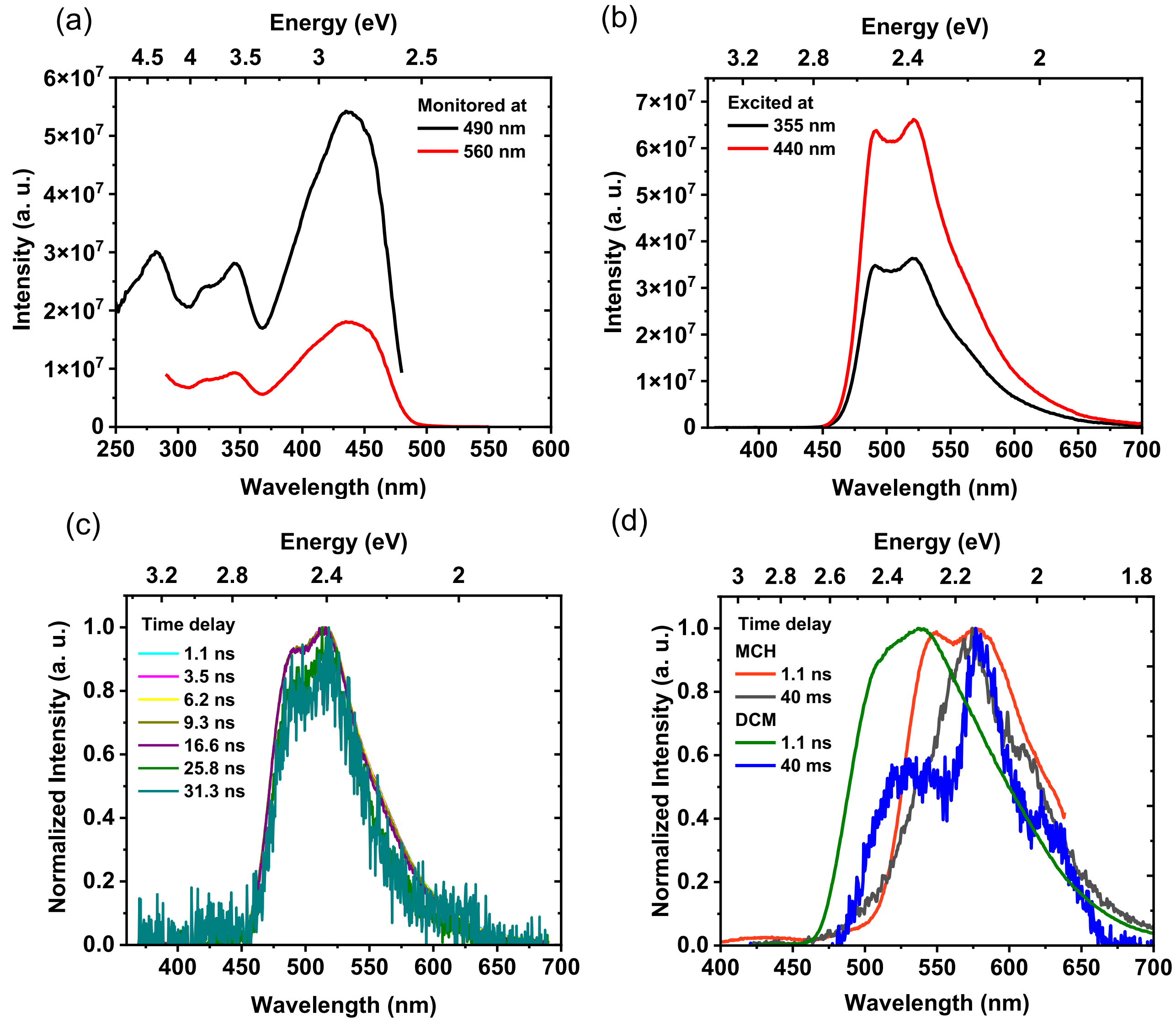

Figure S30. (a) Excitation (b) steady-state emission spectra ($\lambda_{exc.}$ = 355 nm and 440 nm) and (c) time-resolved emission spectra of **CBF2-NO2** ($\lambda_{exc.}$ = 355 nm) at 300 K in MCH solution. [c] = 100 μM. (d) time-resolved emission spectra of **CBF2-NO2** ($\lambda_{exc.}$ = 355 nm) at 80 K in MCH ([c] = 100 μM) and DCM ([c] = 10 μM).

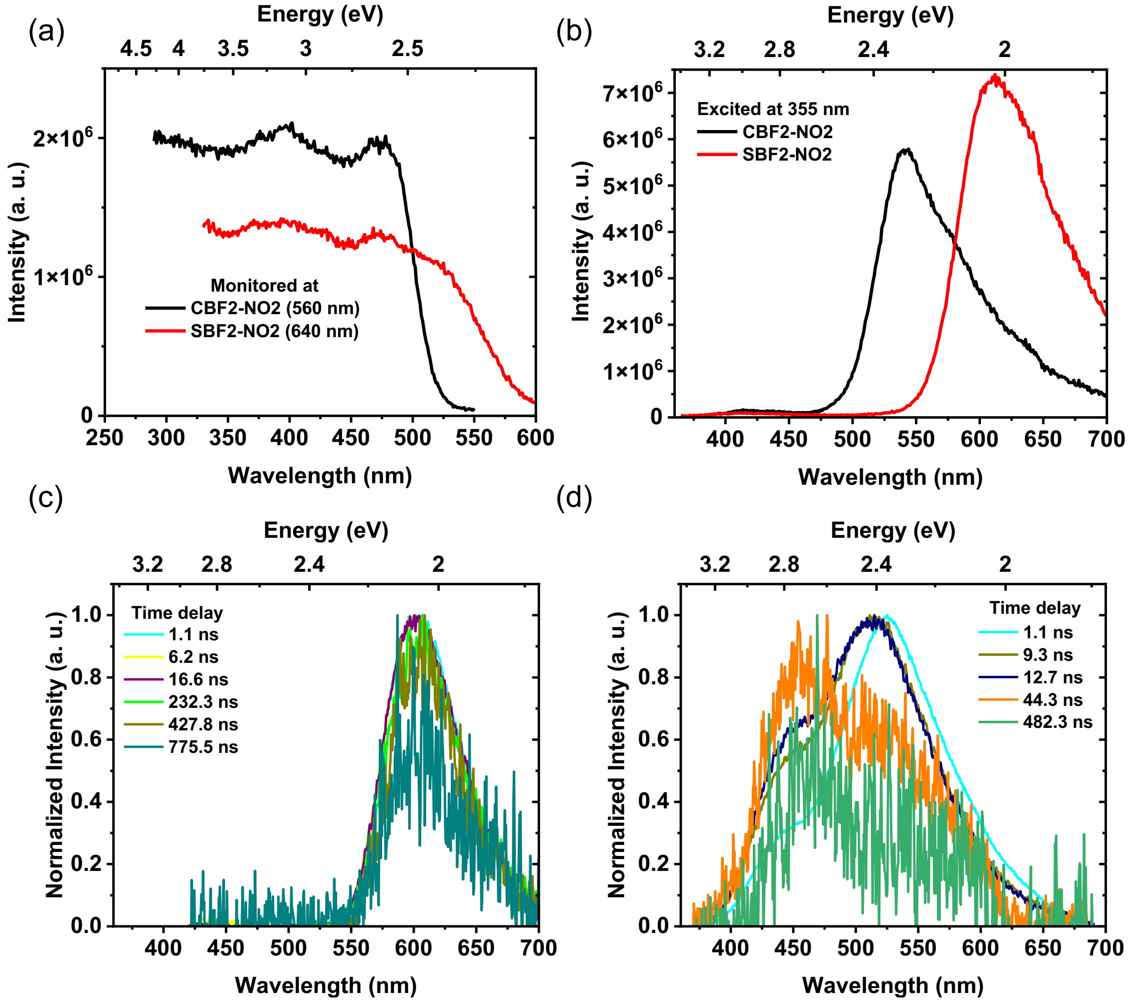

Figure S31. (a) Excitation profile, (b) steady-state emission spectra of **CBF2-NO2** and **SBF2-NO2** ($\lambda_{exc.}$ = 355 nm) at 300 K in crystalline state. Time-resolved emission spectra of (c) **SBF2-NO2** and (d) **CBF2-NO2** crystals at 300 K ($\lambda_{exc.}$ = 355 nm).

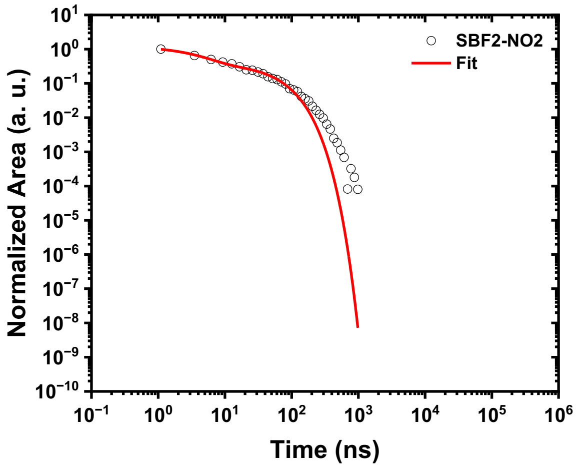

Model	Fit
Equation	y = A1*exp(-x/t1) + A2*exp(-x/t2) + y0
Plot	O
y0	0 ± 0
A1	0.81736 ± 0.02483
t1	3.63333 ± 0.23496
A2	0.3932 ± 0.01516
t2	55.07478 ± 2.95346
Reduced Chi-Sqr	1.71148E-4
R-Square (COD)	0.99674
Adj. R-Square	0.99642

Figure S32. Emission decays obtained for **SBF2-NO2** in crystalline state. Fit and fitting parameters of the prompt emission at 300 K and 80 K are also given. (λ_{exc} = 355 nm).

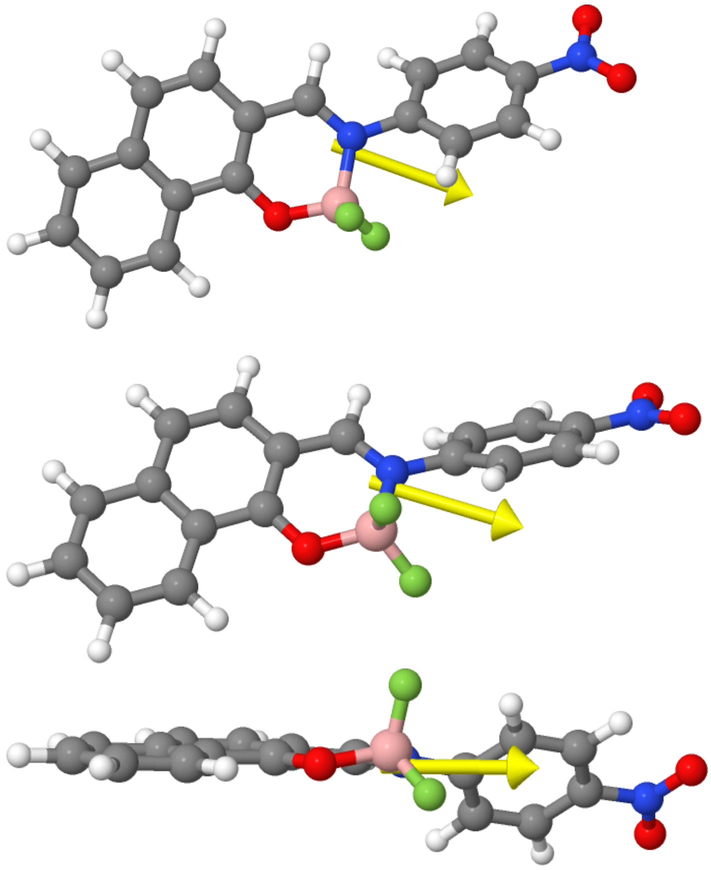

Figure S33. Theoretical monomer transition dipole moment of **CBF2-NO2** in vacuum.

4. Synthetic Scheme and Characterization of the Compounds.

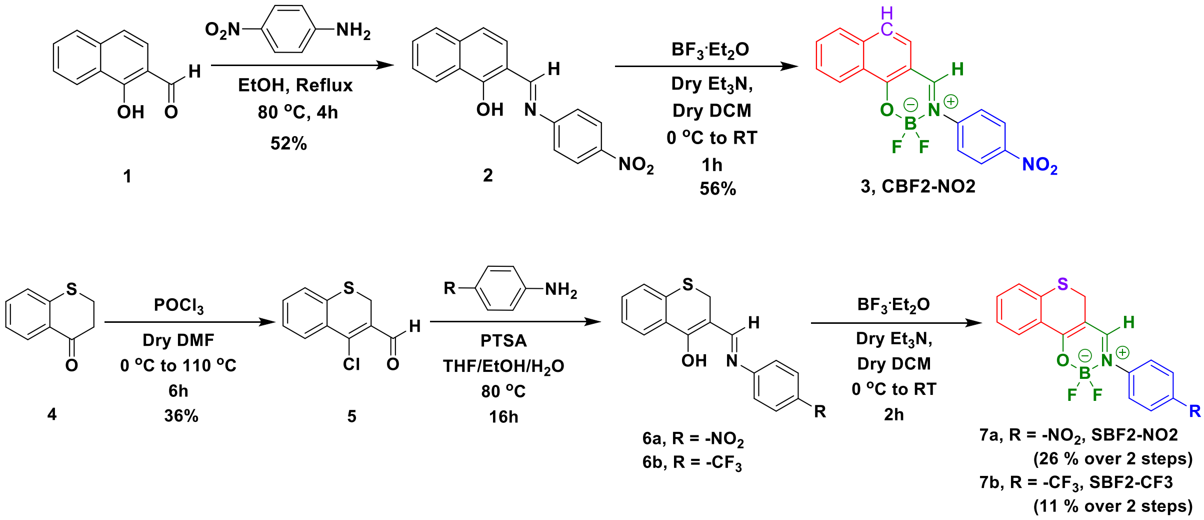

Scheme S1. Synthesis of **CBF2-NO2**, **SBF2-NO2** and **SBF2-CF3**.

All target molecules were synthesized according to a literature procedure. The characterization agrees with the previous reports.[41,45]

CBF2-NO2 (Green solid): ^{1}H NMR (400 MHz, CD_2Cl_2) δ 8.59-8.57 (m, 2H), 8.41 – 8.37 (m, 2H), 7.86 (ddd, *J* = 8.2, 1.3, 0.7 Hz, 1H), 7.83 – 7.74 (m, 3H), 7.66 (ddd, *J* = 8.2, 6.9, 1.3 Hz, 1H), 7.46 (s, 2H). ^{13}C NMR (101 MHz, Acetone-d6) δ 166.97, 161.54, 148.61, 148.55, 140.16, 132.90, 129.12, 127.87, 127.49, 126.00, 125.98, 125.96, 125.81, 125.54, 121.43. ^{11}B NMR (128 MHz, CD_2Cl_2) δ 1.35, 1.23, 1.06. ^{19}F NMR (376 MHz, CD_2Cl_2) δ -133.55, -133.59, -133.64, -133.68. HRMS-ASAP^{+} *m/z* calculated for $[M-F]^{+}$ $C_{17}H_{11}BFN_2O_3$, 321.0847; found: 321.0835

SBF2-NO2 (Red solid): ^{1}H NMR (400 MHz, CD_2Cl_2) δ 8.35 – 8.31 (m, 2H), 8.12 (ddd, *J* = 8.0, 1.6, 0.5 Hz, 1H), 7.98 (s, 1H), 7.70 – 7.63 (m, 2H), 7.47 (ddd, *J* = 8.0, 7.2, 1.5 Hz, 1H), 7.41 (ddd, *J* = 7.9, 1.4, 0.5 Hz, 1H), 7.33 (ddd, *J* = 7.9, 7.2, 1.3 Hz, 1H), 3.86 (s, 2H). ^{13}C NMR (101 MHz, Acetone-d6) δ 169.63, 162.21, 148.59, 147.59, 141.14, 134.52, 129.34, 129.34, 128.58, 127.03, 125.62, 124.90, 124.88, 124.86, 124.95, 103.19, 26.61. ^{11}B NMR (128 MHz, CD_2Cl_2) δ 1.35, 1.23, 1.06. ^{19}F NMR (376 MHz, CD_2Cl_2) δ -133.48, -133.53, -133.57, -133.62. HRMS-ASAP^{+} *m/z* calculated for $[M-F]^{+}$ $C_{16}H_{11}BFN_2O_3S$, 341.0567; found: 341.0560.

SBF2-CF3 (Green solid): ^{1}H NMR (400 MHz, CD_2Cl_2) δ 8.10 (dd, *J* = 7.9, 1.4 Hz, 1H), 7.94 (s, 1H), 7.75 (m, 2H), 7.62 (m, 2H), 7.45-7.39 (m, 2H), 7.31 (ddd, *J* = 7.9, 7.1, 1.4 Hz, 1H), 5.32 (s, 1H), 3.84 (s, 2H). ^{13}C NMR (101 MHz, Acetone-d6) δ 168.98, 162.53, 146.89, 140.96, 134.43, 130.20, 129.55, 129.34, 128.70, 127.60, 127.56, 127.52, 127.48, 127.17, 124.95, 102.96, 26.75. ^{11}B NMR (128 MHz, CD_2Cl_2) δ 1.35, 1.23, 1.06. ^{19}F NMR (376 MHz, CD_2Cl_2) δ -62.85, -133.55, -133.59, -133.64, -133.68. HRMS-ASAP^{+} *m/z* calculated for $[M-F]^{+}$ $C_{17}H_{11}BF_4NOS$, 364.0591; found: 364.0599.

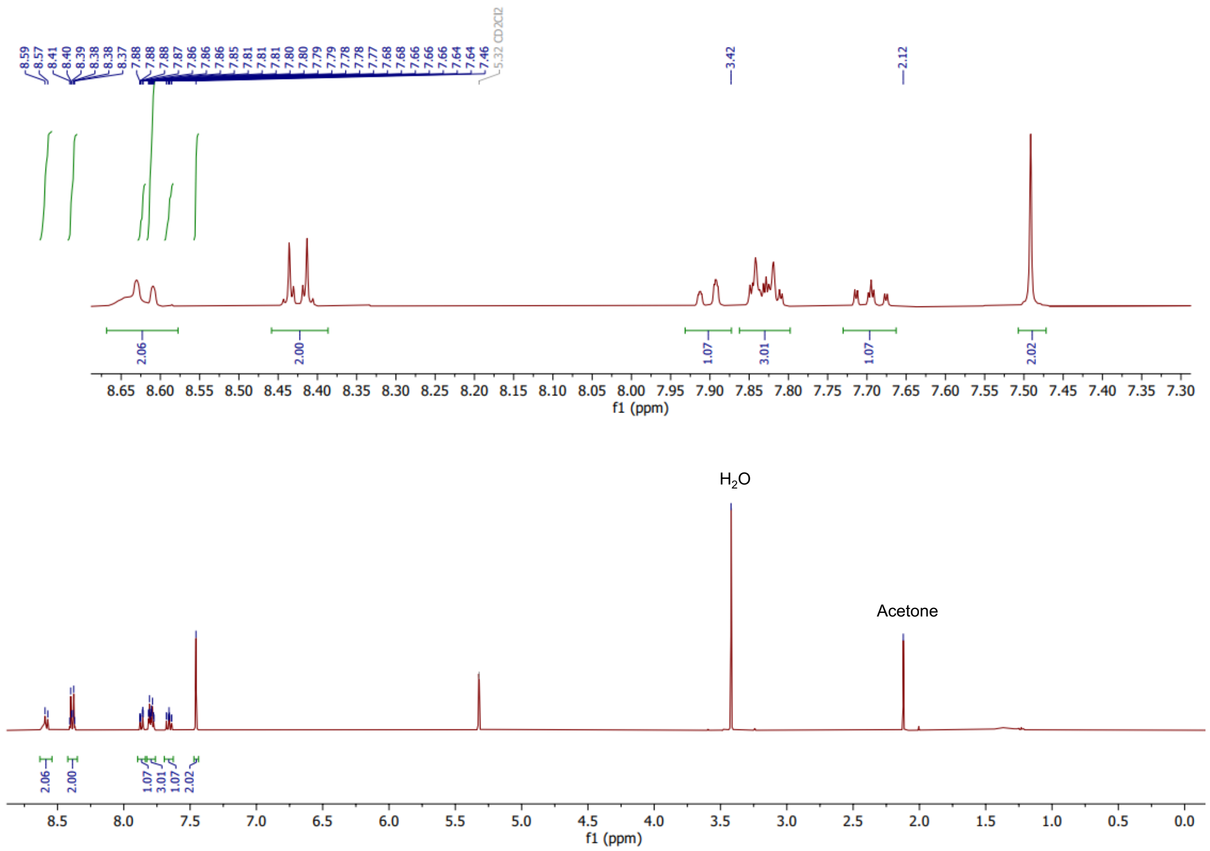

Figure S34. ^{1}H NMR spectrum of **CBF2-NO2** in CD_2Cl_2 at RT.

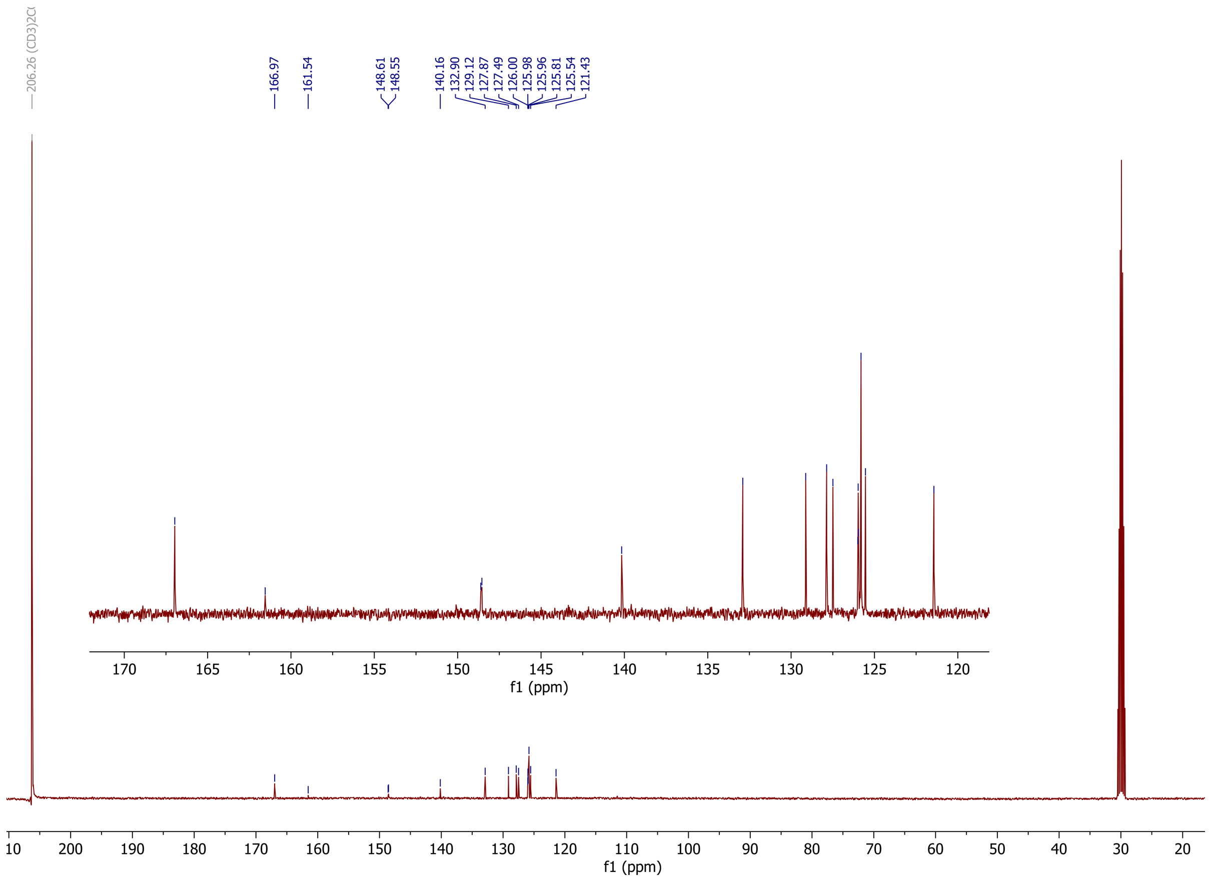

Figure S35. ^{13}C NMR spectrum of **CBF2-NO2** in acetone-d6 at RT.

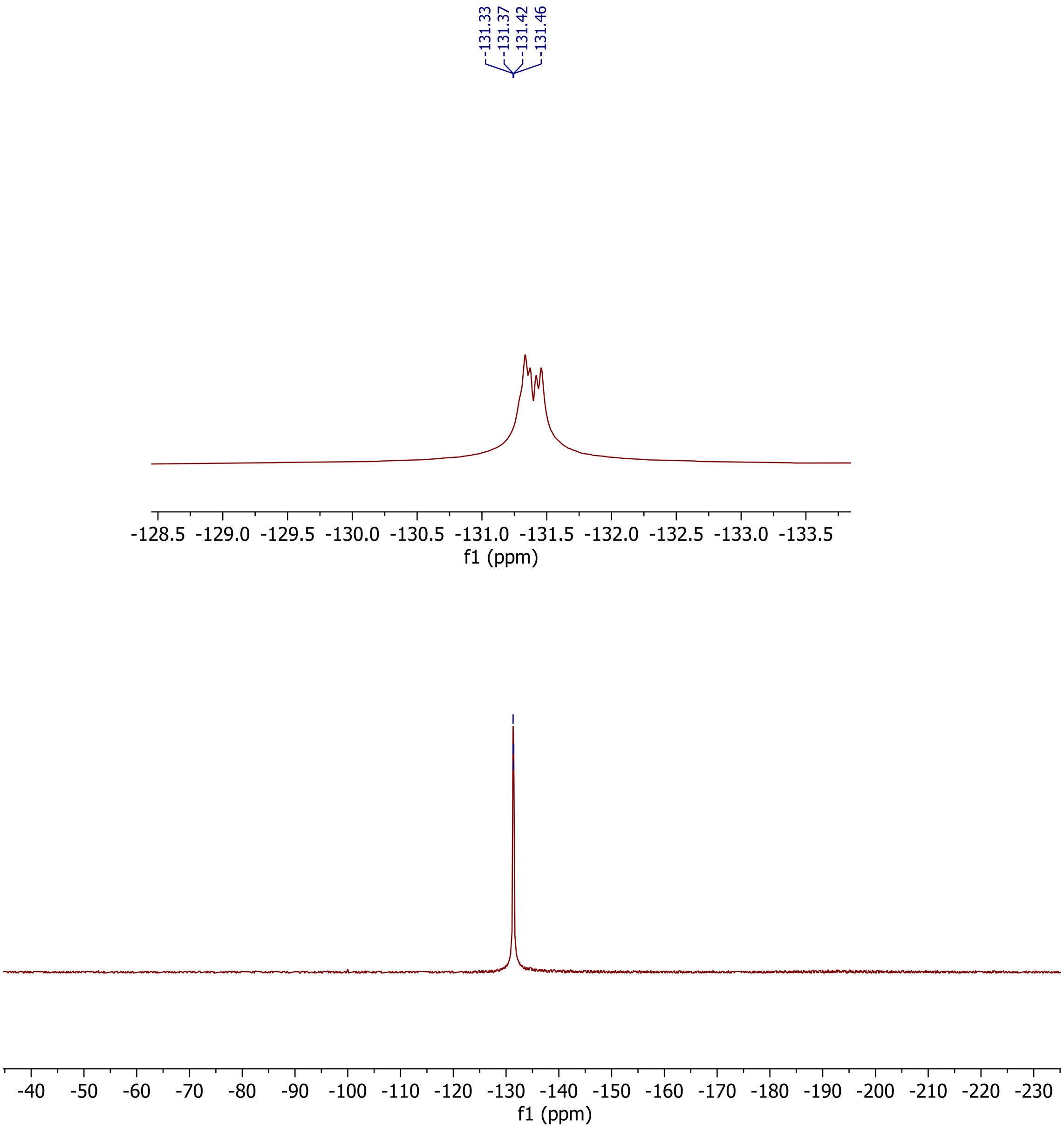

Figure S36. ^{19}F NMR spectrum of **CBF2-NO2** in CD_2Cl_2 at RT.

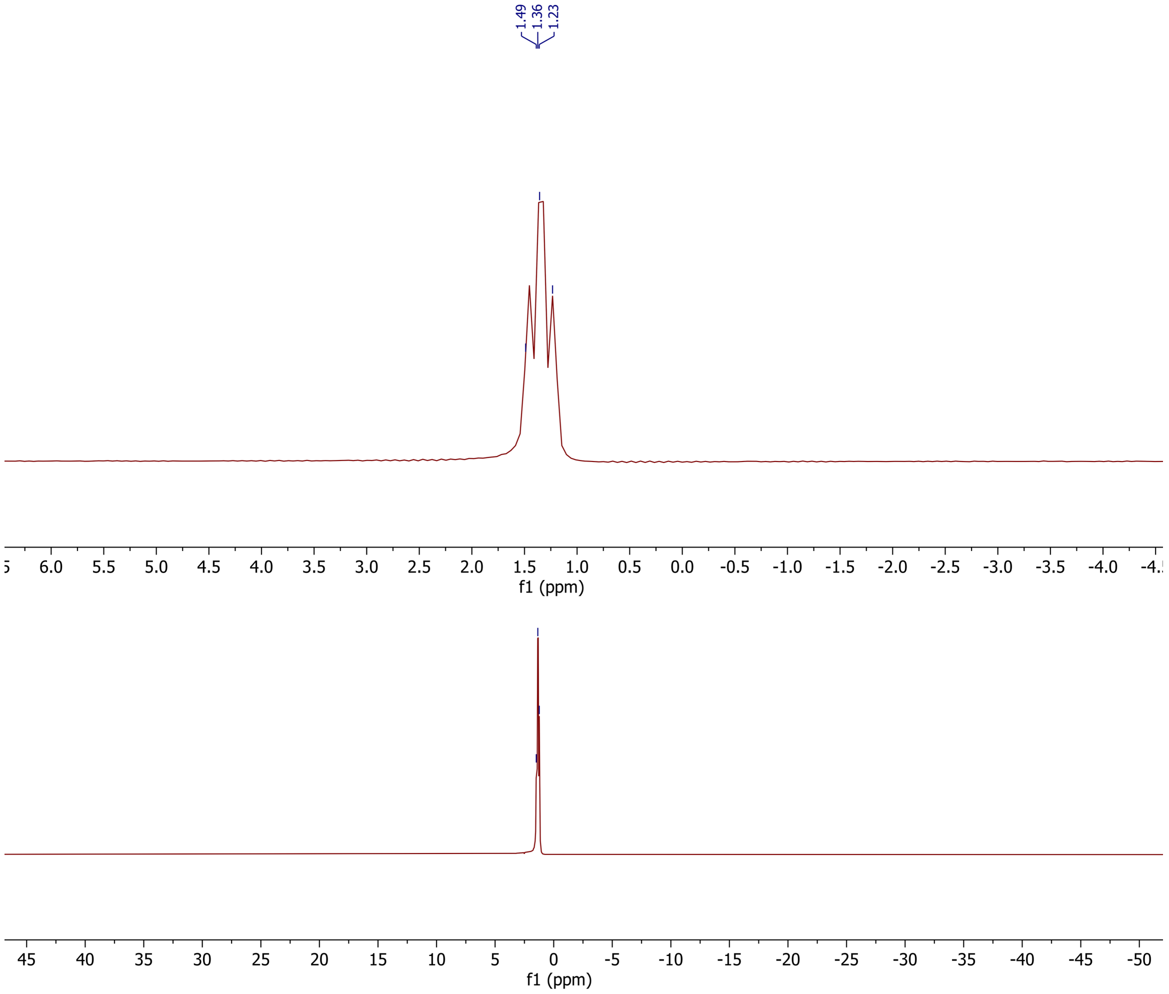

Figure S37. ^{11}B NMR spectrum of **CBF2-NO2** in CD_2Cl_2 at RT.

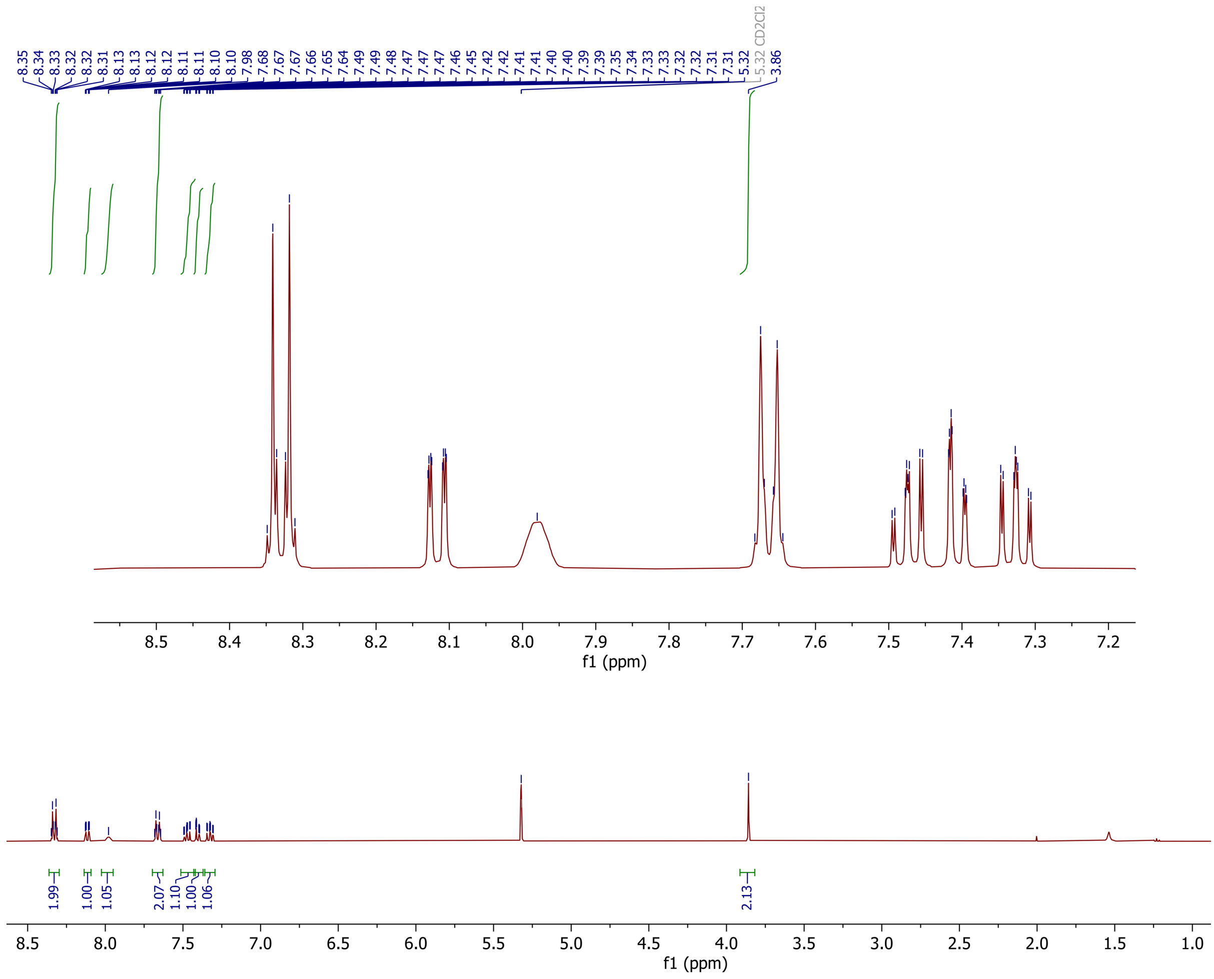

Figure S38. ^{1}H NMR spectrum of **SBF2-NO2** in CD_2Cl_2 at RT.

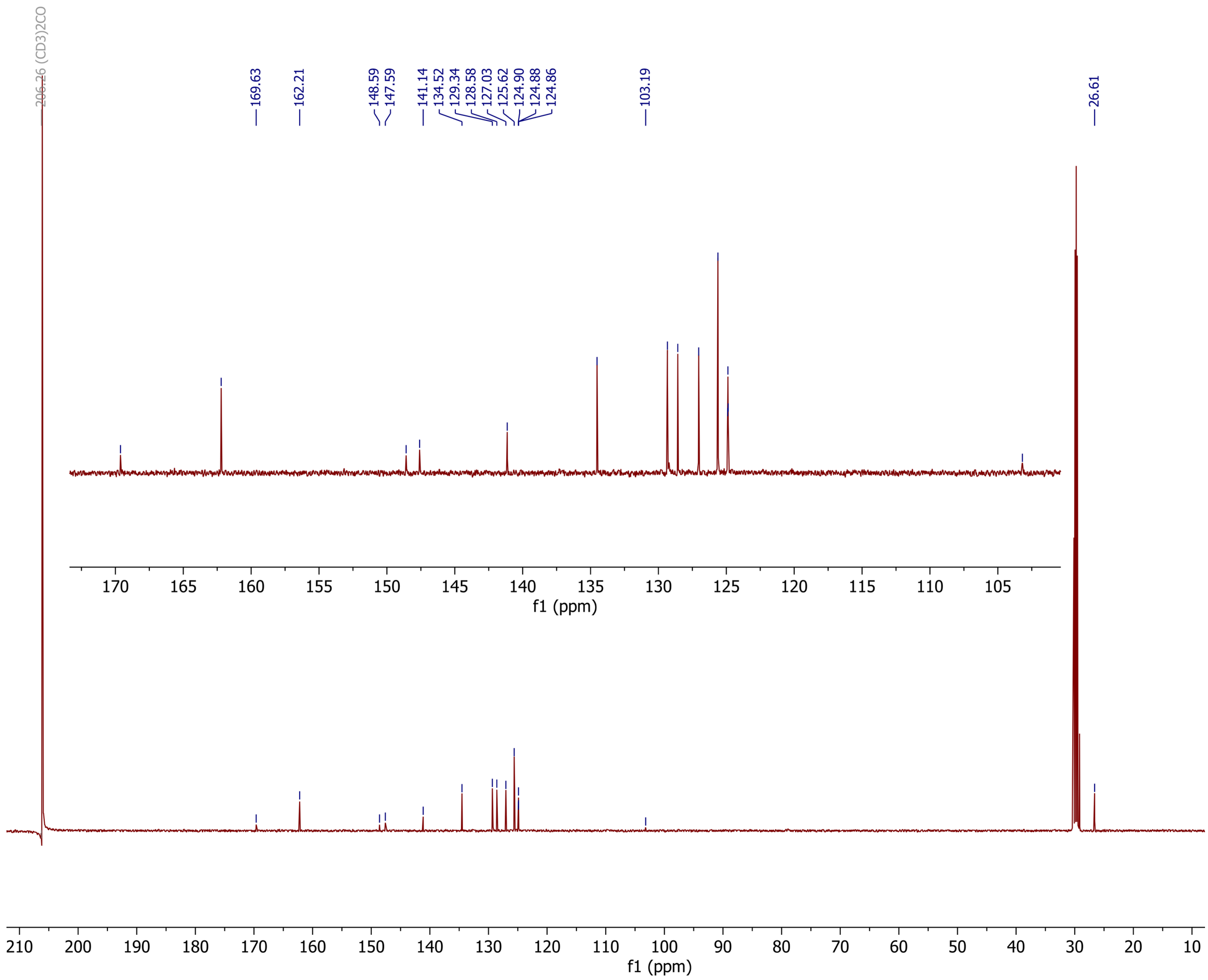

Figure S39. ^{13}C NMR spectrum of **SBF2-NO2** in acetone-d6 at RT.

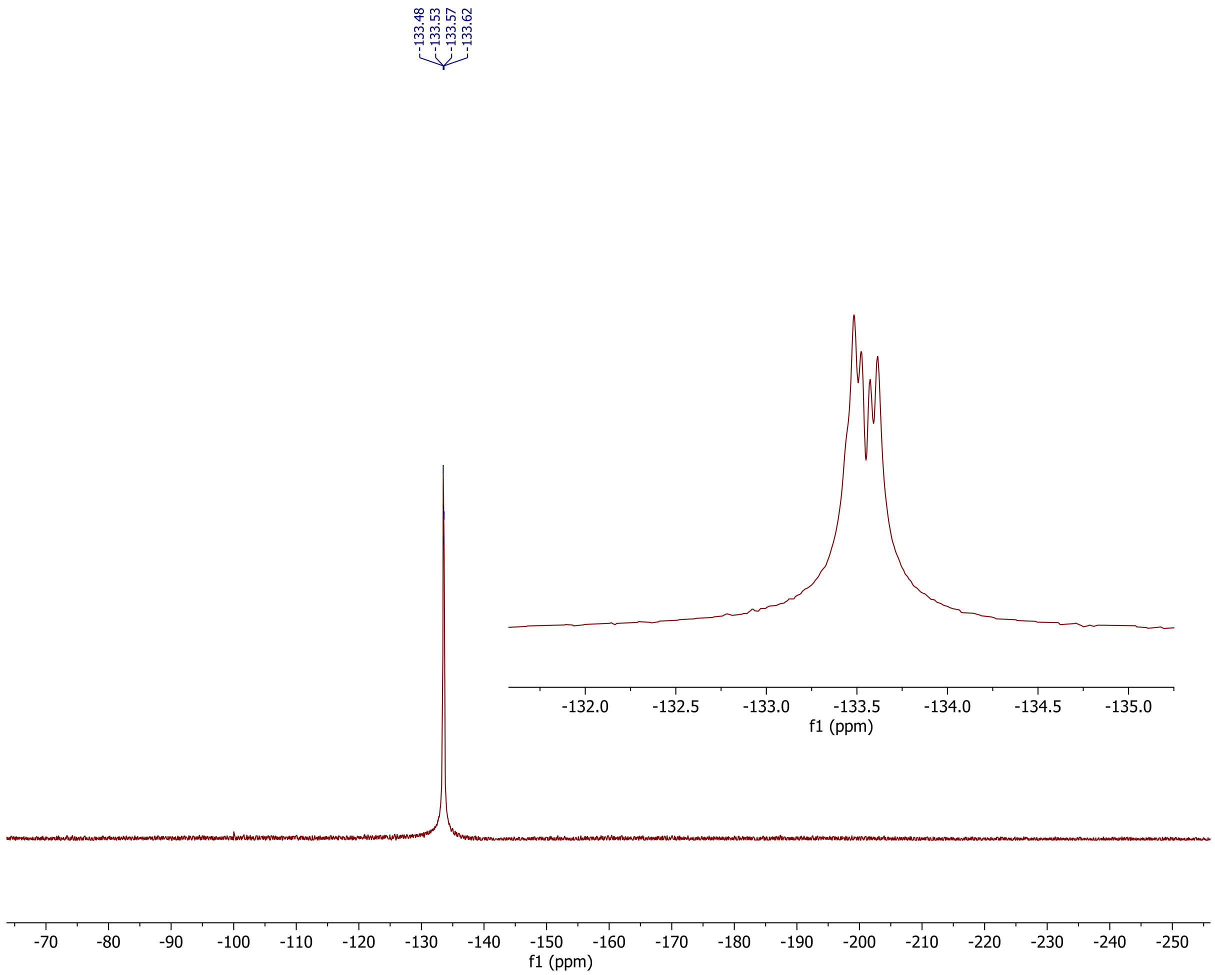

Figure S40. ^{19}F NMR spectrum of **SBF2-NO2** in CD_2Cl_2 at RT.

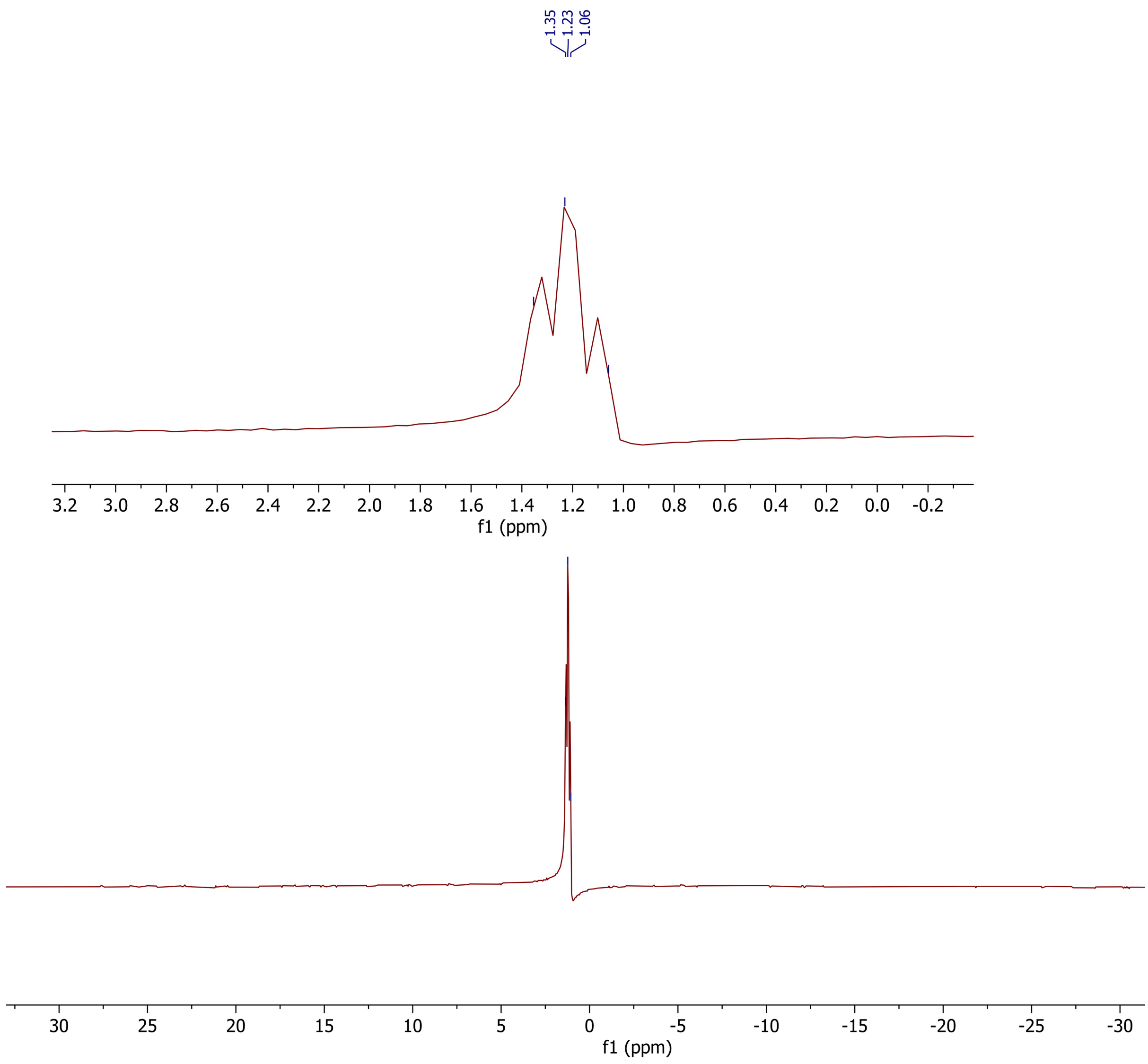

Figure S41. ^{11}B NMR spectrum of **SBF2-NO2** in CD_2Cl_2 at RT.

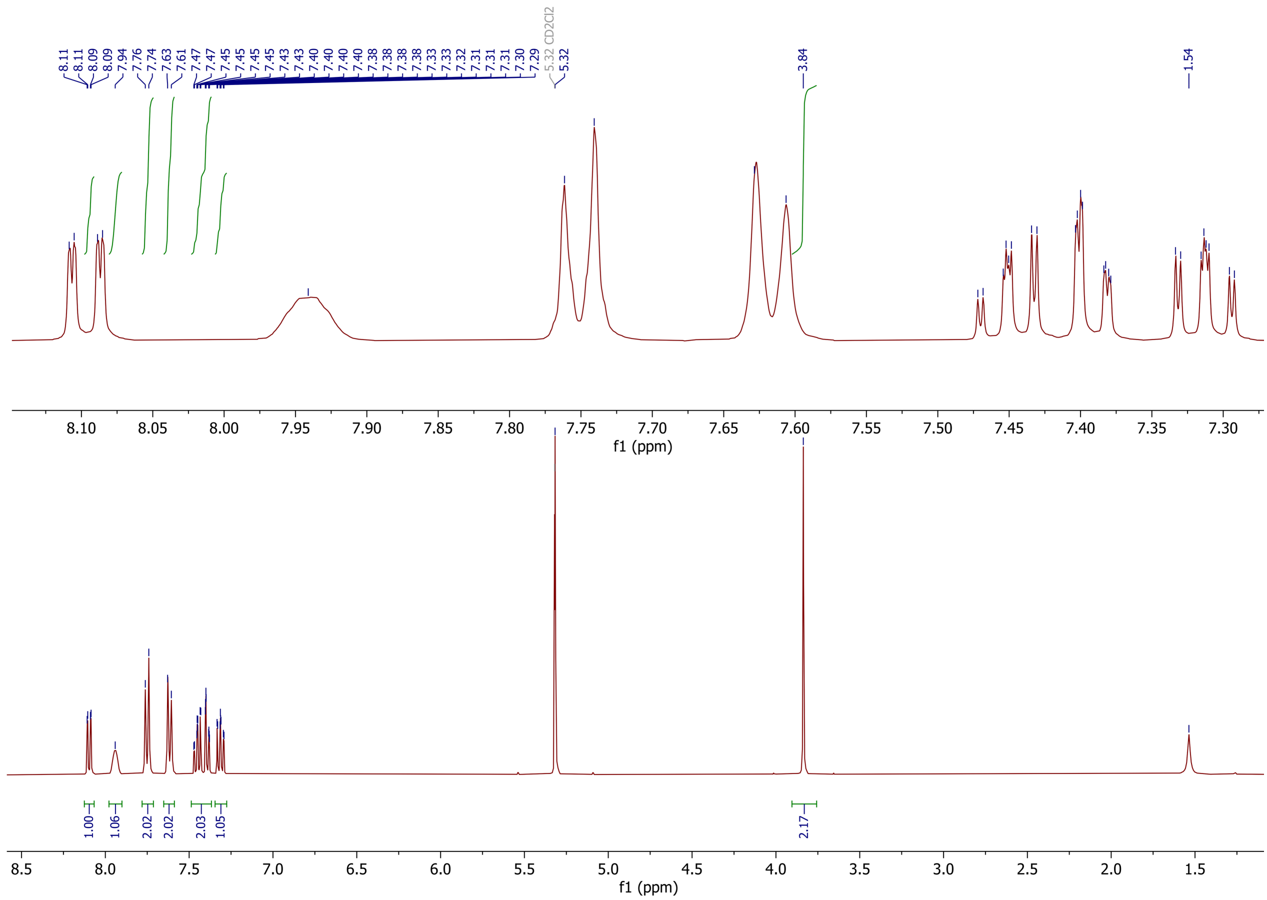

Figure S42. ^{1}H NMR spectrum of **SBF2-CF3** in CD_2Cl_2 at RT.

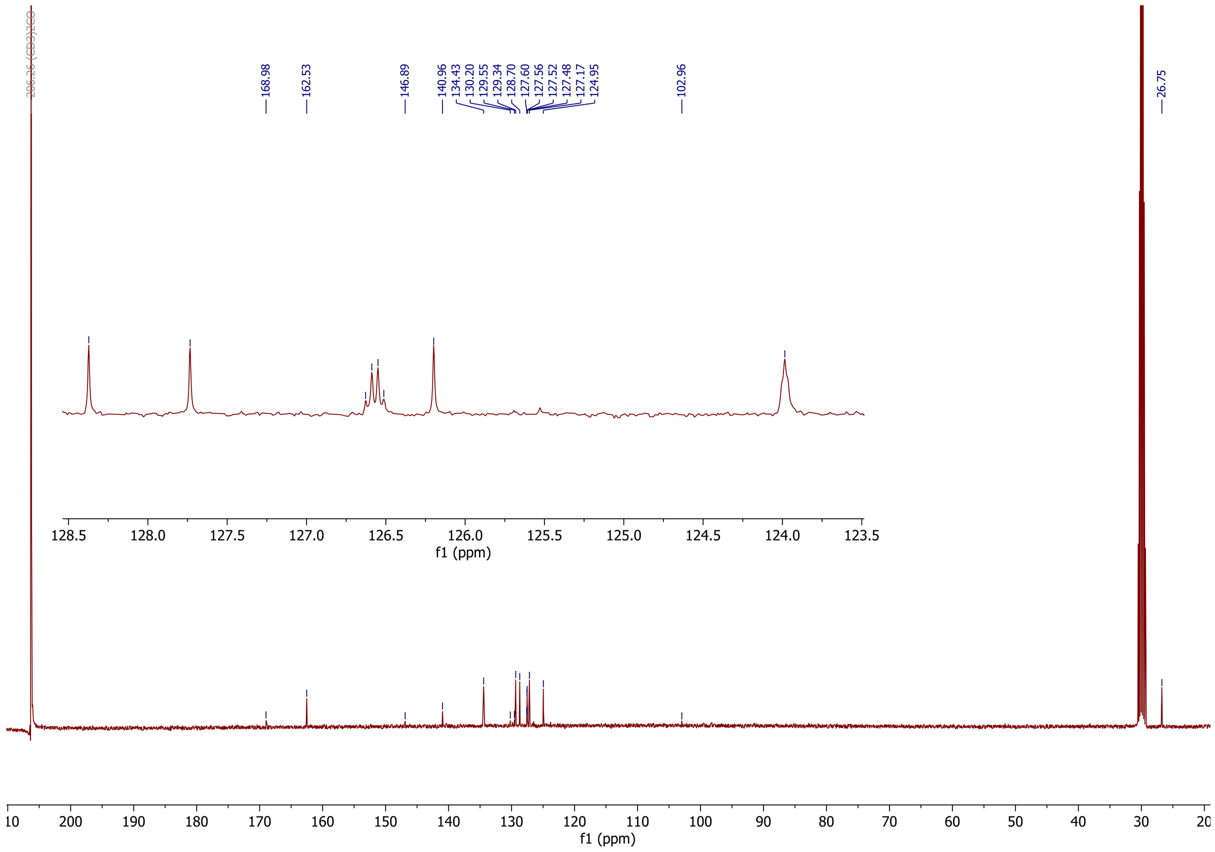

Figure S43. ^{13}C NMR spectrum of **SBF2-CF3** in acetone-d6 at RT.

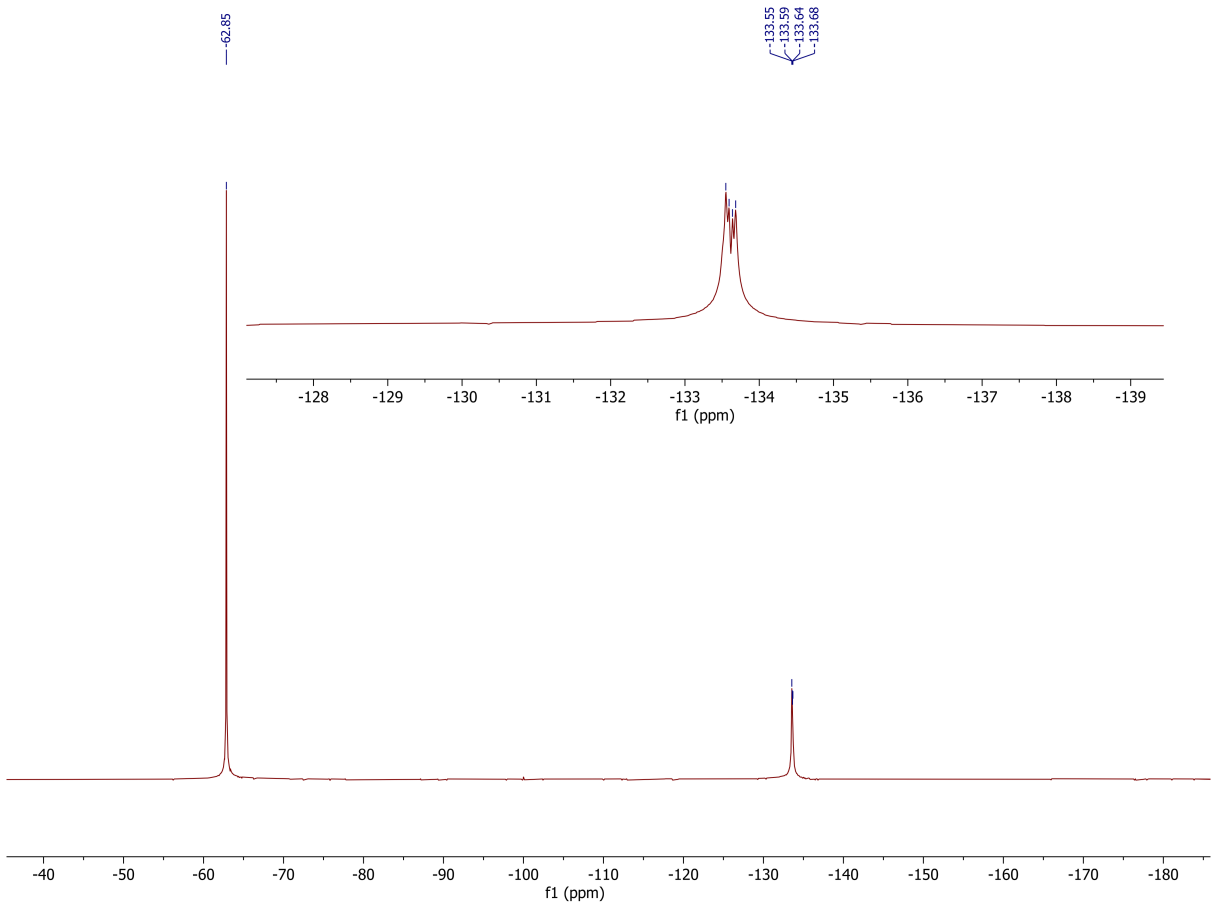

Figure S44. ^{19}F NMR spectrum of **SBF2-CF3** in CD_2Cl_2 at RT.

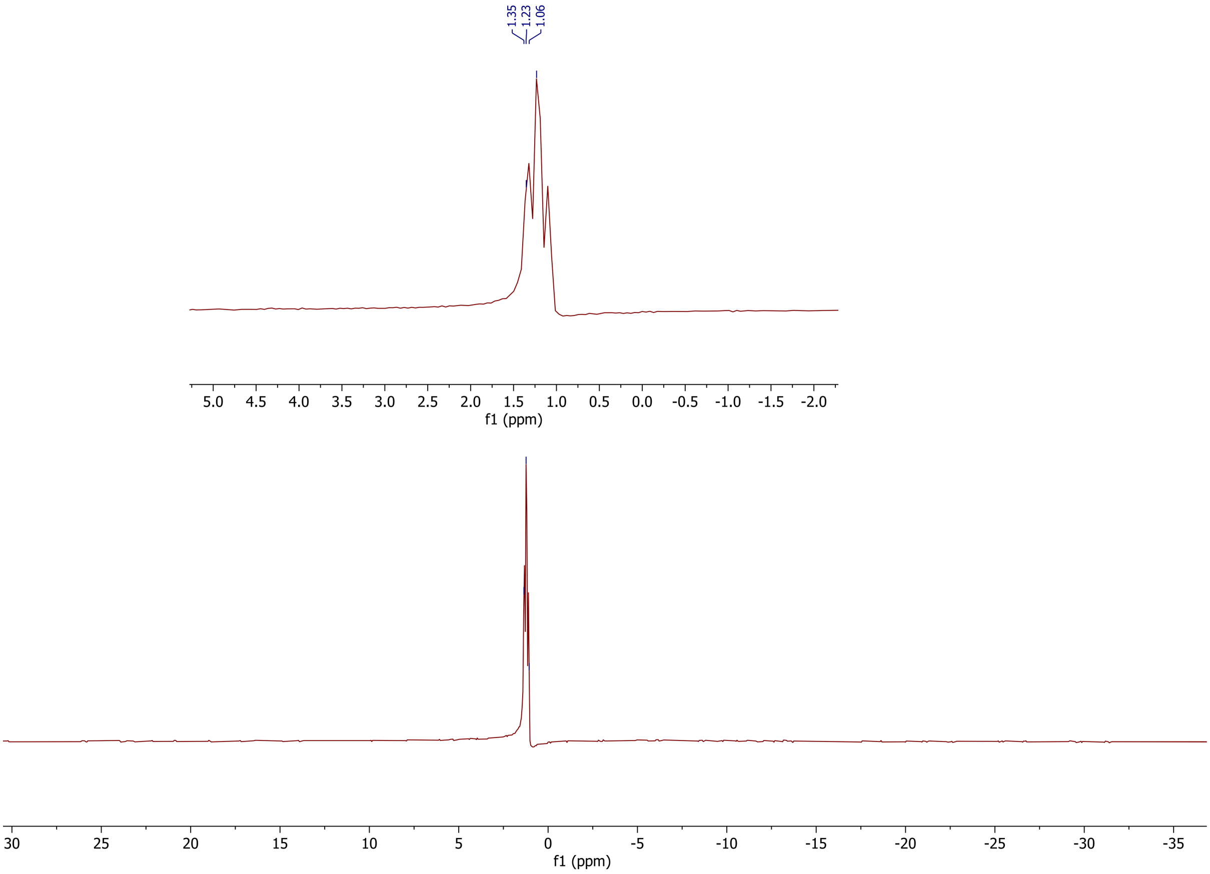

Figure S45. ^{11}B NMR spectrum of **SBF2-CF3** in CD_2Cl_2 at RT.

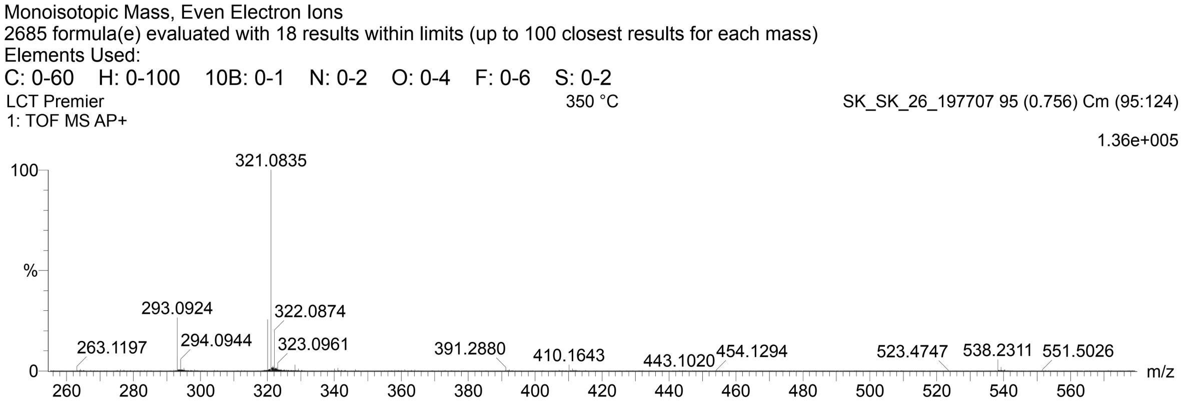

Figure S46. HRMS-APCI spectrum of **CBF2-NO2**.

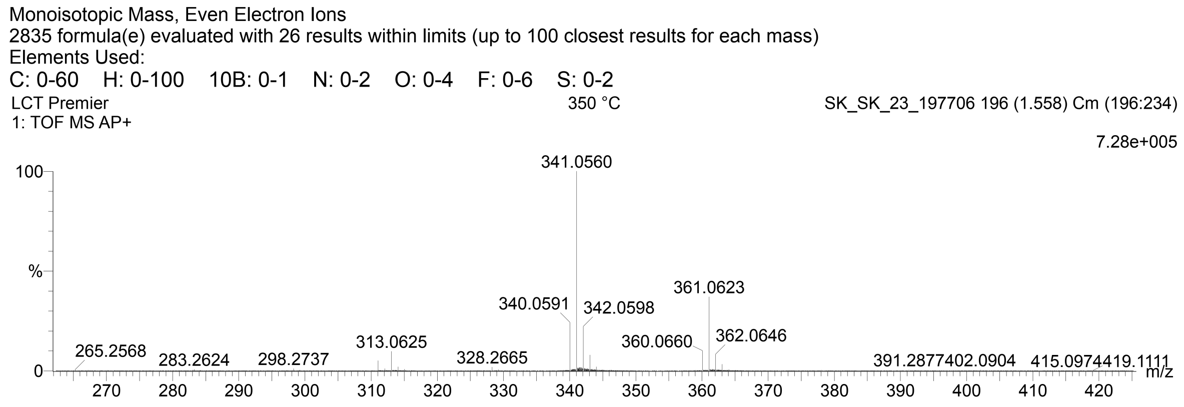

Figure S47. HRMS-APCI spectrum of **SBF2-NO2**.

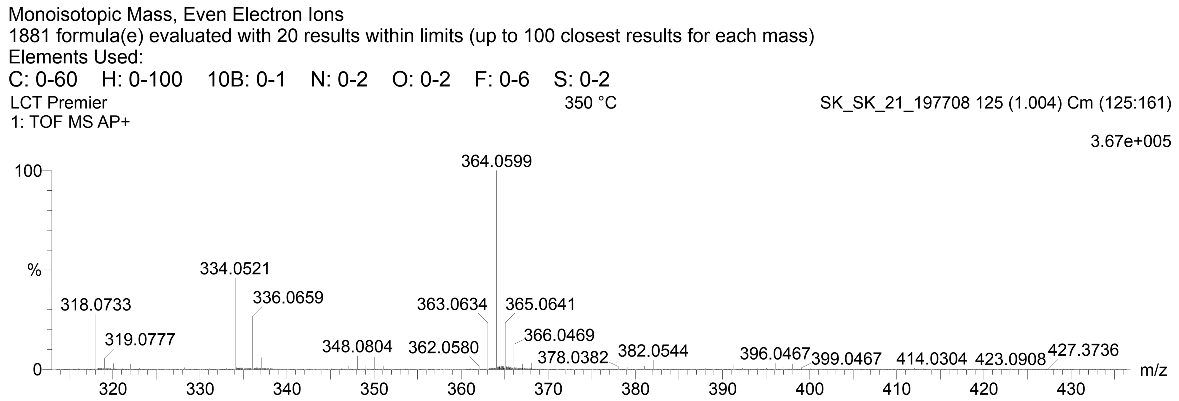

Figure S48. HRMS-APCI spectrum of **SBF2-CF3.**

5. References.

[41] Z. Yu, Y. Wu, L. Xiao, J. Chen, Q. Liao, J. Yao, H. Fu, *J. Am. Chem. Soc.* **2017**, *139*, 6376-6381.

[44] S. Li, Z. Yu, X. Xiao, H. Geng, K. Wang, X. Jin, Q. Liao, Y. Liao, Y. Wu, J. Yao, *Laser Photonics Rev.* **2019**, *13*, 1900036.

[45] S. Li, X. Jin, Z. Yu, X. Xiao, H. Geng, Q. Liao, Y. Liao, Y. Wu, W. Hu, H. Fu, *J. Mater. Chem. C* **2021**, *9*, 7400-7406.

[69] G. R. Fulmer, A. J. M. Miller, N. H. Sherden, H. E. Gottlieb, A. Nudelman, B. M. Stoltz, J. E. Bercaw, K. I. Goldberg, *Organometallics*, **2010**, *29*, 2176-2179.

[70] APEX5, v.2023.9-2, Bruker AXS Inc., Madison, WI, USA, **2019**.

[71] SAINT+ v8.40.0, Bruker AXS Inc., Madison, WI, USA, **2019**.

[72] SADABS, Bruker AXS Inc., Madison, WI, USA, **2019**.

[73] G. M. Sheldrick, *Acta Crystallogr. A* **2015**, *71*, 3-8.

[74] G. M. Sheldrick, *Acta Crystallogr. C* **2015**, *71*, 3-8.

[75] O. V. Dolomanov, L. J. Bourhis, R. J. Gildea, J. A. K. Howard, H. Puschmann, *J. Appl. Crystallogr.* **2009**, *42*, 339-341.
